# The influence of interpersonal synchrony and autism on impressions of dyadic interactions: a preregistered study

**DOI:** 10.1186/s13229-025-00668-y

**Published:** 2025-06-12

**Authors:** Irene S. Plank, Ralf Tepest, Kai Vogeley, Christine M. Falter-Wagner

**Affiliations:** 1 Department of Psychiatry and Psychotherapy, LMU University Hospital, LMU Munich, Nußbaumstraße 7, 80336 Munich, Germany; 2 Department of Psychiatry, Faculty of Medicine and University Hospital Cologne, University of Cologne, Cologne, Germany; 3 Research Center Jülich, Institute of Neuroscience and Medicine, Cognitive Neuroscience (INM-3), Jülich, Germany

**Keywords:** Impression formation, Interpersonal synchrony, Observed interactions, Autism spectrum disorder, Behavioural coordination, Dyadic interactions

## Abstract

**Background:**

Humans form almost instantaneous impressions of everyone they encounter. These impressions set the first tone for how they approach and interact with others. Research on impression formation unveiled that impressions formed by autistic and non-autistic people are often less favourable when rating an autistic person. This effect is partly explainable by differences in motion dynamics.

**Methods:**

In this preregistered study, we systematically assessed impressions formed by 27 autistic and 36 non-autistic comparison observers when watching videos showing silent interactions between either two non-autistic or between an autistic and a non-autistic person. We used an eye tracker to capture their gaze patterns while observing these interactions. Of each dyadic interaction, video vignettes with high and vignettes with low interpersonal synchrony of movement (IPS_mov_) were extracted using Motion Energy Analysis so that we could investigate the effects of interpersonal synchrony and diagnosis, respectively.

**Results:**

Interactions were rated less favourably when the observed dyad included an autistic adult. Additionally, interactions showing low IPS_mov_ were rated less favourably than interactions showing high IPS_mov_, regardless of dyad type. Both autistic and comparison observers rated interactions of non-autistic dyads and high IPS_mov_ interactions more favourably. Gaze patterns revealed differences between autistic and comparison observers, but no differences due to IPS_mov_ or dyad type. Furthermore, dwell times to hands predicted ratings.

**Limitations:**

In this study, we investigated specific influences on impression formation, specifically interpersonal synchrony of movement and autism. There are many more potentially interesting aspects of individuals that impact impression formation, such as facial expressiveness, gaze behaviour and linguistic content of conversations, which should be investigated systematically and in a controlled fashion in future research.

**Conclusions:**

Extending research on autism and impression formation to dyadic interactions, this study reveals that motion dynamics play a role in how pleasant interactions are perceived. Autistic-involved interactions were rated lower, despite observers being unaware of the dyad type and only watching people’s outlines. Future research should identify conversational aspects driving lower ratings of mixed dyads, potentially considering the effect of hand dwell times on ratings. Autistic and comparison observers showed different gaze patterns despite similar ratings, confirming distinct social information processing.

**Supplementary Information:**

The online version contains supplementary material available at 10.1186/s13229-025-00668-y.

## Background

### Autism spectrum disorder and interpersonal synchrony

Several psychiatric diagnoses are associated with changes in behaviour affecting social interactions [1]. Autism spectrum disorder (ASD) is characterised by differences in social interaction and communication, along with repetitive and restricted behaviours [2]. While symptoms of this neurodevelopmental disorder emerge during childhood, they persist across the lifespan profoundly affecting autistic adults’ lives [3]. A relevant aspect of behaviour that is affected in ASD is the coordination of behaviour with an interaction partner, i.e., interpersonal synchrony (IPS [4–8]). IPS is crucial for facilitating social interactions [9, 10]. Establishing social connections or rapport with others critically depends on experiencing smooth reciprocity in interactions with them [11–13]. For instance, IPS of movement (IPS_mov_) has been shown to be decreased in dyads consisting of two autistic interaction partners as well as in mixed dyads consisting of one autistic and one non-autistic interaction partner [5]. This effect of decreased IPS patterns can be traced back to attenuations in adaptation of the two interaction partners, i.e., leading and following behaviours. These patterns have been used to train machine learning algorithms which detected autistic interactions based on IPS and facial expressions as well as based on speech patterns and IPS of speech [7, 14]. Despite reports of differences on the production of IPS, imaging studies revealed that processing of IPS appears to be associated with similar neural correlates in autistic and non-autistic adults when they observe an interaction [15] or when they are part of an interaction [16]. Bierlich and colleagues [15] collected ratings of the naturalness of the observed interactions, which did not differ between autistic and non-autistic comparison observers, possibly indicating that perception and interpretation of IPS_mov_ is spared in ASD.

### Impressions of autistic people formed by other people

Several impression formation studies have shown that autistic people receive less favourable ratings by others than non-autistic people, but underlying mechanisms are unclear. Several studies have asked participants to watch videos of others talking and to report their impressions, including on the observed persons likeability, awkwardness or trustworthiness. Observed autistic people have been consistently rated less favourably [17–22]. Less favourable impressions have potentially vast implications for social [19, 20] and professional participation of autistic adults [23]. Based on reported differences in IPS associated with ASD, a recent study investigated the effect of IPS_mov_ on impressions formed about autistic and non-autistic people by non-autistic observers [24]. Specifically, silent video segments of dyadic interactions either between two non-autistic or including one autistic person were presented to non-autistic observers who were asked to rate their impressions of one of the two interaction partners. IPS_mov_ was evaluated as either leading or following behaviour to assess the differential influence on impressions of just one of the two interaction partners. Results showed that leading results in more favourable impressions of non-autistic people only: impressions of autistic people were not influenced by IPS_mov_. This effect suggests that autistic people did not benefit from the positive effect of IPS_mov_ even when they interacted with non-autistic people. Another possible explanation for less favourable ratings is readability: in a sample of non-autistic observers, Alkhaldi and colleagues [25] found a relationship between independently rated readability and impressions, such that people regardless of ASD diagnosis were rated more favourable by non-autistic observers the easier they were to read.

### Impressions of other people formed by autistic observers

Reports on differences between impressions formed by autistic and non-autistic participants showed mixed results, with some findings showing more favourable impressions being formed by autistic adults of virtual characters [26] and people based on brief videos [18]. Other studies with virtual characters who were modelled in their behaviour after autistic and non-autistic people showed more positive ratings by non-autistic people [27]. Focusing on the interaction between diagnostic status of the observer and the observed, DeBrabander and colleagues [18] asked autistic and non-autistic adults to rate brief videos of autistic and non-autistic adults. They used multiple ratings of character impressions, i.e., likeability, and socialisation intentions, i.e., wanting to hang out with this person. While both autistic and non-autistic observers rated autistic observed people as less favourable on half of the character impressions and three of the four socialisation intentions, there was less difference between the intentions to hang out with a person dependent on their diagnostic state in autistic observers. Additionally, while non-autistic observers reported that they were more likely to sit near non-autistic people, they were equally likely as autistic observers to sit near an autistic person. This data shows that less favourable impressions of autistic people are not limited to non-autistic observers but also extend to autistic observers.

### Impressions of dyads including an autistic person

Since interactive behaviour influences the impression formed of the interaction partners, we expect interactive behaviour to also influence impressions of the interaction itself. In a study by Crompton and colleagues [28], non-autistic, autistic and mixed dyads completed social interactions and reported their feelings of rapport on five dimensions: ease, enjoyment, success, friendliness and awkwardness. These dimensions with awkwardness being reverse coded were then combined to a single rapport score and the interaction partners’ scores were averaged to compute a rapport score for the interaction. The authors found evidence for higher mean rapport reported in the non-autistic dyads, followed by the autistic dyads, with the mixed dyads reporting the lowest rapport values. Afterwards, they asked an independent sample of autistic and non-autistic participants to report their impression of the dyads’ rapport based on observing some of the conversations via video recordings, with rapport again being comprised of ratings on how easy, enjoyable, friendly, successful and awkward the observers considered the interaction. The authors found evidence for impressions of higher rapport for the non-autistic and the autistic dyads compared to the impressions of the mixed dyads, with comparable results for autistic and comparison observers. This pattern of results could suggest that while impressions formed of autistic people by observers are less favourable, this does not necessarily translate to less perceived rapport of observed interactions including autistic people. Crompton and colleagues [28] postulate this might be due to rapport impressions being based on stable interpersonal coordination in their study, which might be increased in homogeneous dyads due to more similar interpersonal styles.

### Gaze patterns to assess social attention

Social interactions are complex stimuli allowing the selection of salient aspects to which one attends most strongly. While one person might focus on the produced gestures, another might attend to the faces and another might constantly shift their attention between the interaction partners. Capturing gaze patterns offers some insights into these attentional differences and allows us to assess mechanistic differences between autistic and non-autistic observers [29]. Previous research has shown differences in processing of observed interactions [15] and attention to social stimuli between autistic and non-autistic people, for example in the form of decreased fixation duration to faces in general and the eye region in particular in autistic adults [30]. Literature linking gaze patterns to influences of autism on impression formation is scarce. Grossman and colleagues [31] used brief video clips of autistic and non-autistic adolescents retelling an adventure story to capture impressions and gaze patterns of autistic and non-autistic observers. The videos showed the story-teller from mid-chest upwards but no other person. While there were no differences in gaze patterns between autistic and non-autistic observers, both observer groups differed in their gaze patterns to autistic and non-autistic story tellers. Authors did not link the gaze patterns to the impressions formed by the observers. Similarly, gaze patterns of autistic and non-autistic observers did not differ in a study where they were asked to judge dominance and trustworthiness of 2D models of male faces [32]. However, despite the wealth of research on social attention in autism, the links between social attention attenuations in autism and impressions of dyadic interactions as well as between attention in general and impressions of dyadic interactions have not been explored yet.

### Purpose of this study

While past research has shown that attenuated IPS is an important characteristic of ASD and that impressions can be influenced by IPS_mov_, the link between perceived IPS_mov_ achieved by interaction partners together and autism is unclear. In Plank et al. [24], we observed an interaction effect between IPS_mov_ and diagnostic status of a partner in a dyadic interaction on impression ratings of this partner; however, we only captured ratings of non-autistic participants and did not collect detailed gaze patterns due to the study being conducted online. Extending this research to autistic participants and capturing gaze patterns allows us to investigate the mechanisms behind the link between IPS_mov_ and impressions. Furthermore, while Plank et al. [24] explored the link between IPS_mov_ and impressions on the level of the individual, the current study focuses on IPS_mov_ as a feature of the dyad since IPS_mov_ always depends on the movement patterns of both interaction partners. Therefore, the goal of this study was to investigate the influence of IPS_mov_ in interactions of autistic and non-autistic people on impressions of the interaction itself formed by a new sample of autistic and non-autistic comparison observers. We presented participants with video segments of interactions of non-autistic or mixed dyads portraying either high or low IPS_mov_ which was extracted from the videos using Motion Energy Analysis [33]. After each vignette, we asked participants to rate how pleasant they find the just observed interaction. Additionally, we collected eye-tracking data to extract dwell times and assess whether autistic and comparison observers focus on the same aspects of the observed interactions. We hypothesised that ratings would be more positive for interactions of non-autistic dyads than interactions of mixed dyads as well as more positive for interactions with high compared to low IPS_mov_. Furthermore, we expected differences between the ratings given by autistic and comparison observers. Specifically, we expected a decreased effect of dyad type on the ratings of autistic compared to comparison observers. Last, we hypothesised a decreased effect of IPS_mov_ for mixed dyads compared to non-autistic dyads. Concerning the dwell times, we expected differences between the dwell times on areas of interests (AOIs) between non-autistic and mixed dyads, between interactions with high and low IPS_mov_ as well as between autistic and comparison observers.

## Method

This study was preregistered on the Open Science Framework: https://osf.io/36tuk. The experiment was presented using PsychToolBox 3 [34] in MATLAB R2023a [35]. Data was preprocessed and analysed using MATLAB R2023a and R 4.2.2 in RStudio 2024.0.4 [36]. All scripts as well as anonymised, aggregated data can be retrieved from GitHub: https://github.com/IreneSophia/PESI. Variance is reported in standard deviations throughout the manuscript and credible intervals are reported in square brackets, unless indicated differently.

### Participants

This study was approved by the Ethics committee of the Medical Faculty of the LMU Munich (Reference number: 23–0268). Participants were recruited through the internal participant database and local network. We aimed to analyse at least 27 participants per group to achieve a power of 90% to detect a medium group effect (*d* = 0.45). The power analysis was conducted with PANGEA v0.2 [37] based on a mixed design with three population-level predictors (observers: ASD or non-autistic comparison group COMP; dyad type: mixed or non-autistic; IPS_mov_: high or low) and two group-level predictors (participants and trials). Inclusion criteria were as follows: no current neurological diagnoses, between 18 and 45 years old, no intellectual disability (i.e., intelligence estimate above 70), normal or corrected-to-normal vision and written informed consent. Additionally, autistic participants had an existing ASD diagnosis, while comparison participants had no psychiatric diagnoses. Existing ASD diagnoses according to national health guidelines were verified either before being scheduled for testing or at the beginning of testing. We excluded data of seven participants from analysis, five autistic and two comparison participants, because only less than two thirds of the ratings were completed. Due to technical difficulties, only data of 46 participants were included in the analysis of the eye-tracking data (20 ASD and 26 COMP), deviating from the preregistration, while a total of 63 participants’ data were included in the behavioural analysis (27 ASD and 36 COMP). The number of trials in the remaining sample was 59.9 ± 0.93 out of 64 trials in the autistic and 60.1 ± 0.92 trials in the comparison group which did not differ credibly between groups (log*BF*_*10*_ = -1.192). Furthermore, the observer groups were comparable in age, gender distribution and IQ (see Table 1).

**Table 1:** Sample characteristics. The table shows sociodemographic information and questionnaire values for both observer groups separately. We used bayesian *t*-tests to compare groups with values violating the normality assumption being rank-transformed. The exception is gender distribution for which we used a bayesian contingency table. Positive log-transformed Bayes factors indicate evidence in favour and negative evidence against a group difference

Measurement	Behavioural sample			Eye-tracking sample		
	ASD	COMP	logBF_10_	ASD	COMP	logBF_10_
Age	28.59 (± 6.95) [18–44]	27.69 (± 5.66) [19–41]	-1.30	28.75 (± 5.93) [20–39]	27.38 (± 5.25) [19–37]	-0.95
BDI	17.44 (± 12.87) [1–53]	4.86 (± 4.93) [0–21]	12.16	13.90 (± 12.54) [1–53]	5.65 (± 5.20) [0–21]	2.37
Gender^1^	11–16	19 − 17	-0.75	10–10	17 − 9	-0.52
d2 - performance	101.74 (± 13.76) [77–130]	104.28 (± 10.04) [80–130]	-0.98	100.90 (± 14.04) [80–130]	103.81 (± 8.50) [87–120]	-0.89
d2 - speed	102.04 (± 12.74) [77–123]	104.69 (± 9.85) [87–130]	-0.94	101.45 (± 13.71) [80–123]	104.69 (± 10.47) [87–130]	-0.92
IQ estimate	113.59 (± 21.03) [77–145]	114.58 (± 12.98) [77–136]	-1.33	113.50 (± 20.84) [77–145]	115.12 (± 11.65) [85–136]	-1.18
Ishihara	28.96 (± 2.38) [18–30]	28.25 (± 3.43) [11–30]	-0.19	28.90 (± 2.73) [18–30]	28.62 (± 1.96) [21–30]	0.03
RADS-R	31.19 (± 8.37) [6–39]	7.44 (± 5.59) [1–29]	28.41	31.95 (± 9.14) [6–39]	6.54 (± 4.20) [1–15]	19.93
STAI-trait	52.15 (± 11.52) [27–72]	30.89 (± 9.58) [11–50]	18.78	50.05 (± 12.14) [27–72]	30.62 (± 9.62) [17–50]	10.63
UI	65.63 (± 13.24) [22–86]	37.97 (± 10.52) [24–67]	21.42	64.95 (± 13.03) [22–86]	34.85 (± 8.76) [24–67]	14.55

Note. Means are presented together with standard deviations and ranges in square brackets. ASD = Autism Spectrum Disorder; BDI = Beck Depression Inventory; BF = Bayes Factor; COMP = comparison group of non-autistic observers; IQ = intelligence quotient; RADS-R = Ritvo Autism Diagnostic Scale– Revised; STAI = State and Trait Anxiety Inventory; UI = Intolerance for Uncertainty; ^1^ first, counts of female, then of male participants. There were no participants who chose any other gender label in the current study

### Procedure

Participants completed the interaction observation task to assess the here presented hypotheses. Additionally, they completed two unrelated tasks, a task modelled after Allenmark and colleagues [38] and a visual acuity task [39], which will be presented elsewhere. The order of tasks was counterbalanced across participants. They completed a demographic questionnaire as well as the following standardised questionnaires and tests: the Beck Depression Inventory (BDI-II [40]), the Culture Fair Intelligence Test 20-R (CFT 20-R [41]),, the d2 attention test [42], the questionnaire for Intolerance for Uncertainty (UI [43]), the Ishihara test for colour blindness [44], the Landolt C for vision acuity [45], the Ritvo Autism Diagnostic Scale– Revised (RADS-R [46]), as well as the State and Trait Anxiety Inventory (STAI [47]). The interaction observation task was presented using a Lenovo Thinkpad P52 laptop with an external monitor (1920 × 1080 pixels, 533 × 300 mm, 120 Hz) and an external number pad. During the interaction observation task, eye movements of both eyes were captured with a LiveTrack Lightning eye tracker (Cambridge RS, sampling rate 500 Hz) while participants placed their head in a headrest to ensure a stable viewing distance of 57 cm. The eye tracker was calibrated before each task block using nine points and data was only collected if at least one eye had an accuracy below 0.5°. For the analysis of the fixations, we focused on the eye with the better accuracy during calibration for each participant. In total, testing took two to three hours.

### Stimulus creation

In the interaction observation task, we used silent videos of dyadic interactions either between two non-autistic adults or between an autistic and a non-autistic adult (mixed dyad) as captured by Georgescu and colleagues [5]. In this study, conversations of autistic and matched comparison adults between 23 and 56 years of age were video-recorded. Diagnoses according to national health guidelines and based on ICD-10 criteria were confirmed for all autistic participants in this study by two experienced clinicians [48]. While this study also included dyads of two autistic participants, we decided against including a third dyad type to keep the interaction observation task at an acceptable length for observers. In the video-taped conversations, the interaction partners jointly planned a five-course meal of foods and drinks they both dislike. Motion Energy Analysis (MEA) [33] was extracted from the head region and processed using the rMEA package, similar to previous work [5, 7]. First, MEA values were rescaled and then IPS_mov_ was computed using windowed-lagged cross-correlation using a window size of 10s and a lag of 3s. We focused on the peak cross-correlation value to characterise IPS_mov_.

After extraction of IPS_mov_ values, the videos were greyscaled and processed with a Gaussian filter to reduce the visual presentation to the outlines of the interaction partners, both anonymising the dyads and reducing information to the interaction partners’ movements. This filter removed characteristic features commonly associated with gender expression. Thus, gender of the interaction partners is no longer clearly recognisable; however, all dyads were matched for gender in the study by Georgescu and colleagues [5].

**Fig. 1:**
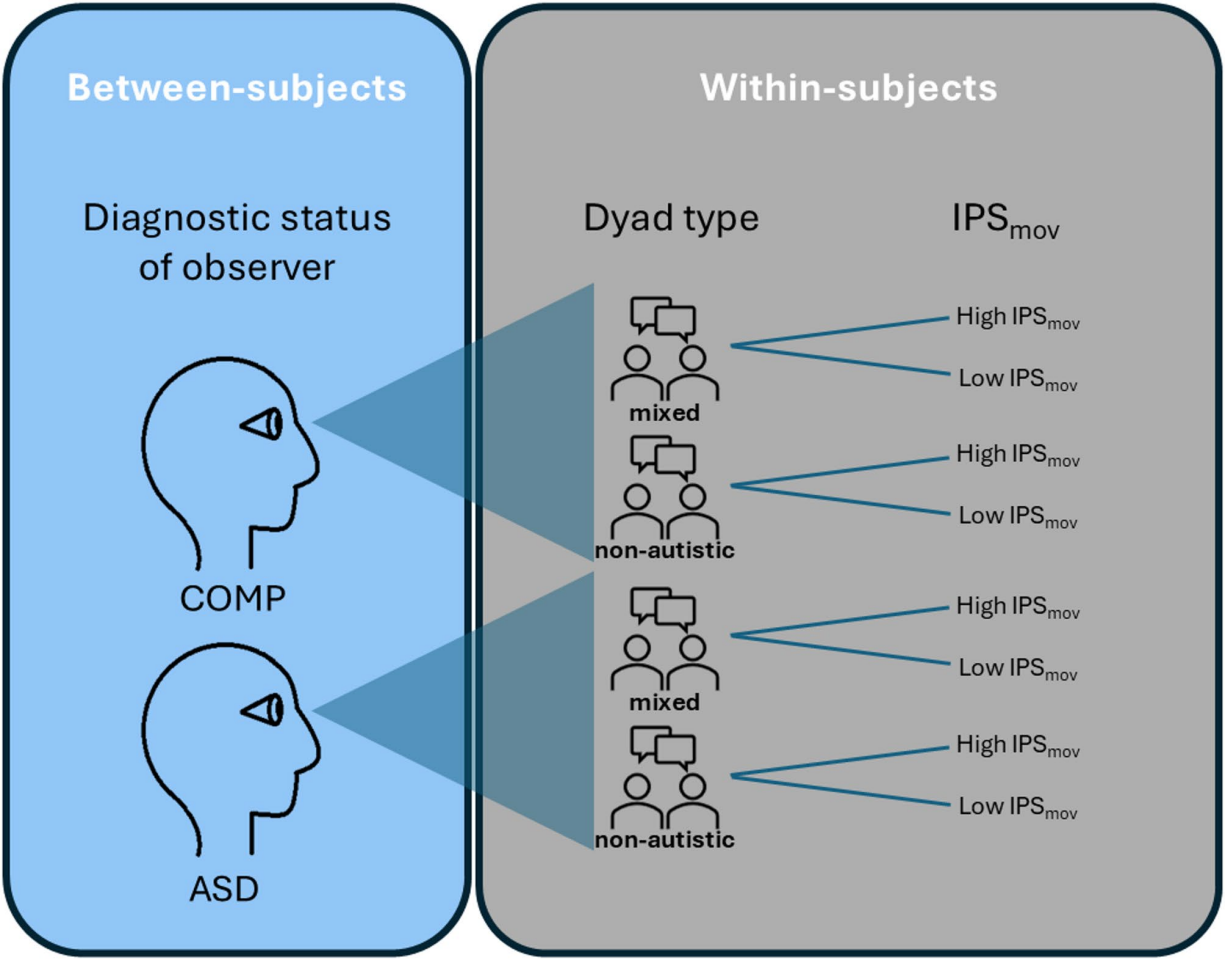
Schematics of the mixed 2 × 2 × 2 design. Autistic and non-autistic comparison (COMP) observers watched segments of four types: mixed dyad producing high IPS_mov_, mixed dyad producing low IPS_mov_, non-autistic dyad producing high IPS_mov_ and non-autistic dyad producing low IPS_mov_

Each stimulus captured a 10-second segment of these interactions. The segments were chosen from eight dyads, four mixed and four non-autistic, to create a balanced sample of segments portraying high and low IPS_mov_ of both mixed and non-autistic dyads such that there were no differences in overall motion. First, we excluded all 10-second segments outside of the middle 50% of each video in terms of overall motion based on MEA. Second, we chose the four segments with the highest and the four segments with the lowest IPS_mov_ of each dyad to model effects of IPS_mov_ independent of dyadic confounders, resulting in 64 segments in total (16 for each dyad type and IPS_mov_ combination). In the resulting 64 segments, segments of mixed and non-autistic dyads do not differ in IPS_mov_ (X̄_non−autistic_ = 0.53 ± 0.27, X̄_mixed_ = 0.52 ± 0.29, log*BF*_*10*_ = -1.365) or overall motion (X̄_non−autistic_ = 0.52 ± 0.08, X̄_mixed_ = 0.53 ± 0.06, log*BF*_*10*_ = -1.365) based on Bayesian *t*-tests. As a consequence, the task design consisted of one between-subjects factor (autistic and comparison observers) and two within-subject factors, namely IPS_mov_ and dyad type (see Fig. 1). In the two previous studies where the videos were used, stimulus choice was performed based on different criteria specific to the respective research question resulting in only 7.8% of overlap between the stimuli [15, 24]. While we cannot share the stimuli due to data protection, we have created mock stimuli which can be viewed here: https://github.com/IreneSophia/PESI/tree/main/mock-stimuli.

### Interaction observation task

**Fig. 2:**
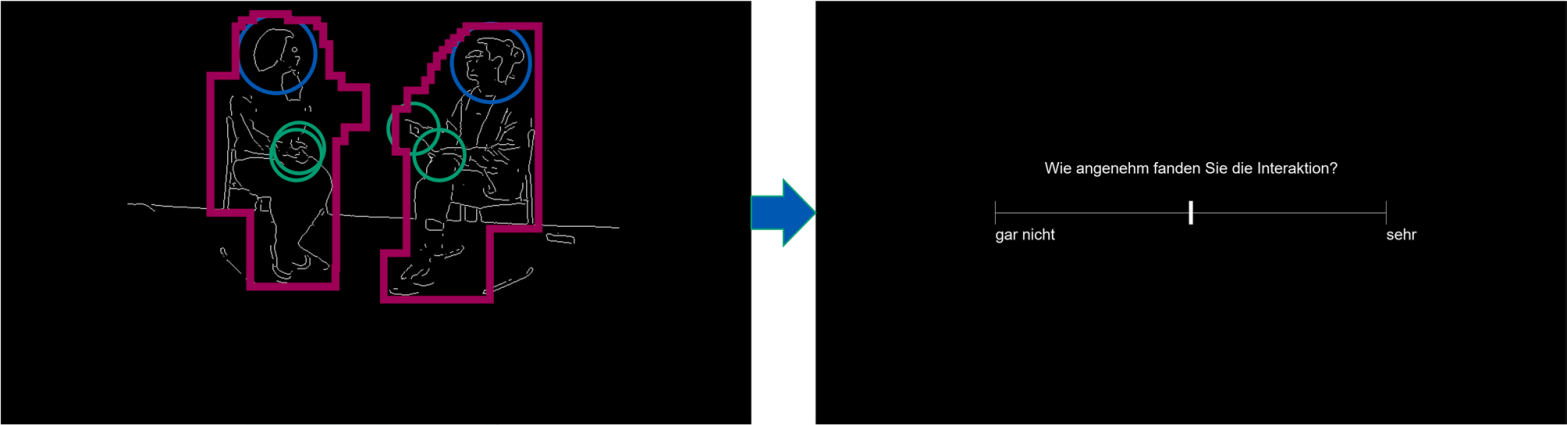
A still frame from a video segment, overlayed with the body (pink), hands (green) and head (blue) areas of interest (AOIs), followed by the impression rating. This segment was created with lab members to maintain the privacy of the interaction partners. It was processed with the same filter as the original stimuli.

In total, 64 segments were presented to each participant in two blocks. Participants were able to take a break between blocks. Each frame of the video segment was presented with an approximate visual angle of 26° by 18°, depending on the exact frame. Participants were instructed to watch videos of dyadic interactions without any further information on the interactions, neither that some include an autistic individual nor about the context of the conversations (for the instructions, see supplementary materials, p. 1). Before each segment, a fixation cross was presented for 50ms at the centre of the segment presentation. After each video segment, participants were asked to rate how pleasant they imagined the interactions to be (“Wie angenehm stellen Sie sich diese Interaktion vor?”, from “not at all” to “very”; see Fig. 2). Participants had 4s to complete their rating, if they did not complete their choice the trial was excluded from the analysis. Ratings were converted into values from 0 to 100.

To create frame-by-frame areas of interest (AOIs) dynamically tracking the heads and hands of the interaction partners, we used DeepLabCut™ which allows to estimate pose of user-defined body parts using deep learning [49]. AOIs were circle-shaped and had a diameter of 210 pixels for the heads and 140 pixels for the hands, corresponding to visual angles of 5.85° and 3.91°, respectively. Body AOIs were the same for all segments of one dyad and chosen to capture all possible body positions (see Fig. 2).

### Preprocessing and analyses

To improve model fit, we aggregated ratings, resulting in two values per participant for each dyad: the mean rating for segments of this dyad with high IPS_mov_ and the mean rating for segments with low IPS_mov_. Fixations and saccades were detected using the algorithm developed by Nyström and Holmqvist [50]. We classified for each fixation whether its starting point was located on a head, hand or body AOI to compute dwell times for each of the AOIs by summing fixation durations. In case of overlap, fixations were considered for all relevant AOIs, i.e., all fixations on the hand or hand were also counted as body fixations. Dwell times were aggregated by computing the median dwell time per dyad, condition and AOI, resulting in six values per participant for each dyad (each three AOI values for the segments with high and low IPS_mov_). To consider overall differences in fixation durations, we computed dwell times as a proportion of total duration of all fixations regardless of location, slightly deviating from the preregistered fixation durations.

We used Bayesian linear mixed models as implemented in the brms package [51], assessing our hypotheses with the brms::hypothesis() function using α = 0.05 for directed and α = 0.025 for undirected hypotheses. We used two models to test our hypotheses, one focusing on relevant predictors for the impression ratings and one for the dwell times. Both models included the population-level predictors diagnostic status of the observer (autistic or comparison), dyad type (mixed or non-autistic) and IPS_mov_ (high or low). The model assessing dwell times additionally included the population-level predictor AOI (hand, head, body). We included the dyad and participants on the group-level in both models based on the recommendations by Barr [52]. While we only included intercepts on the group-level for the model assessing ratings, we also included the slopes for IPS_mov_, dyad type and AOI as well as all interactions for the participants and the slopes for diagnostic status of the observer, IPS_mov_ and AOI as well as all interactions for dyads on the group-level. We checked the reliability and computational faithfulness of our models using prior predictive checks, simulation-based calibration and posterior predictive checks as proposed by Schad and colleagues [53] before commencing inference testing (for details see the supplementary materials, p. 22–30 and 37–46).

In addition to our hypothesis-guided confirmatory testing, we computed two exploratory analyses. First, we used a Bayesian linear mixed model using a Poisson likelihood to assess possible influences of dyad type, IPS_mov_, diagnostic status of the observer and their interactions on the number of saccades produced. Second, we used a Bayesian multiple linear regression with a Gaussian likelihood to investigate whether gaze behaviour can predict mean impressions of how pleasant the interaction was. Specifically, we included the four gaze predictors: mean dwell times on heads, hands or bodies as well as mean number of saccades. All predictors were standardised with a *z*-score normalisation. For both models, we added random intercepts for the participant and the observed dyad on the group-level.

## Results

### Impression ratings

**Fig. 3:**
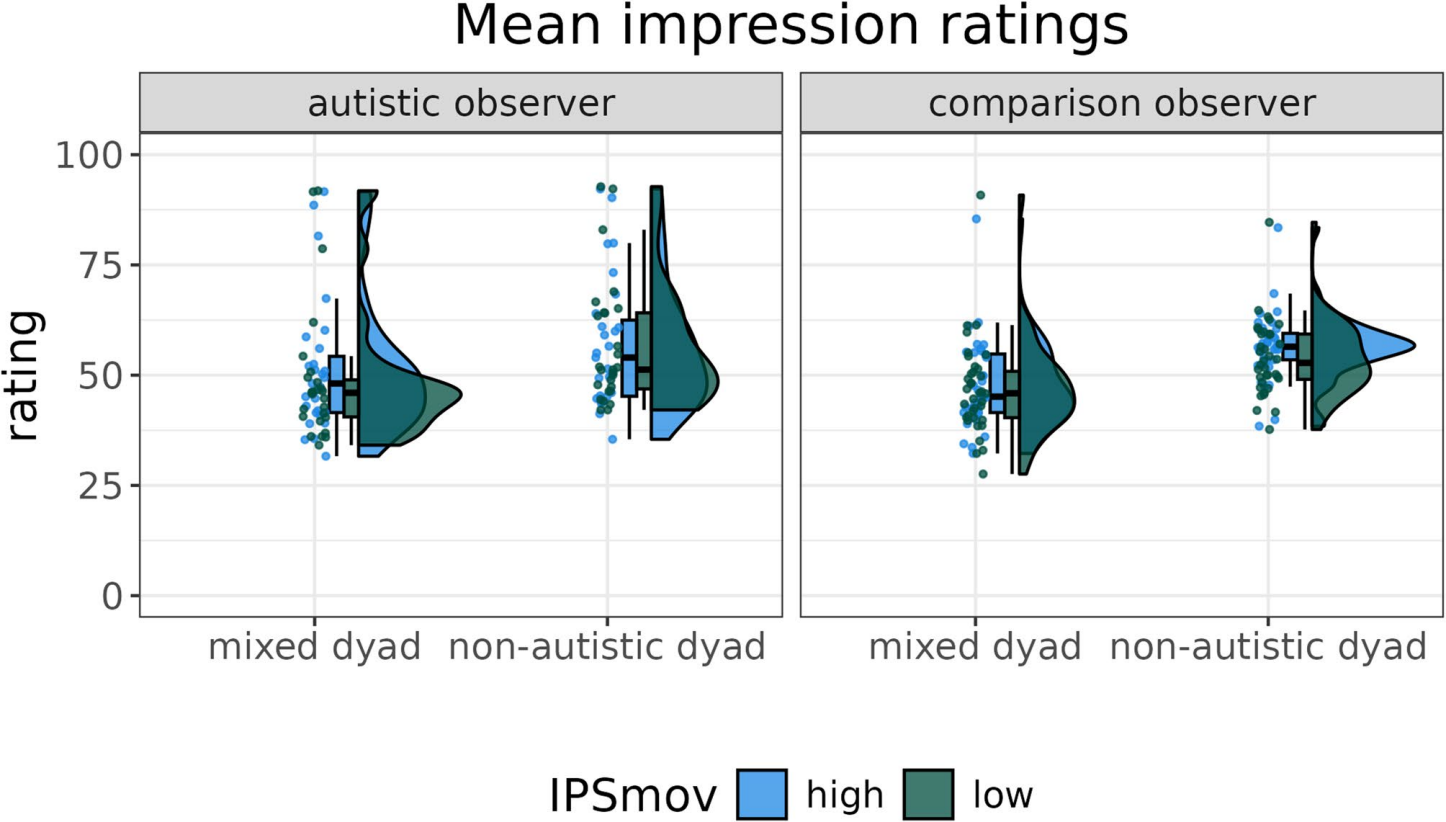
Raincloud plot visualising the mean impression ratings for each participant for each dyad type and IPS_mov_. The graph shows reduced ratings for mixed dyads as well as segments with low IPS_mov_, independent of the two groups of observers

The model investigating aggregated impression ratings revealed support for our hypotheses postulating decreased ratings for mixed dyads compared to non-autistic dyads (*estimate* = -3.87 [-6.79, -1.01], *posterior probability* = 0.984) and increased ratings for segments with high IPS_mov_ as opposed to segments with low IPS_mov_ (*estimate* = 0.83 [0.16, 1.49], *posterior probability* = 0.978). Specifically, the model predicts a mean difference of 7.7 [0.8, 14.9] between non-autistic and mixed dyads as well as a mean differences of 1.7 [0.0, 3.2] between high and low IPS_mov_ (see Fig. 3). However, there is no support for our hypotheses regarding differences between the ratings of autistic and comparison observers (*estimate* = 1.07 [-1.61, 3.78], *posterior probability* = 0.786) or the interaction of IPS_mov_ and dyad type (*estimate* = -0.06 [-0.71, 0.6], *posterior probability* = 0.555) or dyad type and diagnostic status of the observer (*estimate* = 0.24 [-0.41, 0.89], *posterior probability* = 0.739) or any other interaction (see supplementary materials, p. 31).

### Dwell times

**Fig. 4:**
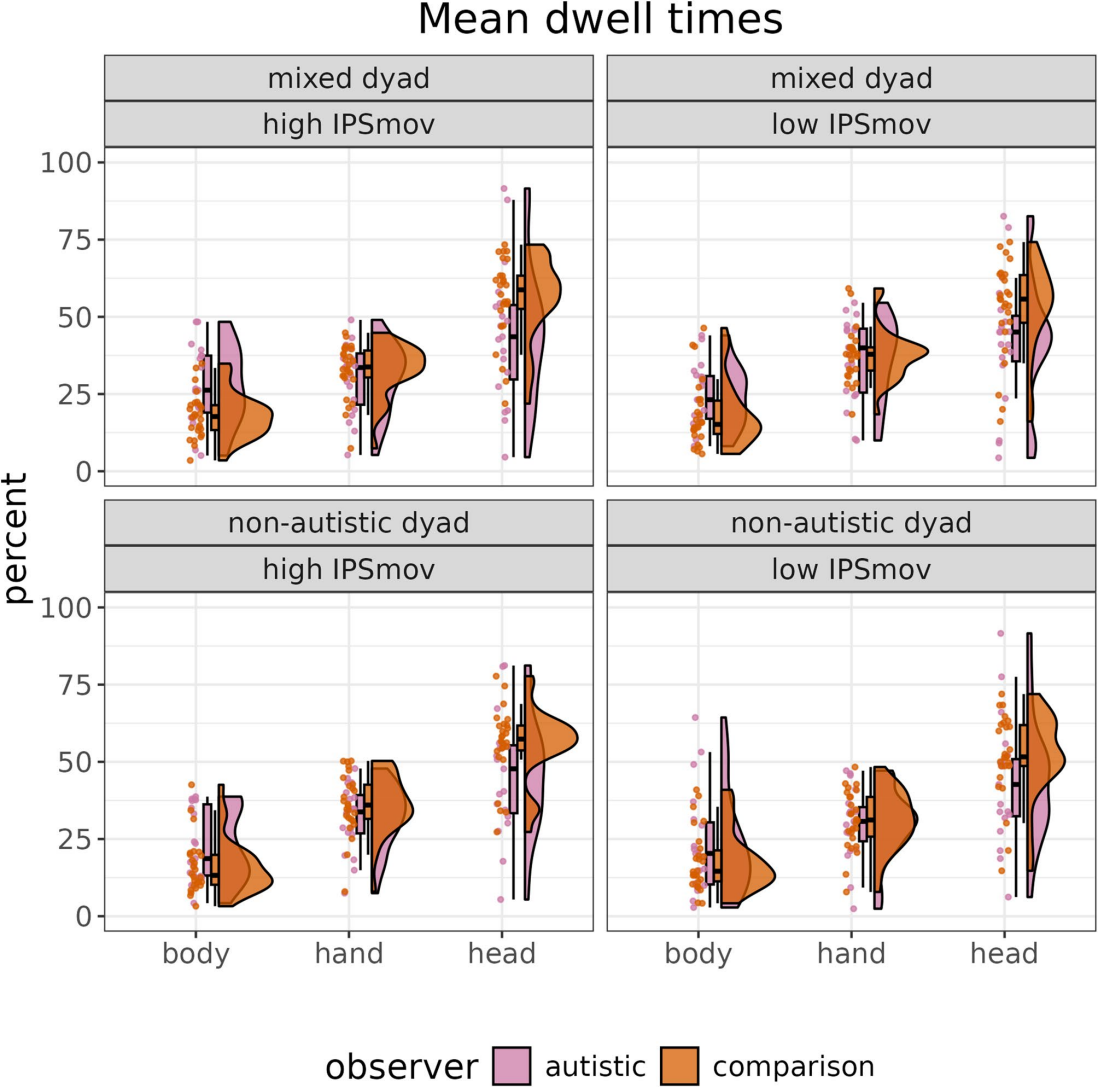
Raincloud plots visualising the mean dwell times of each participant. There is a clear pattern of differences in dwell times between autistic and non-autistic comparison observers, regardless of the dyad type and IPS_mov_. Specifically, autistic observers seem to dwell less on the head regions of the observed interaction partners

The model focusing on dwell times revealed support for our expectation of differences between autistic and comparison observers dependent on the specific AOI (*estimate* = -2.20% [-4.11, -0.31], *posterior probability* = 0.987). Further exploration shows that this is driven by decreased dwell times on the head region for autistic observers (*estimate* = 4.97% [0.53, 9.50], *posterior probability* = 0.986). Specifically, this model predicts a difference of dwell times on the heads of the interaction partners of 10.0% [1.1, 19.0] between autistic and comparison observers (see Fig. 4). There is no support for our hypotheses regarding interactions between IPS_mov_ and AOI (*estimate* = 0.13% [-1.55, 1.85], *posterior probability* = 0.570) or dyad type and AOI (*estimate* = -0.19% [-3.19, 2.87], *posterior probability* = 0.555).

### Exploratory analyses

In general, observers produced on average 19.3 +- 0.7 saccades per 10-second segment. There were no credible differences between any of the task conditions or interactions. However, autistic observers produced fewer saccades than non-autistic observers (*estimate* = 0.06 [0, 0.13], *posterior probability* = 0.98). Specifically, the model predicts that autistic observers produce on average 17.7 [15.9, 19.6] saccades, while comparison observers are predicted to produce 20.2 [18.3, 22.0] saccades on average. Predicting impression ratings with gaze behaviour revealed that more favourable impressions were associated with reduced dwell times on the hands (Scaled dwell times on hands: *estimate* = -2.27 [-3.83, -0.7]) but no other gaze behaviour (see supplementary materials, p. 81).

## Discussion

In this study, we investigated influences on impressions of observed interactions, specifically between either two non-autistic interaction partners or between one autistic and one non-autistic interaction partner. We found that the dyad type influenced the impressions formed by others, such that interactions of mixed dyads were rated less favourable than non-autistic dyads. Thus, segments of mixed dyads showing high IPS_mov_ were rated as less pleasant than non-autistic dyads showing comparably high IPS_mov_. Additionally, high IPS_mov_ elicited more favourable ratings than low IPS_mov_. While impression ratings were comparable between autistic and comparison observers, autistic observers spent less time dwelling on the head regions of the interaction partners. This difference in dwell time suggests that autistic observers used different aspects of the perceived interaction to infer a similar conclusion regarding their impression of how pleasant the interaction is, consistent with previous literature [54].

Differences in impressions of non-autistic and mixed dyads mirror previous results on impressions of autistic and non-autistic individuals, indicating that autistic people themselves [18, 20] as well as interactions of mixed dyads [28] evoke less favourable impressions. In order to minimise alternative characteristics of the interaction partners, we omitted audio to rule out observers being influenced by content or speech patterns [14, 55, 56] and only showed contours of the interaction partners in the segments. Through this procedure, we captured differences in impressions formed for mixed and non-autistic dyads based on the individual interaction partners’ movements as well as the coordination of these movements. Additionally, our segments were only “thin slices” of 10s to capture almost instantaneous impressions of the interactions [20, 57]. These segments still contained various information about both the interaction partners and the interaction, visible as significant differences between mixed and non-autistic dyads. On the level of the dyad, specific movement patterns could influence impression formation. For example, movement indicating shared laughter can lead to the impression of a more pleasant interaction. On the level of the individual interaction partner, their movement patterns or posture could indicate their feeling of comfort in the interaction which could have influenced impressions. Furthermore, if one of the interaction partners was perceived as less likeable, observers might also perceive the interaction as less pleasant. Previous studies reported more favourable impressions being formed of female compared to male children and adults, although it is still unclear how this effect interacts with less favourable impressions associated with ASD [58–60]. Most features associated with gender expression were removed by simplifying the depicted interaction partners to their contours, however, participants might still have perceived or assumed certain genders of the interaction partners. Since we do not have gender information on the interaction partners except that all dyads are same-gender, we cannot assess this based on the here-presented data. The root of these differences in impressions between mixed and non-autistic dyads should be carved out in future research, by separately manipulating features of the individual and the dyad and extracting specific movement patterns from interactions to assess their influence on impressions.

By reducing the information our observers received about interactants on motion dynamics, we were able to show that low IPS_mov_ leads to less favourable impressions of others. Our results indicate that smooth, dynamic interactions with high IPS_mov_ lead to more favourable impressions and could support Crompton and colleagues’ [28] suggestion that stable interpersonal coordination may facilitate higher rapport ratings. This effect is also in line with a study by Miles and colleagues [13] showing evidence that IPS_mov_ of two walkers influenced rapport impression regardless of whether walking scenes were presented visually or auditorily. Importantly, previous literature has shown both mixed and autistic dyads producing decreased overall IPS_mov_ [5]. However, while overall IPS_mov_ is an important characteristic of an interaction, social interactions show fluctuations of IPS_mov_ while they unfold [61]. Thus, we decided to use short segments portraying high and low IPS_mov_ for each dyad to ensure that the observed effect of increased impression ratings could be clearly attributed to high or low IPS_mov_. Since non-autistic dyads seem to produce more IPS_mov_ overall, they could benefit more from the positive effect it has on impressions formed by observers. Future studies could pair methods capturing the development of experiencing how pleasant an interaction is with methods capturing impressions by observers to assess how close experience is to observation.

This study did not reveal any differences in impressions formed by autistic and comparison observers, neither in terms of overall nor in terms of interactive effects. These comparable impressions are in line with studies on observed interactions [28] and on impression formation of individual people [18]. However, other studies suggest differences in impression formations between autistic and comparison observers [26, 27] and intentions based on impressions [18]. In the current study, IPS_mov_ had a similar effect on impressions by autistic and comparison observers. This similarity of impressions between autistic and comparison observers is in line with recent evidence showing spared neural correlates of IPS_mov_ processing based on observing segments of the same social interactions as used in this study [15]. Bierlich and colleagues also did not find any differences in naturalness impressions between autistic and comparison observers. This pattern of results could suggest that autistic and comparison observers arrive at comparable interpretations when observing social interactions showing different levels of IPS_mov_.

Less favourable impressions, both of an individual person and of interactions they are part of, can have negative implications for their everyday life by reducing the willingness of others to interact with them [19, 20]. These implications are especially important since impressions can have long lasting effects on relationship development [62]. Suggestions on how to navigate and approach these implications exist both on the side of the observer and of the person who is the target of the impression. Movement interventions based on imitation and synchronisation could potentially to some extent give autistic adults the tools to increase IPS_mov_ [63]. Concerning the observer, diagnostic labels can lead to more positive impressions, possibly indicating the capacity to adjust expectations [21, 64]. Impressions of autistic individuals by comparison observers might improve if the comparison observers have more knowledge on ASD [21]. In a recent study, autism awareness training did not lead to more favourable impressions of autistic people’s character after a short conversation between autistic and non-autistic participants. However, the autism awareness training increased socialisation intentions with the autistic people in non-autistic interaction partners; thus, mitigating the negative effect of less favourable impressions [65]. This improvement could be due to reframing an interaction as characteristic for autism instead of characteristic of a less pleasant interaction.

We did not find any differences in dwell times to specific features between mixed and non-autistic or between high or low IPS_mov_ segments. Nonetheless, despite comparable impressions, autistic and comparison observers produced slightly different gaze behaviour while watching the interactions. Dwell times on the head of interactants were reduced in autistic compared to comparison observers. Reduced dwell times or speed to fixation in response to social stimuli like heads, faces or eyes in ASD has been shown throughout the literature [30, 66, 67]. Additionally, autistic observers produced fewer saccades compared to comparison observers, possibly indicating that they focused on fewer aspects of the interaction while comparison observers more often moved their eyes from one detail to the next. Considering our results, there is now converging evidence of differential social attention in autism. Importantly, our findings extend this feature to dynamic social interaction stimuli focusing on movement patterns alone and thereby show that altered social attention is found across a wide array of social stimuli in autism and particularly also in stimuli where no facial features are visible.

However, increased dwell times on hands predicted less favourable impressions. This effect could be associated with differences in movement patterns between interactions perceived as more or less pleasant. For example, less pleasant interactions might be associated with more intense hand gesturing, resulting in more dwell times on the hands. In the current study, we did not extract gestures or specific movement patterns from the segments and, thus, cannot assess this possibility. Alternatively, focusing on the hands might lead to less favourable impressions regardless of the interaction. To disentangle these mechanisms, future research could manipulate attention to different aspects of social interactions to assess the influence of attention patterns on impressions.

## Limitations

The exact impact of the effects of IPS_mov_ and dyad type on impressions in real-life cannot be estimated on the basis of this study. Real-life impression formation depends on a wide variety of information, including facial expressiveness, gaze behaviour and conversational content. In this study, we explicitly focused on manipulating two possible influences separately: IPS_mov_ and dyad type. Segments showing high IPS_mov_ were estimated to be rated 1.7 points more favourable on our scale from 0 to 100, with 95% of the estimated values between 0.0 and 3.2 points. This effect is potentially negligible when observing interactions in real-life. However, this effect only captures IPS_mov_, while in reality we observe multiple channels in which interaction partners can be in sync or not. Small effects on each channel might add up to significantly less favourable impressions, making IPS a possible key factor in forming impressions of interactions. Future research should assess this possibility by measuring the additive influence of different channels of IPS on impressions. The effect of dyad type was estimated between 0.8 and 14.9 points with an average of 7.7 points difference between mixed dyads consisting of one autistic and one non-autistic person and dyads consisting of two non-autistic people. This effect is almost five times as large as the effect of IPS_mov_. Yet, it is still unclear what differences between these dyad types cause the influence of dyad type on impressions. In this study, observers only saw 10s segments of people’s outlines, so they received no auditory information regarding the interaction and only limited information on appearances. Future studies should dissect the exact movement patterns such as gestures to further carve out which behavioural differences cause less favourable impressions for these mixed dyads. Furthermore, it is paramount to extend the research to a wider range of mixed and non-autistic dyads assessing how the findings generalise as well as to interactions of two autistic people further carving out how autism interacts with impressions. Last, the average intelligence estimate in the sample analysed here was quite high and we excluded non-verbal autistic adults; thus, generalisability is limited to a subset of adults with ASD without intelligence disability.

## Conclusions

This study illuminates predictors of impression formation of dyadic interactions, particularly those involving autistic adults. Results highlight the importance of motion dynamics in shaping these impressions, with smoother, more coordinated interactions as measured by IPS_mov_ being perceived as more favourable, specifically more pleasant. In addition to this effect of IPS_mov_ on impressions, interactions between autistic and non-autistic adults were generally rated as less pleasant than interactions between two non-autistic adults. While both autistic and comparison observers formed similar impressions, differences in their gaze patterns could suggest distinct strategies for processing social information. While the effects of IPS_mov_ was small in this study, interaction partners can be in sync in many ways, not only in their motion dynamics. Possible additive effects could underscore the importance of IPS in fostering positive social interactions. Future research should examinate other predictors of less favourable impressions associated with autism.

## Electronic supplementary material

Below is the link to the electronic supplementary material.

Supplementary Material 1

## Data Availability

The dataset supporting the conclusions of this article is available in the GitHub repository, 10.5281/zenodo.15241671 or https://github.com/IreneSophia/PESI.

## Abbreviations

AOI: Areas of Interest
ASD: Autism spectrum disorder
BDI: Beck Depression Inventory
CFT: Culture Fair Intelligence Test
COMP: Comparison group of non-autistic observers
IPS: Interpersonal synchrony
IPS_mov_: Interpersonal synchrony of movement
MEA: Motion Energy Analysis
RADS-R: Ritvo Autism Diagnostic Scale– Revised
STAI: State and Trait Anxiety Inventory
UI: Intolerance for Uncertainty
