# Supplementary material for: The influence of interpersonal synchrony and autism on impressions of dyadic interactions: a preregistered study
Source: Mol Autism. 2025 Jun 12;16:34. doi: 10.1186/s13229-025-00668-y (PMC12164215; doi:10.1186/s13229-025-00668-y)
Supplement: Supplementary file 1 — Supplementary Material 1 [file 13229_2025_668_MOESM1_ESM.pdf]

PESI analysis with brms

I. S. Plank

2025-04-16

Introduction

The PESI project focuses on "Perception of nonverbal social interaction in people with and without autism". Specifically, autistic and non-autistic comparison observers watched 64 silent videos of dyadic interactions between two non-autistic or one autistic and one non-autistic adult. The visual angle of the interaction was approximately 26.05 degrees wide and 18 degrees high, depending on the exact frame. We created circular areas of interest (AOIs) for dwell times on the heads and the hands. Their visual angle were 5.85 and 3.91 degrees, respectively.

All observers were adults. These videos were 10 seconds of longer interactions and either portrayed moments of high or low interpersonal synchrony (IPS) of movement. The videos only showed outlines of the dyad to direct the focus on the movement. During the video presentation, observers' gaze is tracked and after each video they are asked to rate how comfortable they imagine this interaction to be.

Instructions

Participants were neither made aware that some of the videos showed mixed and some showed non-autistic dyads nor that we were interested in interpersonal synchrony. They were given the following instructions:

German original: "In dieser Aufgabe sehen Sie Videos von Interaktionen zwischen jeweils zwei Menschen. Alle Menschen kommen dabei öfters vor. Sie sehen nur die Umrisse der Menschen und die Videos sind stumm. Nach jedem Video werden Sie gebeten, auf einer Skala anzugeben, wie angenehm Sie die Interaktion fanden. Mit der Taste 4 können Sie den Regler nach links verschieben, mit der Taste 8 nach rechts und mit der Taste 8 bestätigen Sie Ihre Auswahl. Bitte wählen Sie zügig aus und bestätigen Sie jede Auswahl. Sie haben für jede Auswahl 8 Sekunden Zeit. Drücken Sie jetzt die Taste 8, um die Übung zu starten!"

English translation: "In this task, you will see videos of interactions between two people. You will see each person multiple times, you will only see the outlines of the videos and the videos are silent. After each video, you will be asked to indicate on a scale how pleasant you found the interaction. Use the key 4 on the number block to move the slider to the left, the key 6 to move it to the right and the key 8 to confirm your selection. Please select quickly how pleasant you found the interaction and then confirm. You have 8 seconds for each rating. Press the key 8 to start the task!"

Hypotheses

We preregistered the following hypotheses:

1. Ratings
   (a) Dyad type: Social interactions of no-diagnosis non-autistic dyads are rated more positively than mixed-diagnosis dyads consisting of one autistic and one non-autistic interaction partner.
   (b) Synchrony: Social interactions with high interpersonal synchrony of motion energy are rated more positively than social interactions with low interpersonal synchrony of motion energy.

(c) Diagnostic status: Ratings of social interactions differ between autistic and non-autistic comparison observers.

(d) Synchrony x dyad type: The effect of interpersonal motion synchrony on ratings is decreased for mixed-diagnosis dyads compared to no-diagnosis dyads.

(e) Dyad type x diagnostic status: The effect of dyad type on ratings is decreased in autistic compared to comparison observers.

2. Fixation duration in areas of interest (AOI)

   (a) Dyad type x AOI: Fixation durations for each area of interest differ between clips of no-diagnosis and mixed-diagnosis dyads.

   (b) Synchrony x AOI: Fixation durations for each area of interest differ between high and low synchrony clips.

   (c) Diagnostic status x AOI: Fixation durations for each area of interest differ between autistic and comparison observers.

Some general settings

```
# number of simulations
nsim = 500

# set number of iterations and warmup for models
iter = 4500
warm = 1500

# set the seed
set.seed(2468)
```

Package versions

```
## [1] "R version 4.5.0 (2025-04-11)"

## [1] "knitr version 1.50"
## [1] "ggplot2 version 3.5.2"
## [1] "brms version 2.22.0"
## [1] "designr version 0.1.13"
## [1] "bridgesampling version 1.1.2"
## [1] "tidyverse version 2.0.0"
## [1] "ggpubr version 0.6.0"
## [1] "ggrain version 0.0.4"
## [1] "bayesplot version 1.12.0"
## [1] "SBC version 0.3.0.9000"
## [1] "rstatix version 0.7.2"
## [1] "readODS version 2.3.2"
## [1] "vtable version 1.4.8"
## [1] "BayesFactor version 0.9.12.4.7"
## [1] "bayestestR version 0.15.2"
```

Stimulus selection

PESI stimulus selection

Irene S. Plank

15/02/2023

Introduction

Script to read in MEA data, preprocess it and compute CCF based synchrony for each dyad. The script needs the MEA files separated by location (Munich or Cologne), group (ASD or TD) and fps in separate folders. The folder names are:

- "./MEA txt files_5min_t15_Cologne/TD-ASD/24fps"
- "./MEA txt files_5min_t15_Cologne/TD-ASD/24fps"
- "./MEA txt files_5min_t15_Cologne/TD-ASD/25fps"
- "./MEA txt files_5min_t15_Cologne/TD-ASD/25fps"

The script produces a csv file with the selected videos.

Motion Energy Analysis

```
# initialisation of all settings
st  = 1          # start times (seconds)
sz  = 10         # window size for ccf (seconds)
inc = sz
lg  = 3          # lag for ccf (seconds)

# 25fps ------------------------------------------------------------------

fps = 25

## COLOGNE SAMPLE

# read in total ROI data of ASD dyads
mea_ASD_head_C = readMEA("./MEA txt files_5min_t15_Cologne/TD-ASD/25fps",
                           sampRate=fps,
                           skip=st*fps,
                           s1Col = c(1), s2Col = c(3),
                           s1Name = "L_head", s2Name = "R_head",
                           header = F,
                           idOrder = c("id","group"),
                           idSep = "_",
                           sep = "")
```

```
##
## STEP 1 | Reading 5 dyads
## ..................................................|100%
## ..................................................|Done ;)
##
## STEP 2 | Formatting data frames:
## ..................................................|100%
## ..................................................|Done ;)
```

```
## Warning: 0.01% of the data was higher than 10 standard deviations in dyad: D22g,
## session: 01, group:25fps. Check the raw data!
```

```
## Warning: 0.01% of the data was higher than 10 standard deviations in dyad: D24g,
## session: 01, group:25fps. Check the raw data!
```

```
## Warning: 0.03% of the data was higher than 10 standard deviations in dyad: D25g,
## session: 01, group:25fps. Check the raw data!
```

```
## Warning: 0.09% of the data was higher than 10 standard deviations in dyad: D27g,
## session: 01, group:25fps. Check the raw data!
```

```
## Warning: 0.01% of the data was higher than 10 standard deviations in dyad: D28g,
## session: 01, group:25fps. Check the raw data!
```

```
##
## STEP 3 | ReadMEA report
##         Filename id_dyad session group duration_hh.mm.ss L_head_% R_head_%
## 1 D22g_25fps.txt    D22g      01 25fps          00:05:00     51.7     47.3
## 2 D24g_25fps.txt    D24g      01 25fps          00:05:00     56.1     48.4
## 3 D25g_25fps.txt    D25g      01 25fps          00:05:00     36.7     39.1
## 4 D27g_25fps.txt    D27g      01 25fps          00:05:00     52.4     54.1
## 5 D28g_25fps.txt    D28g      01 25fps          00:05:00     28.7     83.5
```

```
mea_ASD_head_C = setGroup(mea_ASD_head_C, "ASD")

## read in total ROI data of TD dyads
mea_TD_head_C = readMEA("./MEA txt files_5min_t15_Cologne/TD-TD/25fps",
                          sampRate=fps,
                          skip=st*fps,
                          s1Col = c(1), s2Col = c(3),
                          s1Name = "L_head", s2Name = "R_head",
                          header = F,
                          idOrder = c("id","group"),
                          idSep = "_",
                          sep = "")
```

```
##
## STEP 1 | Reading 4 dyads
## ..................................................|100%
## ..................................................|Done ;)
##
## STEP 2 | Formatting data frames:
## ..................................................|100%
## ..................................................|Done ;)
```

```
## Warning: 0.07% of the data was higher than 10 standard deviations in dyad: D19h,
## session: 01, group:25fps. Check the raw data!
```

```
##
## STEP 3 | ReadMEA report
##         Filename id_dyad session group duration_hh.mm.ss L_head_% R_head_%
## 1 D18h_25fps.txt    D18h      01 25fps          00:04:58     72.3     61.8
## 2 D19h_25fps.txt    D19h      01 25fps          00:04:58     62.8     72.6
## 3 D23h_25fps.txt    D23h      01 25fps          00:05:00     28.4     40.0
## 4 D26h_25fps.txt    D26h      01 25fps          00:05:00     60.1     64.8
```

```
mea_TD_head_C = setGroup(mea_TD_head_C, "TD")

## Combine into a single object
mea_all_head = c(mea_ASD_head_C, mea_TD_head_C)

## Preprocessing and calculations
mea_all_head = MEAscale(mea_all_head)
```

```
##
## Rescaling data:
## ..................................................|100%
## ..................................................|Done ;)
```

```
## Run CCF analysis
mea_all_head = MEAccf(mea_all_head, lagSec=lg, winSec=sz, incSec=inc, r2Z=T, ABS=T)
```

```
##
## Computing CCF:
## ..................................................|100%
## ..................................................|Done ;)
```

```
## put information into the dataframe
df.head = createDFsync(mea_all_head, sep = "_", desc = c("group", "dyad", "session"), fps)
```

```
## ── Attaching core tidyverse packages ──────────────────────── tidyverse 2.0.0 ──
## ✔ dplyr     1.0.10     ✔ purrr     1.0.1
## ✔ forcats   0.5.2      ✔ readr     2.1.2
## ✔ ggplot2   3.4.0      ✔ tibble    3.1.8
## ✔ lubridate 1.8.0      ✔ tidyr     1.2.1
## ── Conflicts ────────────────────────────────────────── tidyverse_conflicts() ──
## ✖ tidyr::expand() masks Matrix::expand()
## ✖ dplyr::filter() masks stats::filter()
## ✖ dplyr::id()     masks rMEA::id()
## ✖ dplyr::lag()    masks stats::lag()
## ✖ tidyr::pack()   masks Matrix::pack()
## ✖ tidyr::unpack() masks Matrix::unpack()
## ℹ Use the ]8;;http://conflicted.r-lib.org/conflicted package]8;; to force all conflicts to become errors
```

```
df.head = df.head %>%
  rename("context" = "group") %>%
  mutate(
    context = as.factor(context),
    context = recode_factor(context,
      'ASD' = "heterogeneous",
      'TD'  = "homogeneous"
    )
  ) %>% select(-session)

# create total motion variables for each dyad
df.head$mot = rowMeans(df.head[,c("s1mot","s2mot")])
df = df.head

# filter out windows with extreme motion
df.head = df %>% ungroup() %>%
  mutate(
    upper = quantile(mot, probs=c(0.75), na.rm = T),
    lower = quantile(mot, probs=c(0.25), na.rm = T)
  ) %>%
  filter(lower <= mot & mot <= upper) %>%
  drop_na() %>% filter(
    dyad != "D28g"
  )
```

Choose videos

```
df.head_agg = df.head %>% group_by(context, dyad) %>%
  summarise(
    n_win = n()
  )
```

```
## `summarise()` has grouped output by 'context'. You can override using the
## `.groups` argument.
```

```
# select the n videos of each dyad with the highest and the n with the lowest synchrony values (peak)
n = 4 # number of windows per condition per video
vid_sel_high = df.head %>%
  arrange(desc(peak)) %>%
  group_by(dyad) %>%
  slice(1:n) %>%
  mutate(
    sync = "high"
  )

vid_sel_low = df.head %>%
  arrange(peak) %>%
  group_by(dyad) %>%
  slice(1:n) %>%
  mutate(
    sync = "low"
  )

# add frame for start and end

vid_sel = rbind(vid_sel_low, vid_sel_high) %>%
  mutate(
    frame_sta = as.numeric(substr(winst,5,5))*60*fps + as.numeric(substr(winst,7,8))*fps,
    frame_end = as.numeric(substr(winen,5,5))*60*fps + as.numeric(substr(winen,7,8))*fps
  )
```

General info

We planned to determine the group-level effect subjects following Barr (2013). For each model, experiment specific priors were set based on previous literature or the task (see comments in the code).

We perform prior predictive checks as proposed in Schad, Betancourt and Vasishth (2020) using the SBC package. To do so, we create simulated datasets where parameters are simulated from the priors. These parameters are used to create one fake dataset. Both the true underlying parameters and the simulated values are saved.

Then, we create graphs showing the prior predictive distribution of the simulated discrimination threshold to check whether our priors fit our general expectations about the data. Next, we perform checks of computational faithfulness and model sensitivity as proposed by Schad, Betancourt and Vasishth (2020) and implemented in the SBC package. We create models for each of the simulated datasets. Last, we calculate performance metrics for each of these models, focusing on the population-level parameters.

Preparation and group comparisons

First, we load the data and combine it with demographic information including the diagnostic status of the subjects. Then, all predictors are set to sum contrasts. We have a look at the demographics describing our two diagnostic groups: autistic adults and adults without any neurological and psychiatric diagnoses.

Since this is sensitive data, we load the anonymised version of the processed data at this point but also leave the code we used to create it.

```
# check if the data file exists, if yes load it:
if (!file.exists("PESI_data.RData")) {

  # set file paths
  fl.path = '/media/emba/backup/PESI'
  dt.path = paste(fl.path, 'BVET', sep = "/")

  # read in list of participants
  df.inc = read_ods(file.path(fl.path, "PESI_inc.ods"), range = "A1:K100") %>%
    select(subID, include_BV, include_ET)

  # create an anonymisation key
  df.inc = df.inc %>%
    mutate(
      PID   = subID,
      subID = as.numeric(as.factor(subID))
    )
  df.recode = df.inc %>% select(PID, subID) %>% distinct()
  recode = as.character(df.recode$subID)
  names(recode) = df.recode$PID

  # filter out pilots and cancelled testings
  df.inc = df.inc %>%
    filter(include_BV == 1)

  # load the relevant data in long format
  df.beh = list.files(path = dt.path, pattern = "PESI-BV", full.names = T) %>%
    map_df(~read_delim(., show_col_types = F, delim = ",",
                       col_types = "cddcccddddd")) %>%
    select(-dyad, -sync) %>%
    filter(subID %in% df.inc$PID) %>%
    group_by(subID) %>%
```

```r
  mutate(
    no_trials = n()
  ) %>%
  # filter out participants where not the full task was recorded > no participants
  filter(no_trials == 64)

# load demographic information to get diagnostic status
df.sub = read_csv(file.path(dt.path, "PESI_centraXX.csv"), show_col_types = F) %>%
  mutate(
    diagnosis = recode(diagnosis, "CTR" = "COMP")
  )

# load the stimulus description file
df.stm = read_csv(paste(fl.path, "PESI_videosel-full_230404.csv",
                        sep = "/"), show_col_types = F) %>%
  mutate(
    video = sprintf("PESI_%s_%08d", substr(dyad,1,3), frame_sta),
    dyad.type = case_match(context,
                           "homogeneous" ~ "non-autistic",
                           "heterogeneous" ~ "mixed")
  ) %>%
  select(video, dyad, sync, dyad.type, mot, peak, s1peak, s2peak)

# merge together
df = merge(df.beh, df.stm, all.x = T, by = "video") %>%
  mutate(
    # only use trials with confirmed rating and correct video duration
    use = if_else(confirmed == 1 & abs(dur-10) < 0.1, 1,0),
    rating.confirmed = if_else(use == 1, rating, NA)
       ) %>%
  arrange(subID, trl)

# merge with group information
df = merge(df.sub %>% select(subID, diagnosis), df, all.y = T) %>%
  mutate_if(is.character, as.factor)

# check how many participants per group need to be excluded due to low trial numbers
df.exc = df %>%
  group_by(subID, diagnosis) %>%
  summarise(
    total = sum(use)/64
  ) %>%
  filter(total <= 2/3) %>%
  group_by(diagnosis) %>%
  summarise(
    n = n()
  )

# exclude participants with more than 33% of trials missing
df = df %>%
  group_by(subID) %>%
  mutate(
    total = sum(use)/64
```

```r
  ) %>%
  filter(total > 2/3)

# get a list of the subIDs
subIDs = unique(df$subID)

# anonymise the data
df$subID = str_replace_all(df$subID, recode)

# load preprocessed eye tracking data and rename variables
df.fix = readRDS(file.path(dt.path, "PESI-ET_fix.rds")) %>%
  rename(
    "trl" = "on_trialNo", "video" = "on_trialVid"
  )
df.sac.agg = readRDS(file.path(dt.path, "PESI-ET_sac.rds")) %>%
  rename(
    "trl" = "on_trialNo", "video" = "on_trialVid"
  )

# add information on the videos to the eye tracking data
df.fix = merge(df.fix, df.stm, all.x = T) %>%
  # add diagnostic group
  merge(., df.sub %>% select(subID, diagnosis), all.x = T) %>%
  mutate(across(where(is.character), as.factor))
df.sac.agg = merge(df.sac.agg, df.stm, all.x = T) %>%
  # add diagnostic group
  merge(., df.sub %>% select(subID, diagnosis), all.x = T) %>%
  mutate(across(where(is.character), as.factor))

# add subjects to the subIDs
subIDs.et = unique(c(df.sac.agg$subID, df.fix$subID))

# anonymise ET data in the same way as the behavioural data
df.fix$subID     = str_replace_all(df.fix$subID, recode)
df.sac.agg$subID = str_replace_all(df.sac.agg$subID, recode)

# remove participants not included in the behavioural or the eye tracking
# analysis from the subjects data frame
df.sub.et = df.sub %>%
  filter(subID %in% subIDs.et)
df.sub = df.sub %>%
  filter(subID %in% subIDs)

# get numbers of participants included in ET per group and gender identity
df.incET = df.sub.et %>%
  group_by(diagnosis, gender) %>%
  summarise(
    n = n()
  )

# get an overview over the diagnosis of the autistic group in behavioural data
df.asd = df.sub %>%
  filter(diagnosis == "ASD") %>%
```

```r
  group_by(ASD.icd10) %>%
  count()

# print gender frequencies and compare them across groups
tb.gen  = xtabs(~ gender + diagnosis, data = df.sub)
ct.beh  = contingencyTableBF(tb.gen, sampleType = "indepMulti", fixedMargin = "cols")
tb.gen  = xtabs(~ gender + diagnosis, data = df.sub.et)
ct.et   = contingencyTableBF(tb.gen, sampleType = "indepMulti", fixedMargin = "cols")

# check which outcomes of interest are normally distributed
df.sht = df.sub %>%
  group_by(diagnosis) %>%
  shapiro_test(age, CFT_iq, BDI_total, STAITT_total, RAADS_total, ISH_total,
               UI_total, sw_gz, sw_kl) %>%
  arrange(variable) %>%
  mutate(
    sig = if_else(p < 0.05, "*", "")
  )
df.sht.et = df.sub.et %>%
  group_by(diagnosis) %>%
  shapiro_test(age, CFT_iq, BDI_total, STAITT_total, RAADS_total, ISH_total,
               UI_total, sw_gz, sw_kl) %>%
  arrange(variable) %>%
  mutate(
    sig = if_else(p < 0.05, "*", "")
  )

# some of the measures are not normally distributed;
# therefore, we compute ranks for these outcomes
df.sub = df.sub %>%
  mutate(
    rBDI   = rank(BDI_total),
    rISH   = rank(ISH_total),
    rRAADS = rank(RAADS_total),
    rUI    = rank(UI_total),
    rage   = rank(age),
    rsw_kl = rank(sw_kl),
    diagnosis = as.factor(diagnosis)
  )
df.sub.et = df.sub.et %>%
  mutate(
    rBDI   = rank(BDI_total),
    rISH   = rank(ISH_total),
    rRAADS = rank(RAADS_total),
    rUI    = rank(UI_total),
    diagnosis = as.factor(diagnosis)
  )

# now we can compute our ttests
ostt.age       = ttestBF(formula = rage        ~ diagnosis, data = df.sub)
ostt.iq        = ttestBF(formula = CFT_iq      ~ diagnosis, data = df.sub)
ostt.kl        = ttestBF(formula = sw_gz       ~ diagnosis, data = df.sub)
ostt.gz        = ttestBF(formula = rsw_kl      ~ diagnosis, data = df.sub)
```

```r
ostt.STAITT    = ttestBF(formula = STAITT_total ~ diagnosis, data = df.sub)
ostt.BDI       = ttestBF(formula = rBDI         ~ diagnosis, data = df.sub)
ostt.ISH       = ttestBF(formula = rISH         ~ diagnosis, data = df.sub)
ostt.RAADS     = ttestBF(formula = rRAADS       ~ diagnosis, data = df.sub)
ostt.UI        = ttestBF(formula = rUI          ~ diagnosis, data = df.sub)
ostt.age.et    = ttestBF(formula = age          ~ diagnosis, data = df.sub.et)
ostt.iq.et     = ttestBF(formula = CFT_iq       ~ diagnosis, data = df.sub.et)
ostt.kl.et     = ttestBF(formula = sw_gz        ~ diagnosis, data = df.sub.et)
ostt.gz.et     = ttestBF(formula = sw_kl        ~ diagnosis, data = df.sub.et)
ostt.STAITT.et = ttestBF(formula = STAITT_total ~ diagnosis, data = df.sub.et)
ostt.BDI.et    = ttestBF(formula = rBDI         ~ diagnosis, data = df.sub.et)
ostt.ISH.et    = ttestBF(formula = rISH         ~ diagnosis, data = df.sub.et)
ostt.RAADS.et  = ttestBF(formula = rRAADS       ~ diagnosis, data = df.sub.et)
ostt.UI.et     = ttestBF(formula = rUI          ~ diagnosis, data = df.sub.et)

# ...and put everything in a new dataframe for printing
measurement  = "Age"
ASD.beh      = sprintf("%.2f (±%.2f) [%.0f - %.0f]",
                   mean(df.sub[df.sub$diagnosis == "ASD",]$age),
                   sd(df.sub[df.sub$diagnosis == "ASD",]$age),
                   min(df.sub[df.sub$diagnosis == "ASD",]$age),
                   max(df.sub[df.sub$diagnosis == "ASD",]$age))
COMP.beh      = sprintf("%.2f (±%.2f) [%.0f - %.0f]",
                    mean(df.sub[df.sub$diagnosis == "COMP",]$age),
                    sd(df.sub[df.sub$diagnosis == "COMP",]$age),
                    min(df.sub[df.sub$diagnosis == "COMP",]$age),
                    max(df.sub[df.sub$diagnosis == "COMP",]$age))
logBF10.beh  = sprintf("%.3f", ostt.age@bayesFactor[["bf"]])
ASD.et       = sprintf("%.2f (±%.2f) [%.0f - %.0f]",
                   mean(df.sub.et[df.sub.et$diagnosis == "ASD",]$age),
                   sd(df.sub.et[df.sub.et$diagnosis == "ASD",]$age),
                   min(df.sub.et[df.sub.et$diagnosis == "ASD",]$age),
                   max(df.sub.et[df.sub.et$diagnosis == "ASD",]$age))
COMP.et       = sprintf("%.2f (±%.2f) [%.0f - %.0f]",
                    mean(df.sub.et[df.sub.et$diagnosis == "COMP",]$age),
                    sd(df.sub.et[df.sub.et$diagnosis == "COMP",]$age),
                    min(df.sub.et[df.sub.et$diagnosis == "COMP",]$age),
                    max(df.sub.et[df.sub.et$diagnosis == "COMP",]$age))
logBF10.et    = sprintf("%.3f", ostt.age.et@bayesFactor[["bf"]])
df.table = data.frame(measurement, ASD.beh, COMP.beh, logBF10.beh,
                      ASD.et, COMP.et, logBF10.et)
df.table = rbind(df.table,
                 c(
                   "BDI",
                   sprintf("%.2f (±%.2f) [%.0f - %.0f]",
                           mean(df.sub[df.sub$diagnosis == "ASD",]$BDI_total),
                           sd(df.sub[df.sub$diagnosis == "ASD",]$BDI_total),
                           min(df.sub[df.sub$diagnosis == "ASD",]$BDI_total),
                           max(df.sub[df.sub$diagnosis == "ASD",]$BDI_total)),
                   sprintf("%.2f (±%.2f) [%.0f - %.0f]",
                           mean(df.sub[df.sub$diagnosis == "COMP",]$BDI_total),
                           sd(df.sub[df.sub$diagnosis == "COMP",]$BDI_total),
                           min(df.sub[df.sub$diagnosis == "COMP",]$BDI_total),
```

```
                max(df.sub[df.sub$diagnosis == "COMP",]$BDI_total)),
        sprintf("%.3f", ostt.BDI@bayesFactor[["bf"]]),
        sprintf("%.2f (±%.2f) [%.0f - %.0f]",
                mean(df.sub.et[df.sub.et$diagnosis == "ASD",]$BDI_total),
                sd(df.sub.et[df.sub.et$diagnosis == "ASD",]$BDI_total),
                min(df.sub.et[df.sub.et$diagnosis == "ASD",]$BDI_total),
                max(df.sub.et[df.sub.et$diagnosis == "ASD",]$BDI_total)),
        sprintf("%.2f (±%.2f) [%.0f - %.0f]",
                mean(df.sub.et[df.sub.et$diagnosis == "COMP",]$BDI_total),
                sd(df.sub.et[df.sub.et$diagnosis == "COMP",]$BDI_total),
                min(df.sub.et[df.sub.et$diagnosis == "COMP",]$BDI_total),
                max(df.sub.et[df.sub.et$diagnosis == "COMP",]$BDI_total)),
        sprintf("%.3f", ostt.BDI.et@bayesFactor[["bf"]])

      ),
      c(
        "Gender (diverse/agender/non-binary - female - male)",
        sprintf("%d - %d - %d",
                nrow(df.sub[df.sub$diagnosis == "ASD" &
                              df.sub$gender == "dan",]),
                nrow(df.sub[df.sub$diagnosis == "ASD" &
                              df.sub$gender == "fem",]),
                nrow(df.sub[df.sub$diagnosis == "ASD" &
                              df.sub$gender == "mal",])),
        sprintf("%d - %d - %d",
                nrow(df.sub[df.sub$diagnosis == "COMP" &
                              df.sub$gender == "dan",]),
                nrow(df.sub[df.sub$diagnosis == "COMP" &
                              df.sub$gender == "fem",]),
                nrow(df.sub[df.sub$diagnosis == "COMP" &
                              df.sub$gender == "mal",])),
        sprintf("%.3f", ct.beh@bayesFactor[["bf"]]),
        sprintf("%d - %d - %d",
                nrow(df.sub.et[df.sub.et$diagnosis == "ASD" &
                              df.sub.et$gender == "dan",]),
                nrow(df.sub.et[df.sub.et$diagnosis == "ASD" &
                              df.sub.et$gender == "fem",]),
                nrow(df.sub.et[df.sub.et$diagnosis == "ASD" &
                              df.sub.et$gender == "mal",])),
        sprintf("%d - %d - %d",
                nrow(df.sub.et[df.sub.et$diagnosis == "COMP" &
                              df.sub.et$gender == "dan",]),
                nrow(df.sub.et[df.sub.et$diagnosis == "COMP" &
                              df.sub.et$gender == "fem",]),
                nrow(df.sub.et[df.sub.et$diagnosis == "COMP" &
                              df.sub.et$gender == "mal",])),
        sprintf("%.3f", ct.et@bayesFactor[["bf"]])
      ),
      c(
        "IQ",
        sprintf("%.2f (±%.2f) [%.0f - %.0f]",
                mean(df.sub[df.sub$diagnosis == "ASD",]$CFT_iq),
                sd(df.sub[df.sub$diagnosis == "ASD",]$CFT_iq),
```

```r
            min(df.sub[df.sub$diagnosis == "ASD",]$CFT_iq),
            max(df.sub[df.sub$diagnosis == "ASD",]$CFT_iq)),
    sprintf("%.2f (±%.2f) [%.0f - %.0f]",
            mean(df.sub[df.sub$diagnosis == "COMP",]$CFT_iq),
            sd(df.sub[df.sub$diagnosis == "COMP",]$CFT_iq),
            min(df.sub[df.sub$diagnosis == "COMP",]$CFT_iq),
            max(df.sub[df.sub$diagnosis == "COMP",]$CFT_iq)),
    sprintf("%.3f", ostt.iq@bayesFactor[["bf"]]),
    sprintf("%.2f (±%.2f) [%.0f - %.0f]",
            mean(df.sub.et[df.sub.et$diagnosis == "ASD",]$CFT_iq),
            sd(df.sub.et[df.sub.et$diagnosis == "ASD",]$CFT_iq),
            min(df.sub.et[df.sub.et$diagnosis == "ASD",]$CFT_iq),
            max(df.sub.et[df.sub.et$diagnosis == "ASD",]$CFT_iq)),
    sprintf("%.2f (±%.2f) [%.0f - %.0f]",
            mean(df.sub.et[df.sub.et$diagnosis == "COMP",]$CFT_iq),
            sd(df.sub.et[df.sub.et$diagnosis == "COMP",]$CFT_iq),
            min(df.sub.et[df.sub.et$diagnosis == "COMP",]$CFT_iq),
            max(df.sub.et[df.sub.et$diagnosis == "COMP",]$CFT_iq)),
    sprintf("%.3f", ostt.iq.et@bayesFactor[["bf"]])
  ),
  c(
    "RADS-R",
    sprintf("%.2f (±%.2f) [%.0f - %.0f]",
            mean(df.sub[df.sub$diagnosis == "ASD",]$RAADS_total),
            sd(df.sub[df.sub$diagnosis == "ASD",]$RAADS_total),
            min(df.sub[df.sub$diagnosis == "ASD",]$RAADS_total),
            max(df.sub[df.sub$diagnosis == "ASD",]$RAADS_total)),
    sprintf("%.2f (±%.2f) [%.0f - %.0f]",
            mean(df.sub[df.sub$diagnosis == "COMP",]$RAADS_total),
            sd(df.sub[df.sub$diagnosis == "COMP",]$RAADS_total),
            min(df.sub[df.sub$diagnosis == "COMP",]$RAADS_total),
            max(df.sub[df.sub$diagnosis == "COMP",]$RAADS_total)),
    sprintf("%.3f", ostt.RAADS@bayesFactor[["bf"]]),
    sprintf("%.2f (±%.2f) [%.0f - %.0f]",
            mean(df.sub.et[df.sub.et$diagnosis == "ASD",]$RAADS_total),
            sd(df.sub.et[df.sub.et$diagnosis == "ASD",]$RAADS_total),
            min(df.sub.et[df.sub.et$diagnosis == "ASD",]$RAADS_total),
            max(df.sub.et[df.sub.et$diagnosis == "ASD",]$RAADS_total)),
    sprintf("%.2f (±%.2f) [%.0f - %.0f]",
            mean(df.sub.et[df.sub.et$diagnosis == "COMP",]$RAADS_total),
            sd(df.sub.et[df.sub.et$diagnosis == "COMP",]$RAADS_total),
            min(df.sub.et[df.sub.et$diagnosis == "COMP",]$RAADS_total),
            max(df.sub.et[df.sub.et$diagnosis == "COMP",]$RAADS_total)),
    sprintf("%.3f", ostt.RAADS.et@bayesFactor[["bf"]])
  ),
  c(
    "D2 - concentration performance",
    sprintf("%.2f (±%.2f) [%.0f - %.0f]",
            mean(df.sub[df.sub$diagnosis == "ASD",]$sw_kl),
            sd(df.sub[df.sub$diagnosis == "ASD",]$sw_kl),
            min(df.sub[df.sub$diagnosis == "ASD",]$sw_kl),
            max(df.sub[df.sub$diagnosis == "ASD",]$sw_kl)),
    sprintf("%.2f (±%.2f) [%.0f - %.0f]",
```

```
              mean(df.sub[df.sub$diagnosis == "COMP",]$sw_kl),
              sd(df.sub[df.sub$diagnosis == "COMP",]$sw_kl),
              min(df.sub[df.sub$diagnosis == "COMP",]$sw_kl),
              max(df.sub[df.sub$diagnosis == "COMP",]$sw_kl)),
      sprintf("%.3f", ostt.kl@bayesFactor[["bf"]]),
      sprintf("%.2f (±%.2f) [%.0f - %.0f]",
              mean(df.sub.et[df.sub.et$diagnosis == "ASD",]$sw_kl),
              sd(df.sub.et[df.sub.et$diagnosis == "ASD",]$sw_kl),
              min(df.sub.et[df.sub.et$diagnosis == "ASD",]$sw_kl),
              max(df.sub.et[df.sub.et$diagnosis == "ASD",]$sw_kl)),
      sprintf("%.2f (±%.2f) [%.0f - %.0f]",
              mean(df.sub.et[df.sub.et$diagnosis == "COMP",]$sw_kl),
              sd(df.sub.et[df.sub.et$diagnosis == "COMP",]$sw_kl),
              min(df.sub.et[df.sub.et$diagnosis == "COMP",]$sw_kl),
              max(df.sub.et[df.sub.et$diagnosis == "COMP",]$sw_kl)),
      sprintf("%.3f", ostt.kl.et@bayesFactor[["bf"]])
    ),
    c(
      "D2 - speed",
      sprintf("%.2f (±%.2f) [%.0f - %.0f]",
              mean(df.sub[df.sub$diagnosis == "ASD",]$sw_gz),
              sd(df.sub[df.sub$diagnosis == "ASD",]$sw_gz),
              min(df.sub[df.sub$diagnosis == "ASD",]$sw_gz),
              max(df.sub[df.sub$diagnosis == "ASD",]$sw_gz)),
      sprintf("%.2f (±%.2f) [%.0f - %.0f]",
              mean(df.sub[df.sub$diagnosis == "COMP",]$sw_gz),
              sd(df.sub[df.sub$diagnosis == "COMP",]$sw_gz),
              min(df.sub[df.sub$diagnosis == "COMP",]$sw_gz),
              max(df.sub[df.sub$diagnosis == "COMP",]$sw_gz)),
      sprintf("%.3f", ostt.gz@bayesFactor[["bf"]]),
      sprintf("%.2f (±%.2f) [%.0f - %.0f]",
              mean(df.sub.et[df.sub.et$diagnosis == "ASD",]$sw_gz),
              sd(df.sub.et[df.sub.et$diagnosis == "ASD",]$sw_gz),
              min(df.sub.et[df.sub.et$diagnosis == "ASD",]$sw_gz),
              max(df.sub.et[df.sub.et$diagnosis == "ASD",]$sw_gz)),
      sprintf("%.2f (±%.2f) [%.0f - %.0f]",
              mean(df.sub.et[df.sub.et$diagnosis == "COMP",]$sw_gz),
              sd(df.sub.et[df.sub.et$diagnosis == "COMP",]$sw_gz),
              min(df.sub.et[df.sub.et$diagnosis == "COMP",]$sw_gz),
              max(df.sub.et[df.sub.et$diagnosis == "COMP",]$sw_gz)),
      sprintf("%.3f", ostt.gz.et@bayesFactor[["bf"]])
    ),
    c(
      "STAI-trait",
      sprintf("%.2f (±%.2f) [%.0f - %.0f]",
              mean(df.sub[df.sub$diagnosis == "ASD",]$STAITT_total),
              sd(df.sub[df.sub$diagnosis == "ASD",]$STAITT_total),
              min(df.sub[df.sub$diagnosis == "ASD",]$STAITT_total),
              max(df.sub[df.sub$diagnosis == "ASD",]$STAITT_total)),
      sprintf("%.2f (±%.2f) [%.0f - %.0f]",
              mean(df.sub[df.sub$diagnosis == "COMP",]$STAITT_total),
              sd(df.sub[df.sub$diagnosis == "COMP",]$STAITT_total),
              min(df.sub[df.sub$diagnosis == "COMP",]$STAITT_total),
```

```
                max(df.sub[df.sub$diagnosis == "COMP",]$STAITT_total)),
        sprintf("%.3f", ostt.STAITT@bayesFactor[["bf"]]),
        sprintf("%.2f (±%.2f) [%.0f - %.0f]",
                mean(df.sub.et[df.sub.et$diagnosis == "ASD",]$STAITT_total),
                sd(df.sub.et[df.sub.et$diagnosis == "ASD",]$STAITT_total),
                min(df.sub.et[df.sub.et$diagnosis == "ASD",]$STAITT_total),
                max(df.sub.et[df.sub.et$diagnosis == "ASD",]$STAITT_total)),
        sprintf("%.2f (±%.2f) [%.0f - %.0f]",
                mean(df.sub.et[df.sub.et$diagnosis == "COMP",]$STAITT_total),
                sd(df.sub.et[df.sub.et$diagnosis == "COMP",]$STAITT_total),
                min(df.sub.et[df.sub.et$diagnosis == "COMP",]$STAITT_total),
                max(df.sub.et[df.sub.et$diagnosis == "COMP",]$STAITT_total)),
        sprintf("%.3f", ostt.STAITT.et@bayesFactor[["bf"]])
      ),
      c(
        "Ishihara",
        sprintf("%.2f (±%.2f) [%.0f - %.0f]",
                mean(df.sub[df.sub$diagnosis == "ASD",]$ISH_total),
                sd(df.sub[df.sub$diagnosis == "ASD",]$ISH_total),
                min(df.sub[df.sub$diagnosis == "ASD",]$ISH_total),
                max(df.sub[df.sub$diagnosis == "ASD",]$ISH_total)),
        sprintf("%.2f (±%.2f) [%.0f - %.0f]",
                mean(df.sub[df.sub$diagnosis == "COMP",]$ISH_total),
                sd(df.sub[df.sub$diagnosis == "COMP",]$ISH_total),
                min(df.sub[df.sub$diagnosis == "COMP",]$ISH_total),
                max(df.sub[df.sub$diagnosis == "COMP",]$ISH_total)),
        sprintf("%.3f", ostt.ISH@bayesFactor[["bf"]]),
        sprintf("%.2f (±%.2f) [%.0f - %.0f]",
                mean(df.sub.et[df.sub.et$diagnosis == "ASD",]$ISH_total),
                sd(df.sub.et[df.sub.et$diagnosis == "ASD",]$ISH_total),
                min(df.sub.et[df.sub.et$diagnosis == "ASD",]$ISH_total),
                max(df.sub.et[df.sub.et$diagnosis == "ASD",]$ISH_total)),
        sprintf("%.2f (±%.2f) [%.0f - %.0f]",
                mean(df.sub.et[df.sub.et$diagnosis == "COMP",]$ISH_total),
                sd(df.sub.et[df.sub.et$diagnosis == "COMP",]$ISH_total),
                min(df.sub.et[df.sub.et$diagnosis == "COMP",]$ISH_total),
                max(df.sub.et[df.sub.et$diagnosis == "COMP",]$ISH_total)),
        sprintf("%.3f", ostt.ISH.et@bayesFactor[["bf"]])
      ),
      c(
        "UI",
        sprintf("%.2f (±%.2f) [%.0f - %.0f]",
                mean(df.sub[df.sub$diagnosis == "ASD",]$UI_total),
                sd(df.sub[df.sub$diagnosis == "ASD",]$UI_total),
                min(df.sub[df.sub$diagnosis == "ASD",]$UI_total),
                max(df.sub[df.sub$diagnosis == "ASD",]$UI_total)),
        sprintf("%.2f (±%.2f) [%.0f - %.0f]",
                mean(df.sub[df.sub$diagnosis == "COMP",]$UI_total),
                sd(df.sub[df.sub$diagnosis == "COMP",]$UI_total),
                min(df.sub[df.sub$diagnosis == "COMP",]$UI_total),
                max(df.sub[df.sub$diagnosis == "COMP",]$UI_total)),
        sprintf("%.3f", ostt.UI@bayesFactor[["bf"]]),
        sprintf("%.2f (±%.2f) [%.0f - %.0f]",
```

```r
                                mean(df.sub.et[df.sub.et$diagnosis == "ASD",]$UI_total),
                                sd(df.sub.et[df.sub.et$diagnosis == "ASD",]$UI_total),
                                min(df.sub.et[df.sub.et$diagnosis == "ASD",]$UI_total),
                                max(df.sub.et[df.sub.et$diagnosis == "ASD",]$UI_total)),
                        sprintf("%.2f (±%.2f) [%.0f - %.0f]",
                                mean(df.sub.et[df.sub.et$diagnosis == "COMP",]$UI_total),
                                sd(df.sub.et[df.sub.et$diagnosis == "COMP",]$UI_total),
                                min(df.sub.et[df.sub.et$diagnosis == "COMP",]$UI_total),
                                max(df.sub.et[df.sub.et$diagnosis == "COMP",]$UI_total)),
                        sprintf("%.3f", ostt.UI.et@bayesFactor[["bf"]])
                      )
                   )

  # save it all
  df = df %>% select(subID, diagnosis, dyad, video, run, trl, sync, rating,
                     dyad.type, mot, peak, rating.confirmed, s1peak, s2peak)
  save(df, df.fix, df.sac.agg,
       df.table, df.sht, df.sht.et, ct.beh, ct.et, df.exc, df.incET, df.asd,
       file = "PESI_data.RData")

} else {

  load("PESI_data.RData")

}

# print the group of included participants
kable(df %>% select(subID, diagnosis) %>% distinct() %>% group_by(diagnosis) %>% count())
```

diagnosis	n
ASD	27
COMP	36

```r
# print the group of excluded participants
kable(df.exc)
```

diagnosis	n
ASD	5
COMP	2

```r
rm(df.exc)

# print number of included participants in eye tracking
kable(df.incET)
```

diagnosis	gender	n
ASD	fem	10
ASD	mal	10
COMP	fem	17
COMP	mal	9

diagnosis	gender	n

```r
rm(df.incET)

# print the icd 10 code counts
kable(df.asd)
```

ASD.icd10	n
F84.0	5
F84.1	2
F84.5	20

```r
rm(df.asd)

# print the outcome of the shapiro tests
kable(df.sht)
```

diagnosis	variable	statistic	p	sig
ASD	BDI_total	0.8733786	0.0034673	*
COMP	BDI_total	0.8548415	0.0002465	*
ASD	CFT_iq	0.9306520	0.0717023	
COMP	CFT_iq	0.9464526	0.0808711	
ASD	ISH_total	0.4654390	0.0000000	*
COMP	ISH_total	0.4929020	0.0000000	*
ASD	RAADS_total	0.8323572	0.0005256	*
COMP	RAADS_total	0.8278027	0.0000611	*
ASD	STAITT_total	0.9502740	0.2176972	
COMP	STAITT_total	0.9638854	0.2824299	
ASD	UI_total	0.9090163	0.0216386	*
COMP	UI_total	0.9019167	0.0038513	*
ASD	age	0.9308549	0.0725292	
COMP	age	0.9395161	0.0490695	*
ASD	sw_gz	0.9456727	0.1680787	
COMP	sw_gz	0.9540447	0.1401784	
ASD	sw_kl	0.9567579	0.3110060	
COMP	sw_kl	0.9353969	0.0365896	*

```r
rm(df.sht)
kable(df.sht.et)
```

diagnosis	variable	statistic	p	sig
ASD	BDI_total	0.8242338	0.0020323	*
COMP	BDI_total	0.8634980	0.0026292	*
ASD	CFT_iq	0.9416794	0.2579128	
COMP	CFT_iq	0.9588648	0.3696914	
ASD	ISH_total	0.4563704	0.0000001	*
COMP	ISH_total	0.6693335	0.0000021	*
ASD	RAADS_total	0.7594585	0.0002280	*
COMP	RAADS_total	0.9005759	0.0161271	*

diagnosis	variable	statistic	p	sig
ASD	STAITT_total	0.9602915	0.5496893	
COMP	STAITT_total	0.9287707	0.0724445	
ASD	UI_total	0.8538946	0.0061989	*
COMP	UI_total	0.8102438	0.0002643	*
ASD	age	0.9325877	0.1731580	
COMP	age	0.9228331	0.0524119	
ASD	sw_gz	0.9145795	0.0779632	
COMP	sw_gz	0.9563677	0.3251132	
ASD	sw_kl	0.9242727	0.1197645	
COMP	sw_kl	0.9313464	0.0834328	

```
rm(df.sht.et)

# print the outcome of the contingency table for behavioural sample
ct.beh@bayesFactor
```

```
##                             bf error                     time         code
## Non-indep. (a=1) -0.7457339     0 Wed Apr  9 14:24:29 2025 43491cbfcf90
```

```
# print the outcome of the contingency table for ET sample
ct.et@bayesFactor
```

```
##                             bf error                     time          code
## Non-indep. (a=1) -0.5166516     0 Wed Apr  9 14:24:29 2025 43491313e6d67
```

```
# print information on how many trials were included per participant
df.trlno = df %>%
  filter(!is.na(rating.confirmed)) %>%
  group_by(subID, diagnosis) %>%
  count()
df.trlno %>%
  group_by(diagnosis) %>%
  summarise(
    trlno.mean = mean(n),
    trlno.sd   = sd(n),
    trlno.se   = trlno.sd/sqrt(n()),
    trlno.min  = min(n),
    trlno.max  = max(n)
  )
```

```
## # A tibble: 2 x 6
##   diagnosis trlno.mean trlno.sd trlno.se trlno.min trlno.max
##   <fct>          <dbl>    <dbl>    <dbl>     <int>     <int>
## 1 ASD             59.9     4.84    0.932        46        64
## 2 COMP            60.1     5.54    0.923        43        64
```

```
# is there a difference between groups?
df.trlno %>%
    group_by(diagnosis) %>%
    shapiro_test(n) %>%
    arrange(variable) %>%
    mutate(
      sig = if_else(p < 0.05, "*", "")
    )
```

```
## # A tibble: 2 x 5
##   diagnosis variable statistic           p sig
##   <fct>     <chr>        <dbl>       <dbl> <chr>
## 1 ASD       n            0.808 0.000191    *
## 2 COMP      n            0.727 0.000000779 *
```

```
df.trlno = df.trlno %>%
  ungroup() %>%
  mutate(
    rn   = rank(n)
    )
ostt.trlno = ttestBF(formula = rn ~ diagnosis, data = df.trlno)
ostt.trlno@bayesFactor
```

```
##                        bf        error                     time         code
## Alt., r=0.707 -1.192466 0.0001071183 Wed Apr 16 11:19:07 2025 4edd5d594042
```

```
effectsize::interpret_bf(ostt.trlno@bayesFactor$bf, log = T)
```

```
## [1] "moderate evidence against"
## (Rules: jeffreys1961)
```

```
# aggregate the data due to large differences between videos
df.agg = df %>%
  group_by(subID, diagnosis, dyad, sync, dyad.type) %>%
  summarise(
    rating.confirmed = mean(rating.confirmed, na.rm = T)
  ) %>% ungroup() %>%
  mutate_if(is.character, as.factor)
df.fix.agg = df.fix %>%
  group_by(subID, diagnosis, dyad, sync, dyad.type, AOI) %>%
  summarise(
    fix.dur   = median(fix.dur, na.rm = T),
    fix.total = median(fix.total),
    fix.prop  = fix.dur*100/fix.total
  ) %>% ungroup() %>%
  mutate_if(is.character, as.factor)

# set and print the contrasts
contrasts(df.agg$sync) = contr.sum(2)
contrasts(df.agg$sync)
```

```
##      [,1]
## high    1
## low    -1
```

```
contrasts(df.agg$dyad.type) = contr.sum(2)
contrasts(df.agg$dyad.type)
```

```
##              [,1]
## mixed           1
## non-autistic   -1
```

```
contrasts(df.agg$diagnosis) = contr.sum(2)
contrasts(df.agg$diagnosis)
```

```
##      [,1]
## ASD     1
```

```
## COMP   -1
```

```r
contrasts(df.fix$sync) = contr.sum(2)
contrasts(df.fix$sync)
```

```
##      [,1]
## high    1
## low    -1
```

```r
contrasts(df.fix$dyad.type) = contr.sum(2)
contrasts(df.fix$dyad.type)
```

```
##              [,1]
## mixed           1
## non-autistic   -1
```

```r
contrasts(df.fix$diagnosis) = contr.sum(2)
contrasts(df.fix$diagnosis)
```

```
##      [,1]
## ASD     1
## COMP   -1
```

```r
contrasts(df.fix.agg$sync) = contr.sum(2)
contrasts(df.fix.agg$sync)
```

```
##      [,1]
## high    1
## low    -1
```

```r
contrasts(df.fix.agg$dyad.type) = contr.sum(2)
contrasts(df.fix.agg$dyad.type)
```

```
##              [,1]
## mixed           1
## non-autistic   -1
```

```r
contrasts(df.fix.agg$diagnosis) = contr.sum(2)
contrasts(df.fix.agg$diagnosis)
```

```
##      [,1]
## ASD     1
## COMP   -1
```

```r
contrasts(df.fix.agg$AOI) = contr.sum(3)[c(3,2,1),]
contrasts(df.fix.agg$AOI)
```

```
##      [,1] [,2]
## body   -1   -1
## hand    0    1
## head    1    0
```

```r
contrasts(df.sac.agg$sync) = contr.sum(2)
contrasts(df.sac.agg$sync)
```

```
##      [,1]
## high    1
## low    -1
```

```r
contrasts(df.sac.agg$dyad.type) = contr.sum(2)
contrasts(df.sac.agg$dyad.type)
```

```
##               [,1]
## mixed            1
## non-autistic    -1
```

```r
contrasts(df.sac.agg$diagnosis) = contr.sum(2)
contrasts(df.sac.agg$diagnosis)
```

```
##      [,1]
## ASD     1
## COMP   -1
```

Stimulus comparison

```r
df.stm = df %>%
  ungroup() %>%
  select(video, sync, dyad.type, mot, peak) %>%
  distinct()

# differences in sync and motion by sync
df.stm %>%
  group_by(sync) %>%
  summarise(
    peak.mean = mean(peak),
    peak.sd   = sd(peak),
    mot.mean  = mean(mot),
    mot.sd    = sd(mot)
  )
```

```
## # A tibble: 2 x 5
##   sync  peak.mean peak.sd mot.mean mot.sd
##   <fct>     <dbl>   <dbl>    <dbl>  <dbl>
## 1 high      0.771  0.160     0.531 0.0758
## 2 low       0.276  0.0706    0.520 0.0672
```

```r
df.stm %>%
  group_by(sync) %>%
  shapiro_test(
    peak, mot
  )
```

```
## # A tibble: 4 x 4
##   sync  variable statistic     p
##   <fct> <chr>        <dbl> <dbl>
## 1 high  mot          0.952 0.162
## 2 high  peak         0.973 0.589
## 3 low   mot          0.969 0.465
## 4 low   peak         0.972 0.542
```

```r
t.mot = ttestBF(df.stm[df.stm$sync == "high",]$mot,
                df.stm[df.stm$sync == "low",]$mot, paired = F)
t.mot
```

```
## Bayes factor analysis
```

```
## --------------
## [1] Alt., r=0.707 : 0.2991656 ±0.01%
##
## Against denominator:
##   Null, mu1-mu2 = 0
## ---
## Bayes factor type: BFindepSample, JZS
```

```r
t.mot@bayesFactor$bf
```

```
## [1] -1.206758
```

```r
t.peak = ttestBF(df.stm[df.stm$sync == "high",]$peak,
                 df.stm[df.stm$sync == "low",]$peak, paired = F)
t.peak
```

```
## Bayes factor analysis
## --------------
## [1] Alt., r=0.707 : 2.053393e+20 ±0%
##
## Against denominator:
##   Null, mu1-mu2 = 0
## ---
## Bayes factor type: BFindepSample, JZS
```

```r
t.peak@bayesFactor$bf
```

```
## [1] 46.7712
```

```r
# differences in sync and motion by dyad type
df.stm %>%
  group_by(dyad.type) %>%
  summarise(
    peak.mean = mean(peak),
    peak.sd   = sd(peak),
    mot.mean  = mean(mot),
    mot.sd    = sd(mot)
  )
```

```
## # A tibble: 2 x 5
##   dyad.type    peak.mean peak.sd mot.mean mot.sd
##   <fct>            <dbl>   <dbl>    <dbl>  <dbl>
## 1 mixed            0.520   0.292    0.527 0.0583
## 2 non-autistic     0.528   0.267    0.523 0.0831
```

```r
df.stm %>%
  group_by(dyad.type) %>%
  shapiro_test(
    peak, mot
  )
```

```
## # A tibble: 4 x 4
##   dyad.type    variable statistic       p
##   <fct>        <chr>        <dbl>   <dbl>
## 1 mixed        mot          0.985 0.925
## 2 mixed        peak         0.899 0.00600
## 3 non-autistic mot          0.920 0.0210
## 4 non-autistic peak         0.909 0.0103
```

```r
df.stm = df.stm %>%
  ungroup() %>%
  mutate(
    rpeak = rank(peak),
    rmot  = rank(mot)
  )

t.mot = ttestBF(df.stm[df.stm$dyad.type == "mixed",]$rmot,
                df.stm[df.stm$dyad.type == "non-autistic",]$rmot, paired = F)
t.mot
```

```
## Bayes factor analysis
## --------------
## [1] Alt., r=0.707 : 0.2620788 ±0.01%
##
## Against denominator:
##   Null, mu1-mu2 = 0
## ---
## Bayes factor type: BFindepSample, JZS
```

```r
t.mot@bayesFactor$bf
```

```
## [1] -1.33911
```

```r
t.peak = ttestBF(df.stm[df.stm$dyad.type == "mixed",]$rpeak,
                 df.stm[df.stm$dyad.type == "non-autistic",]$rpeak, paired = F)
t.peak
```

```
## Bayes factor analysis
## --------------
## [1] Alt., r=0.707 : 0.2733605 ±0.01%
##
## Against denominator:
##   Null, mu1-mu2 = 0
## ---
## Bayes factor type: BFindepSample, JZS
```

```r
t.peak@bayesFactor$bf
```

```
## [1] -1.296964
```

Hypothesis-guided analysis

Aggregated ratings

After each video, participants were asked to rate the interaction on a continuous scale for "How comfortable do you imagine this interaction to be?" (from 0 "not at all" to 100 "very").

SBC

To achieve good posterior fit, we aggregated the rating data. First, we attempted to run a model including all main effects as slopes as well as the interaction of sync and dyad type for subjects. However, all models including random slopes had major divergence issues. Even the model only including the intercepts on the group-level had some divergent transitions. Nonetheless, we opted to include both the intercept for the dyads and the subjects, and look out for problems with the actual model.

```r
# set formula considering all combinations
code   = "PESI_int"
f.pesi = brms::bf(rating.confirmed ~ diagnosis * sync * dyad.type
                  + (1 | subID) + (1 | dyad))

# set weakly informed priors
priors = c(
  prior(normal(50,  10), class = Intercept),
  prior(normal(0,   10), class = sigma),
  prior(normal(0,   10), class = sd),
  # differences due to dyad.type
  prior(normal(-5,   5), class = b, coef = dyad.type1), # mixed
  # differences due to synchrony
  prior(normal(5,    5), class = b, coef = sync1), # high
  # effect of synchrony decreased when mixed dyad
  prior(normal(-5,   5), class = b, coef = sync1:dyad.type1),
  # effect of dyad type decreased in autistic subjects
  prior(normal(-5,   5), class = b, coef = diagnosis1:dyad.type1),
  # no specific expectations for diagnostic groups and other interactions
  prior(normal(0,    5), class = b)
)

if (file.exists(file.path(cache_dir, paste0("df_res_", code, ".rds")))) {
  # load in the results of the SBC
  df.results = readRDS(file.path(cache_dir, paste0("df_res_", code, ".rds")))
  df.backend = readRDS(file.path(cache_dir, paste0("df_div_", code, ".rds")))
  dat        = readRDS(file.path(cache_dir, paste0("dat_", code, ".rds")))
} else {
  # set the seed
  set.seed(2469)
  # create the data
  gen  = SBC_generator_brms(f.pesi, data = df.agg, prior = priors,
                            thin = 50, warmup = 20000, refresh = 2000
  )
  dat  = generate_datasets(gen, nsim)
  saveRDS(dat, file = sprintf("%s/dat_%s.rds", cache_dir, code))

  # perform the SBC
  bck = SBC_backend_brms_from_generator(gen, chains = 4, thin = 1,
                                        warmup = warm, iter = iter,
                                        inits = 0.1)
  res = compute_SBC(dat, bck,
                    cache_mode = "results",
                    cache_location = file.path(cache_dir, sprintf("res_%s", code)))
  # save the results dataframes
  df.results = res$stats
  df.backend = res$backend_diagnostics
  saveRDS(df.results, file = file.path(cache_dir, paste0("df_res_", code, ".rds")))
  saveRDS(df.backend, file = file.path(cache_dir, paste0("df_div_", code, ".rds")))

}
```

We start by investigating the rhats and the number of divergent samples. This shows that 14 of 500 simulations had at least one parameter that had an rhat of at least 1.05, and 186 models had divergent samples (mean

number of samples of the simulations with divergent samples: 7.85). We will have to watch out for this.

Next, we can plot the simulated values to perform prior predictive checks.

```
# create a matrix out of generated data
dvname = gsub(" ", "", gsub("[\\|~].*", "", f.pesi)[1])
dvfakemat = matrix(NA, nrow(dat[['generated']][[1]]), length(dat[['generated']]))
for (i in 1:length(dat[['generated']])) {
  dvfakemat[,i] = dat[['generated']][[i]][[dvname]]
}
truePars = dat$variables

# plot simulated data for prior predictive checks
dvmax = 100
dvfakematH = dvfakemat;
dvfakematH[dvfakematH > dvmax] = dvmax
dvfakematH[dvfakematH < 0] = 0
breaks = seq(0, max(dvfakematH, na.rm=T), length.out = 100)
binwidth = round(breaks[2] - breaks[1])
breaks = seq(0, max(dvfakematH), by = binwidth)
histmat = matrix(NA, ncol = dim(dvfakematH)[2] + binwidth, nrow = length(breaks)-1)
for (i in 1:dim(dvfakematH)[2]) {
  histmat[,i] = hist(dvfakematH[,i], breaks = breaks, plot = F)$counts
}
probs = seq(0.1, 0.9, 0.1)
quantmat= as.data.frame(matrix(NA, nrow=dim(histmat)[1], ncol = length(probs)))
names(quantmat) = paste0("p", probs)
for (i in 1:dim(histmat)[1]) {
  quantmat[i,] = quantile(histmat[i,], p = probs, na.rm = T)
}
quantmat$x = breaks[2:length(breaks)] - binwidth/2 # add bin mean
ggplot(data = quantmat, aes(x = x)) +
  geom_ribbon(aes(ymax = p0.9, ymin = p0.1), fill = c_light) +
  geom_ribbon(aes(ymax = p0.8, ymin = p0.2), fill = c_light_highlight) +
  geom_ribbon(aes(ymax = p0.7, ymin = p0.3), fill = c_mid) +
  geom_ribbon(aes(ymax = p0.6, ymin = p0.4), fill = c_mid_highlight) +
  geom_line(aes(y = p0.5), colour = c_dark, linewidth = 1) +
  xlim(0, max(dvfakematH)) +
  theme_bw()
```

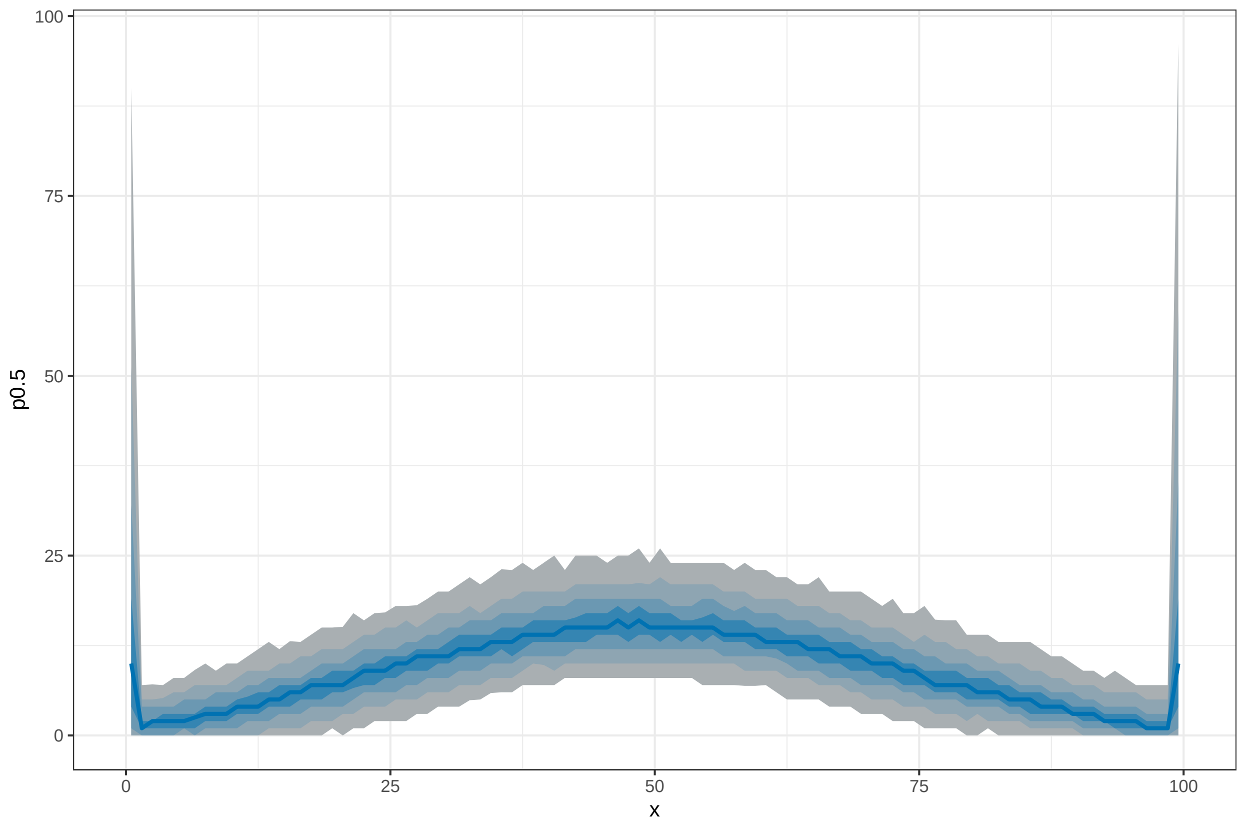

The above figure shows the simulated data for the ratings. The data is centered around the middle of the rating scale. We had some outliers that were outside of the possible values (greater than 100 or smaller than 0), however, to keep our prior somewhat wide we chose to accept this in the simulated data.

```
# get simulation numbers with issues
rank = max(df.results$max_rank)
check = merge(df.results %>%
    group_by(sim_id) %>%
      summarise(
        rhat = max(rhat, na.rm = T),
        mean_rank = mean(max_rank)
        ) %>%
    filter(rhat >= 1.05 | mean_rank != rank),
  df.backend %>% filter(n_divergent > 0), all = T)

# plot SBC with functions from the SBC package focusing on population-level parameters
df.results.b = df.results %>%
  filter(substr(variable, 1, 2) == "b_") %>%
  filter(!(sim_id %in% check$sim_id))
p1 = plot_ecdf_diff(df.results.b) + theme_bw() + theme(legend.position = "none") +
  scale_x_continuous(breaks=scales::pretty_breaks(n = 3)) +
  scale_y_continuous(breaks=scales::pretty_breaks(n = 3))
p2 = plot_rank_hist(df.results.b, bins = 20) + theme_bw() +
  scale_x_continuous(breaks=scales::pretty_breaks(n = 3)) +
  scale_y_continuous(breaks=scales::pretty_breaks(n = 3))
p3 = plot_sim_estimated(df.results.b, alpha = 0.5) + theme_bw() +
  scale_x_continuous(breaks=scales::pretty_breaks(n = 3)) +
  scale_y_continuous(breaks=scales::pretty_breaks(n = 3))
```

```r
p4 = plot_contraction(
  df.results.b,
  prior_sd = setNames(c(10, # Intercept
                        rep(5, length(unique(df.results.b$variable))-1)),
                      unique(df.results.b$variable))) +
  theme_bw() +
  scale_x_continuous(breaks=scales::pretty_breaks(n = 3)) +
  scale_y_continuous(breaks=scales::pretty_breaks(n = 3))

p = ggarrange(p1, p2, p3, p4, labels = "AUTO", ncol = 1, nrow = 4)
annotate_figure(p,
                top = text_grob("Computational faithfulness and model sensitivity",
                face = "bold", size = 14))
```

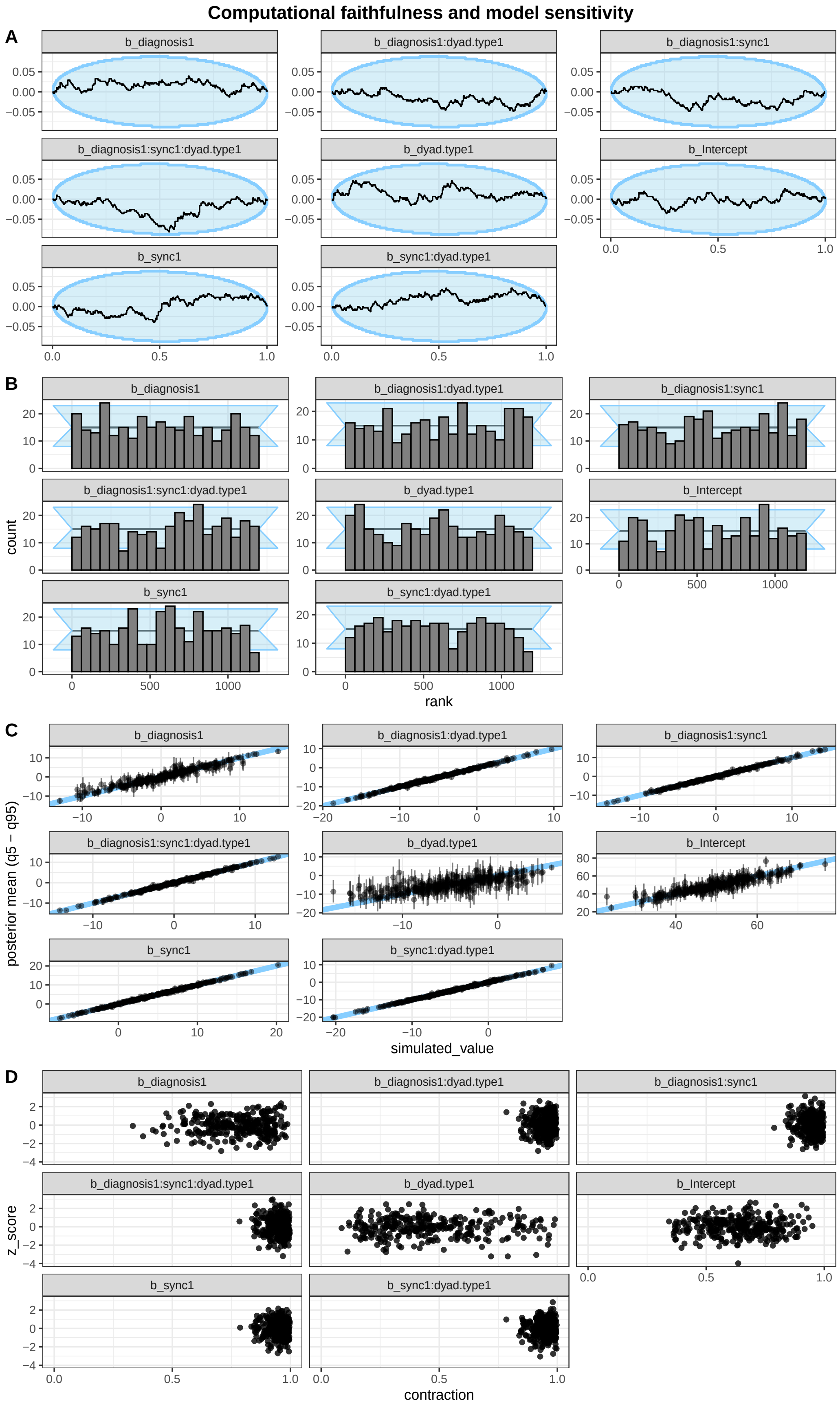

Computational faithfulness and model sensitivity
A
b_diagnosis1
b_diagnosis1:dyad.type1
b_diagnosis1:sync1
b_diagnosis1:sync1:dyad.type1
b_dyad.type1
b_Intercept
b_sync1
b_sync1:dyad.type1
B
count
rank
C
posterior mean (q5 – q95)
simulated_value
D
z_score
contraction

Second, we check the ranks of the parameters. If the model is unbiased, these should be uniformly distributed (Schad, Betancourt and Vasishth, 2020). The sample empirical cumulative distribution function (ECDF) lies within the theoretical distribution (95%) and the rank histogram also shows ranks within the 95% expected range, although there are some small deviations. We judge this to be acceptable.

Third, we investigated the relationship between the simulated true parameters and the posterior estimates. Although there are individual values diverging from the expected pattern, most parameters were recovered successfully within an uncertainty interval of alpha = 0.05.

Last, we explore the z-score and the posterior contraction of our population-level predictors. The z-score "determines the distance of the posterior mean from the true simulating parameter", while the posterior contraction "estimates how much prior uncertainty is reduced in the posterior estimation" (Schad, Betancourt and Vasisth, 2020). Both look acceptable.

Posterior predictive checks

As the next step, we fit the model and check whether there were any obvious issues with it.

```
# fit the model
set.seed(2486)
m.pesi = brm(f.pesi,
            df.agg, prior = priors,
            iter = iter, warmup = warm,
            backend = "cmdstanr", threads = threading(8),
            file = "m_PESI",
            save_pars = save_pars(all = TRUE)
            )

# in this model, there are no divergent samples
sum(subset(nuts_params(m.pesi), Parameter == "divergent__")$Value)
```

```
## [1] 0
```

```
# check that rhats are below 1.01
sum(brms::rhat(m.pesi) >= 1.01, na.rm = T)
```

```
## [1] 0
```

```
# and the chains have converged
post.draws = as_draws_df(m.pesi)
mcmc_trace(post.draws, regex_pars = "^b_",
           facet_args = list(ncol = 2)) +
  scale_x_continuous(breaks=scales::pretty_breaks(n = 3)) +
  scale_y_continuous(breaks=scales::pretty_breaks(n = 3))
```

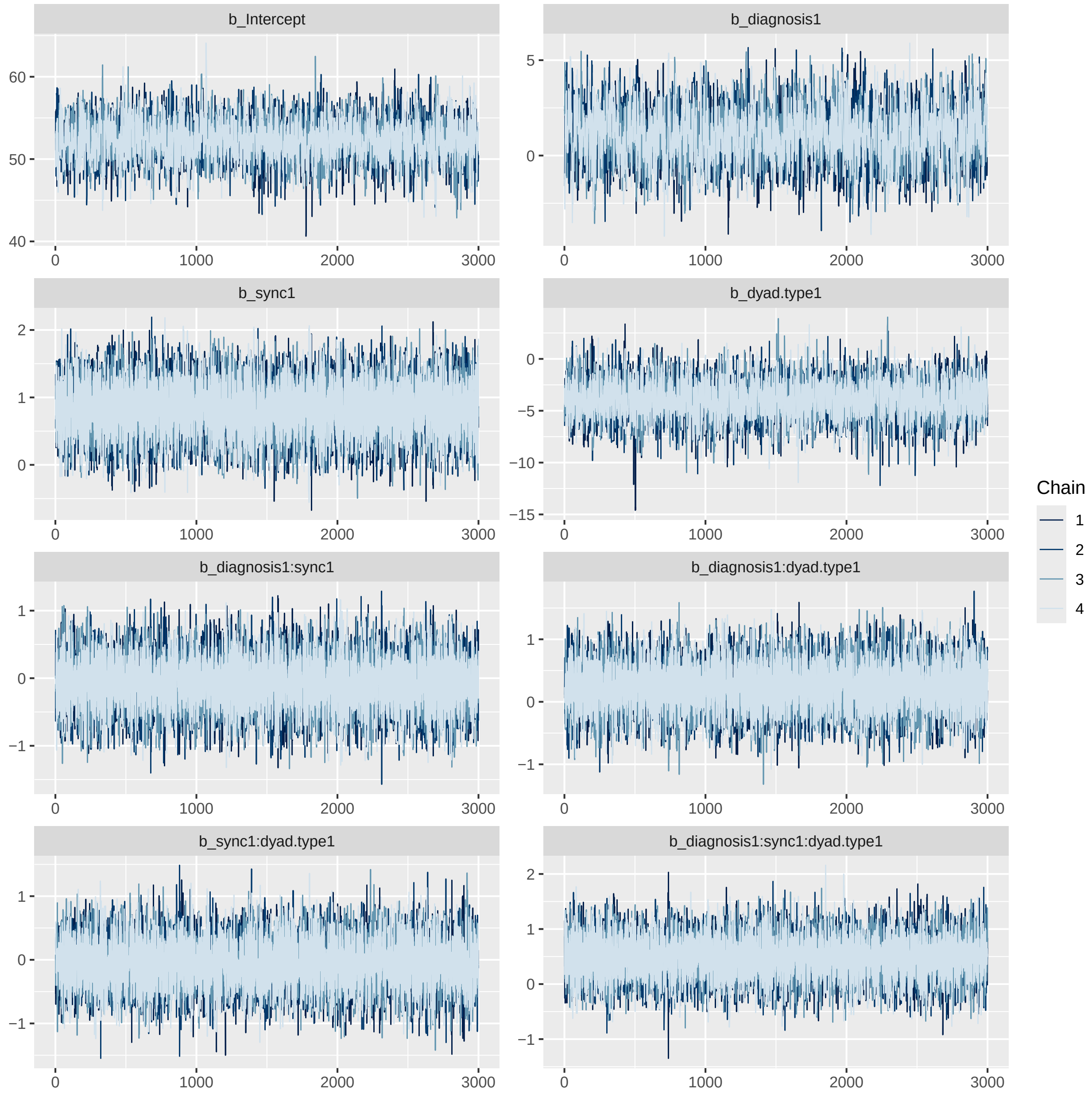

This model has no divergent samples, and no rhat that is higher or equal to 1.01. Therefore, we go ahead and perform our posterior predictive checks.

```
# get the posterior predictions
post.pred = posterior_predict(m.pesi, ndraws = nsim)

# check the fit of the predicted data compared to the real data
p1 = pp_check(m.pesi, ndraws = nsim) +
  theme_bw() + theme(legend.position = "none") + xlim(0,100)

# distributions of means and sds compared to the real values per group
p2 = ppc_stat_grouped(df.agg$rating.confirmed, post.pred, df.agg$diagnosis) +
  theme_bw() + theme(legend.position = "none")
# ... sync level
p3 = ppc_stat_grouped(df.agg$rating.confirmed, post.pred, df.agg$sync) +
```

```
  theme_bw() + theme(legend.position = "none")
# ... and dyad type
p4 = ppc_stat_grouped(df.agg$rating.confirmed, post.pred, df.agg$dyad.type) +
  theme_bw() + theme(legend.position = "none")

p = ggarrange(p1, p2, p3, p4,
          nrow = 2, ncol = 2, labels = "AUTO")
annotate_figure(p,
                top = text_grob("Posterior predictive checks",
                face = "bold", size = 14))
```

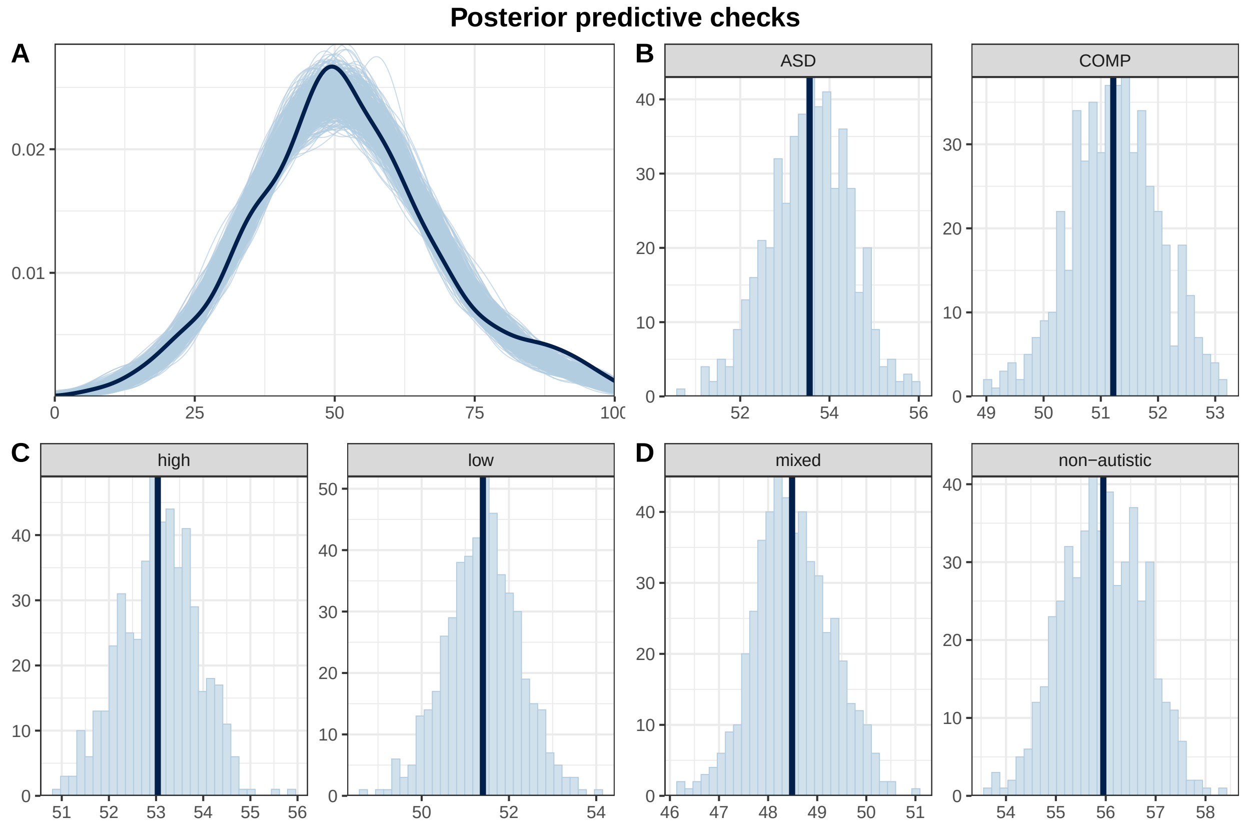

The predictions based on the model (light blue) capture the data (dark blue) very well. The predicted means for each group are firmly distributed around the real values. This further increased our trust in the model and we move on to interpret it.

Model summary

Now that we are convinced that we can trust our model, we have a look at the model and its estimates.

```
# print a summary
summary(m.pesi)
```

```
##  Family: gaussian
##   Links: mu = identity; sigma = identity
## Formula: rating.confirmed ~ diagnosis * sync * dyad.type + (1 | subID) + (1 | dyad)
##    Data: df.agg (Number of observations: 1008)
##   Draws: 4 chains, each with iter = 4500; warmup = 1500; thin = 1;
```

```
##          total post-warmup draws = 12000
##
## Multilevel Hyperparameters:
## ~dyad (Number of levels: 8)
##               Estimate Est.Error l-95% CI u-95% CI Rhat Bulk_ESS Tail_ESS
## sd(Intercept)     5.18      1.78     2.77     9.62 1.00     4251     6415
##
## ~subID (Number of levels: 63)
##               Estimate Est.Error l-95% CI u-95% CI Rhat Bulk_ESS Tail_ESS
## sd(Intercept)    10.74      1.07     8.87    13.00 1.00     3107     5369
##
## Regression Coefficients:
##                             Estimate Est.Error l-95% CI u-95% CI Rhat Bulk_ESS
## Intercept                      52.24      2.32    47.65    56.84 1.00     2930
## diagnosis1                      1.07      1.37    -1.61     3.78 1.00     2137
## sync1                           0.83      0.40     0.02     1.60 1.00    22391
## dyad.type1                     -3.87      1.78    -7.45    -0.40 1.00     5143
## diagnosis1:sync1               -0.08      0.40    -0.87     0.71 1.00    22927
## diagnosis1:dyad.type1           0.24      0.39    -0.54     1.02 1.00    21273
## sync1:dyad.type1               -0.06      0.40    -0.83     0.73 1.00    22302
## diagnosis1:sync1:dyad.type1     0.48      0.40    -0.30     1.24 1.00    21095
##                             Tail_ESS
## Intercept                       5158
## diagnosis1                      4105
## sync1                           7815
## dyad.type1                      6811
## diagnosis1:sync1                8372
## diagnosis1:dyad.type1           8337
## sync1:dyad.type1                8364
## diagnosis1:sync1:dyad.type1     8549
##
## Further Distributional Parameters:
##       Estimate Est.Error l-95% CI u-95% CI Rhat Bulk_ESS Tail_ESS
## sigma    12.56      0.29    12.00    13.15 1.00    17960     9039
##
## Draws were sampled using sample(hmc). For each parameter, Bulk_ESS
## and Tail_ESS are effective sample size measures, and Rhat is the potential
## scale reduction factor on split chains (at convergence, Rhat = 1).
```

```r
# plot the posterior distributions for the estimated effects
post.draws %>%
  select(starts_with("b_")) %>%
  pivot_longer(cols = starts_with("b_"),
               names_to = "coef",
               values_to = "estimate") %>%
  subset(!startsWith(coef, "b_Int")) %>%
  mutate(
    coef = substr(coef, 3, nchar(coef)),
    coef = str_replace_all(coef, ":", " x "),
    coef = str_replace_all(coef, "diagnosis1", "ASD"),
    coef = str_replace_all(coef, "sync1", "high sync"),
    coef = str_replace_all(coef, "dyad.type1", "mixed"),
    coef_order = case_when(
      coef == "ASD" ~ 100,
```

```
      coef == "high sync" ~ 99,
      coef == "mixed" ~ 98,
      T ~ 100-nchar(coef)),
    coef = fct_reorder(coef, coef_order)
  ) %>%
  group_by(coef) %>%
  mutate(
    cred = case_when(
      (mean(estimate) < 0 & quantile(estimate, probs = 0.975) < 0) |
        (mean(estimate) > 0 & quantile(estimate, probs = 0.025) > 0) ~ "credible",
      T ~ "not credible"
    )
  ) %>% ungroup() %>%
  ggplot(aes(x = estimate, y = coef, fill = cred)) +
  geom_vline(xintercept = 0, linetype = 'dashed') +
  ggdist::stat_halfeye(alpha = 0.7) + ylab(NULL) + theme_bw() +
  scale_fill_manual(values = c(credible = c_dark, c_light)) +
  theme(legend.position = "none")
```

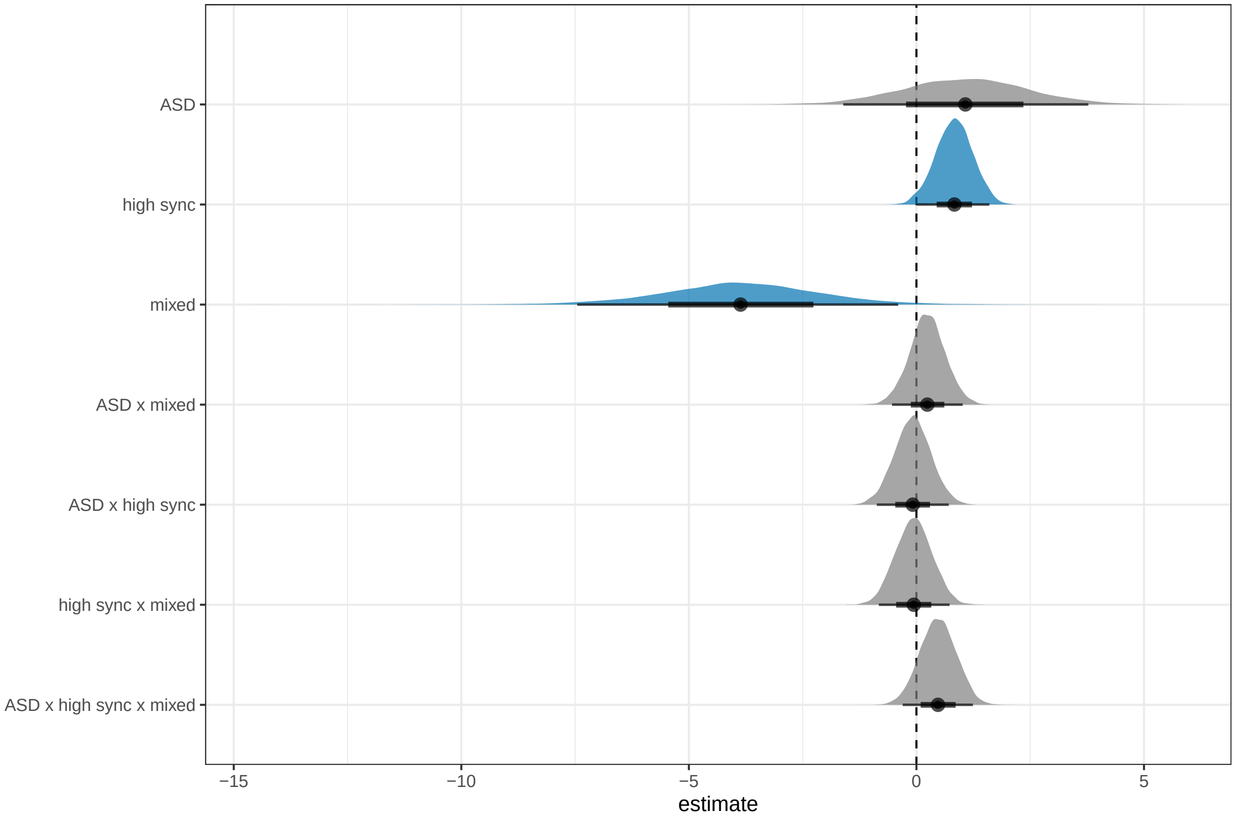

Inferences

```
# H1.a Context: Social interactions of no-diagnosis non-autistic dyads are
# rated more positively than mixed-diagnosis dyads consisting of one autistic
# and one non-autistic interaction partner.
h1.a = hypothesis(m.pesi, "dyad.type1 < 0")
```

```
h1.a
```

```
## Hypothesis Tests for class b:
##          Hypothesis Estimate Est.Error CI.Lower CI.Upper Evid.Ratio Post.Prob
## 1 (dyad.type1) < 0    -3.87      1.78    -6.79    -1.01      59.91      0.98
##   Star
## 1    *
## ---
## 'CI': 90%-CI for one-sided and 95%-CI for two-sided hypotheses.
## '*': For one-sided hypotheses, the posterior probability exceeds 95%;
## for two-sided hypotheses, the value tested against lies outside the 95%-CI.
## Posterior probabilities of point hypotheses assume equal prior probabilities.
```

```
# H1.b Synchrony: Social interactions with high interpersonal synchrony of
# motion energy are rated more positively than social interactions with low
# interpersonal synchrony of motion energy.
h1.b = hypothesis(m.pesi, "sync1 > 0")
h1.b
```

```
## Hypothesis Tests for class b:
##    Hypothesis Estimate Est.Error CI.Lower CI.Upper Evid.Ratio Post.Prob Star
## 1 (sync1) > 0     0.83       0.4     0.16     1.49      43.78      0.98    *
## ---
## 'CI': 90%-CI for one-sided and 95%-CI for two-sided hypotheses.
## '*': For one-sided hypotheses, the posterior probability exceeds 95%;
## for two-sided hypotheses, the value tested against lies outside the 95%-CI.
## Posterior probabilities of point hypotheses assume equal prior probabilities.
```

```
# H1.c Diagnostic status: Ratings of social interactions differ between
# autistic and comparison observers.
h1.c = hypothesis(m.pesi, "diagnosis1 > 0", alpha = 0.025)
h1.c
```

```
## Hypothesis Tests for class b:
##          Hypothesis Estimate Est.Error CI.Lower CI.Upper Evid.Ratio Post.Prob
## 1 (diagnosis1) > 0     1.07      1.37    -1.61     3.78       3.67      0.79
##   Star
## 1
## ---
## 'CI': 95%-CI for one-sided and 97.5%-CI for two-sided hypotheses.
## '*': For one-sided hypotheses, the posterior probability exceeds 97.5%;
## for two-sided hypotheses, the value tested against lies outside the 97.5%-CI.
## Posterior probabilities of point hypotheses assume equal prior probabilities.
```

```
# H1.d Synchrony x dyad type: The effect of interpersonal motion synchrony
# on ratings is decreased for mixed-diagnosis dyads compared to no-diagnosis dyads.
h1.d = hypothesis(m.pesi, "sync1:dyad.type1 < 0")
h1.d
```

```
## Hypothesis Tests for class b:
##                Hypothesis Estimate Est.Error CI.Lower CI.Upper Evid.Ratio
## 1 (sync1:dyad.type1) < 0    -0.06       0.4    -0.71      0.6       1.25
##   Post.Prob Star
## 1      0.56
## ---
## 'CI': 90%-CI for one-sided and 95%-CI for two-sided hypotheses.
## '*': For one-sided hypotheses, the posterior probability exceeds 95%;
```

```
## for two-sided hypotheses, the value tested against lies outside the 95%-CI.
## Posterior probabilities of point hypotheses assume equal prior probabilities.
```

```r
# H1.e Dyad type x diagnostic status: The effect of dyad type on ratings is
# decreased in autistic compared to comparison observers.
h1.e = hypothesis(m.pesi, "diagnosis1:dyad.type1 > 0")
h1.e
```

```
## Hypothesis Tests for class b:
##                 Hypothesis Estimate Est.Error CI.Lower CI.Upper Evid.Ratio
## 1 (diagnosis1:dyad.... > 0     0.24      0.39    -0.41     0.89       2.83
##   Post.Prob Star
## 1      0.74
## ---
## 'CI': 90%-CI for one-sided and 95%-CI for two-sided hypotheses.
## '*': For one-sided hypotheses, the posterior probability exceeds 95%;
## for two-sided hypotheses, the value tested against lies outside the 95%-CI.
## Posterior probabilities of point hypotheses assume equal prior probabilities.
```

```r
# Explore interaction between sync and dyad type in the COMP group only
e1 = hypothesis(m.pesi,
                "0 < diagnosis1:sync1:dyad.type1 - sync1:dyad.type1",
                alpha = 0.025)
e1
```

```
## Hypothesis Tests for class b:
##                 Hypothesis Estimate Est.Error CI.Lower CI.Upper Evid.Ratio
## 1 (0)-(diagnosis1:s... < 0    -0.53      0.52    -1.54     0.51       5.69
##   Post.Prob Star
## 1      0.85
## ---
## 'CI': 95%-CI for one-sided and 97.5%-CI for two-sided hypotheses.
## '*': For one-sided hypotheses, the posterior probability exceeds 97.5%;
## for two-sided hypotheses, the value tested against lies outside the 97.5%-CI.
## Posterior probabilities of point hypotheses assume equal prior probabilities.
```

```r
# extract predicted differences based on the model
df.new = df.agg %>%
  select(diagnosis, dyad.type, sync) %>%
  distinct() %>%
  mutate(
    condition = paste(diagnosis, dyad.type, sync, sep = "_")
  )
df.ms = as.data.frame(
  fitted(m.pesi, summary = F,
               newdata = df.new %>% select(diagnosis, dyad.type, sync),
               re_formula = NA))
colnames(df.ms) = df.new$condition

# compute specific differences
df.ms = df.ms %>%
  mutate(
    dyad.diff =
      rowMeans(select(., matches(".*_non-autistic_.*")), na.rm = T) -
      rowMeans(select(., matches(".*_mixed_.*")), na.rm = T),
    sync.diff =
```

Table 7: Summary Statistics

Variable	N	Mean	Std. Dev.	Min	Pctl. 25	Pctl. 75	Max
ASD_non-autistic_high	12000	57	3.5	41	55	60	73
ASD_non-autistic_low	12000	57	3.5	41	54	59	71
ASD_mixed_high	12000	51	3.4	35	49	53	65
ASD_mixed_low	12000	49	3.4	32	46	51	63
COMP_non-autistic_high	12000	57	3.3	42	55	59	72
COMP_non-autistic_low	12000	54	3.3	39	52	56	69
COMP_mixed_high	12000	47	3.2	34	45	50	61
COMP_mixed_low	12000	47	3.2	33	45	49	60
dyad.diff	12000	7.7	3.6	-8.1	5.5	9.9	29
sync.diff	12000	1.7	0.81	-1.3	1.1	2.2	4.4

```
      rowMeans(select(., ends_with("_high")), na.rm = T) -
      rowMeans(select(., ends_with("_low")), na.rm = T)
  )

# table for the cell values
st(df.ms, out = "kable")
```

The model revealed support for our hypotheses postulating decreased ratings for mixed dyads compared to non-autistic dyads (*estimate* = -3.87 [-6.79, -1.01], *posterior probability* = 0.984) and increased ratings for segments with high IPS as opposed to segments with low IPS (*estimate* = 0.83 [0.16, 1.49], *posterior probability* = 0.978). Specifically, the model predicts a mean difference of 7.742 [0.803, 14.907] between non-autistic and mixed dyads as well as a mean differences of 1.662 [0.039, 3.204] between high and low IPS. However, there is no support for our hypotheses regarding differences between the ratings of autistic and comparison observers (*estimate* = 1.07 [-1.61, 3.78], *posterior probability* = 0.786) or the interaction of IPS and dyad type (*estimate* = -0.06 [-0.71, 0.6], *posterior probability* = 0.555) or dyad type and diagnostic status of the observer (*estimate* = 0.24 [-0.41, 0.89], *posterior probability* = 0.739).

Plots

Finally, we can plot our data!

```
# rain cloud plot for ratings
df.agg %>%
  group_by(subID, diagnosis, dyad.type, sync) %>%
  summarise(
    rating.confirmed = mean(rating.confirmed, na.rm = T)
  ) %>%
  rename(`IPSmov` = sync) %>%
  mutate(
    observer = recode(diagnosis,
                      "ASD" = "autistic observer",
                      "COMP" = "comparison observer"),
    dyad.type = recode(dyad.type,
                      "mixed"        = "mixed dyad",
                      "non-autistic" = "non-autistic dyad")
  ) %>%
  ggplot(aes(dyad.type, rating.confirmed, fill = `IPSmov`, colour = `IPSmov`)) +
  geom_rain(rain.side = 'r',
```

```
            boxplot.args = list(colour = "black",
                                outlier.shape = NA,
                                show.legend = FALSE,
                                alpha = 0.75),
            violin.args  = list(colour = "black",
                                outlier.shape = NA,
                                show.legend = T,
                                alpha = .75),
            point.args   = list(show.legend = FALSE,
                                size  = .75,
                                alpha = .75),
            boxplot.args.pos = list(
              position =
                ggpp::position_dodgenudge(x = .1, width = 0.1),
              width = 0.1
            )) +
  ylim(0, 100) +
  facet_wrap(. ~ observer) +
  scale_fill_manual(values = c("#1E88E5", "#004D40")) +
  scale_color_manual(values = c("#1E88E5", "#004D40")) +
  labs(title = "Mean impression ratings",
       x = "",
       y = "rating") +
  theme_bw() +
  theme(legend.position = "bottom",
        plot.title = element_text(hjust = 0.5),
        legend.direction = "horizontal",
        text = element_text(size = 15))
```

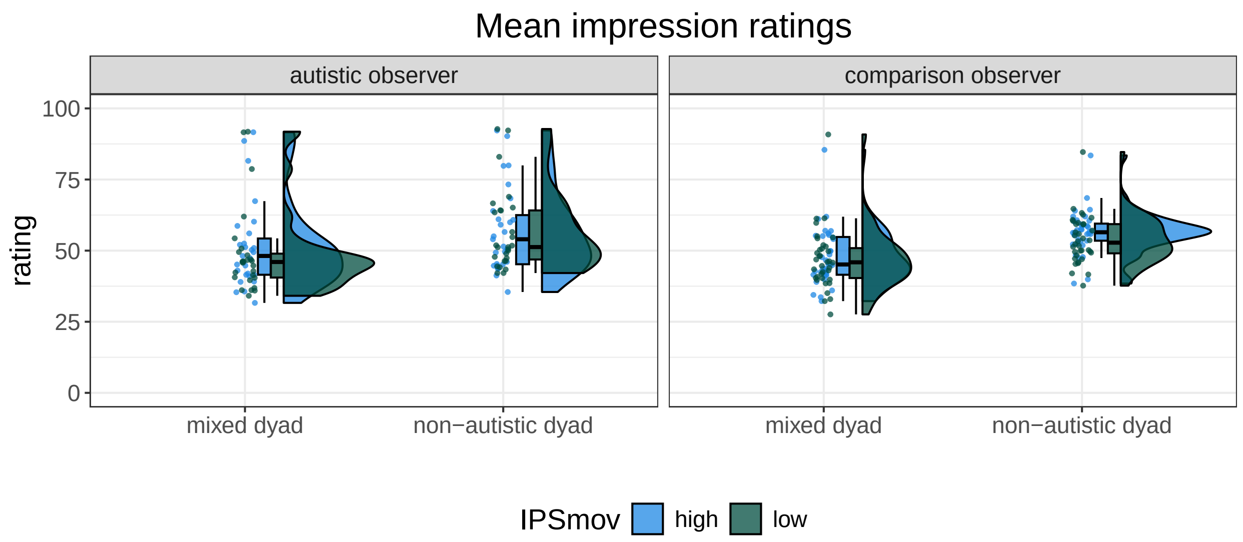


```
ggsave("Figure3_ratings.png",
       units = "mm",
       width  = 170,
       height = 100,
       dpi    = 300)
```

Fixation durations

We also collected eye tracking data during the presentation of the videos. From the eye tracking data, we extracted fixations using the algorithm from Nyström und Holmqvist (2010) and then calculated the duration of fixations on areas of interest (heads, hands and body). For testing the hypotheses, we calculated proportions of fixation durations from the total fixation duration for each video.

SBC

```
# set formula considering all combinations
code   = "PESI_fix"
f.fix = brms::bf(fix.prop ~ diagnosis * sync * dyad.type * AOI
                 + (sync * dyad.type * AOI | subID)
                 + (diagnosis * sync * AOI | dyad))

# set weakly informed priors
priors = c(
  # three AOIs, therefore, intercept expected to be around 33%
  prior(normal(33, 11), class = Intercept),
  prior(normal(11, 11), class = sigma), # 1/3 of the Intercept
  prior(normal(5,   5), class = sd),
  prior(lkj(2)        , class = cor),
  prior(normal(0,  10), class = b)
)

if (file.exists(file.path(cache_dir, paste0("df_res_", code, ".rds")))) {
  # load in the results of the SBC
  df.results = readRDS(file.path(cache_dir, paste0("df_res_", code, ".rds")))
  df.backend = readRDS(file.path(cache_dir, paste0("df_div_", code, ".rds")))
  dat        = readRDS(file.path(cache_dir, paste0("dat_", code, ".rds")))
} else {
  # set the seed
  set.seed(2469)
  # create the data
  gen   = SBC_generator_brms(f.fix, data = df.fix.agg, prior = priors,
                             thin = 50, warmup = 20000, refresh = 2000
  )
  dat   = generate_datasets(gen, nsim)
  saveRDS(dat, file = sprintf("%s/dat_%s.rds", cache_dir, code))

  # perform the SBC
  bck = SBC_backend_brms_from_generator(gen, chains = 4, thin = 1,
                                        warmup = warm, iter = iter,
                                        inits = 0.1)
  res = compute_SBC(dat, bck,
                    cache_mode = "results",
                    cache_location = file.path(cache_dir, sprintf("res_%s", code)))
  # save the results dataframes
  df.results = res$stats
  df.backend = res$backend_diagnostics
  saveRDS(df.results, file = file.path(cache_dir, paste0("df_res_", code, ".rds")))
  saveRDS(df.backend, file = file.path(cache_dir, paste0("df_div_", code, ".rds")))

}
```

Again, we start by investigating the rhats and the number of divergent samples. This shows that 8 of 500 simulations had at least one parameter that had an rhat of at least 1.05, and 17 models had divergent samples (mean number of samples of the simulations with divergent samples: 1.18). Although not perfect, we are satisfied with these numbers and continue.

Next, we can plot the simulated values to perform prior predictive checks.

```
# create a matrix out of generated data
dvname = gsub(" ", "", gsub("[\\|~].*", "", f.fix)[1])
dvfakemat = matrix(NA, nrow(dat[['generated']][[1]]), length(dat[['generated']]))
for (i in 1:length(dat[['generated']])) {
  dvfakemat[,i] = dat[['generated']][[i]][[dvname]]
}
truePars = dat$variables

# plot simulated data for prior predictive checks
#dvmax = 100
dvfakematH = dvfakemat;
#dvfakematH[dvfakematH > dvmax] = dvmax
#dvfakematH[dvfakematH < 0] = 0
breaks = seq(min(dvfakematH, na.rm = T)-1, max(dvfakematH, na.rm=T)+1, length.out = 100)
binwidth = ceiling(breaks[2] - breaks[1])
breaks = seq(min(dvfakematH, na.rm = T)-1, max(dvfakematH)+binwidth, by = binwidth)
histmat = matrix(NA, ncol = dim(dvfakematH)[2] + binwidth, nrow = length(breaks)-1)
for (i in 1:dim(dvfakematH)[2]) {
  histmat[,i] = hist(dvfakematH[,i], breaks = breaks, plot = F)$counts
}
probs = seq(0.1, 0.9, 0.1)
quantmat= as.data.frame(matrix(NA, nrow=dim(histmat)[1], ncol = length(probs)))
names(quantmat) = paste0("p", probs)
for (i in 1:dim(histmat)[1]) {
  quantmat[i,] = quantile(histmat[i,], p = probs, na.rm = T)
}
quantmat$x = breaks[2:length(breaks)] - binwidth/2 # add bin mean
ggplot(data = quantmat, aes(x = x)) +
  geom_ribbon(aes(ymax = p0.9, ymin = p0.1), fill = c_light) +
  geom_ribbon(aes(ymax = p0.8, ymin = p0.2), fill = c_light_highlight) +
  geom_ribbon(aes(ymax = p0.7, ymin = p0.3), fill = c_mid) +
  geom_ribbon(aes(ymax = p0.6, ymin = p0.4), fill = c_mid_highlight) +
  geom_line(aes(y = p0.5), colour = c_dark, linewidth = 1) +
  #xlim(0, max(dvfakematH)) +
  theme_bw()
```

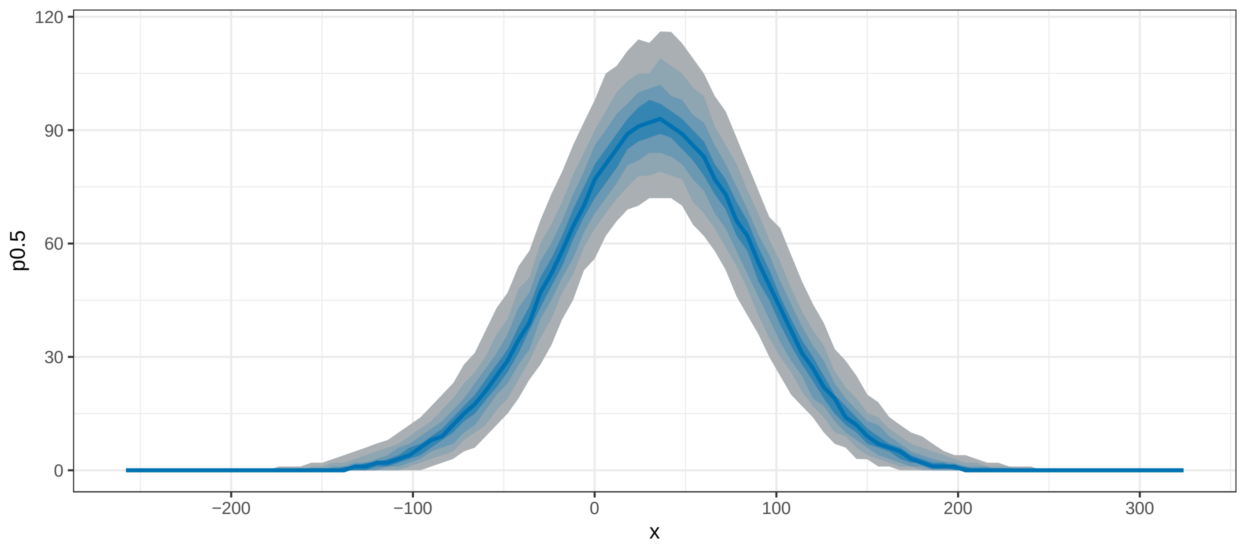

Again, most of our data fits our expectations, however, we chose wider priors, therefore, there are also simulated values outside of the possible values (0 to 100).

```
# get simulation numbers with issues
rank = max(df.results$max_rank)
check = merge(df.results %>%
    group_by(sim_id) %>%
      summarise(
        rhat = max(rhat, na.rm = T),
        mean_rank = mean(max_rank)
        ) %>%
    filter(rhat >= 1.05 | mean_rank != rank),
  df.backend %>% filter(n_divergent > 0), all = T)

# plot SBC with functions from the SBC package focusing on population-level parameters
df.results.b = df.results %>%
  filter(substr(variable, 1, 2) == "b_") %>%
  filter(!(sim_id %in% check$sim_id))
plot_ecdf_diff(df.results.b) + theme_bw() + theme(legend.position = "none") +
  scale_x_continuous(breaks=scales::pretty_breaks(n = 3)) +
  scale_y_continuous(breaks=scales::pretty_breaks(n = 3)) +
  ggtitle("Empirical cumulative distribution function")
```

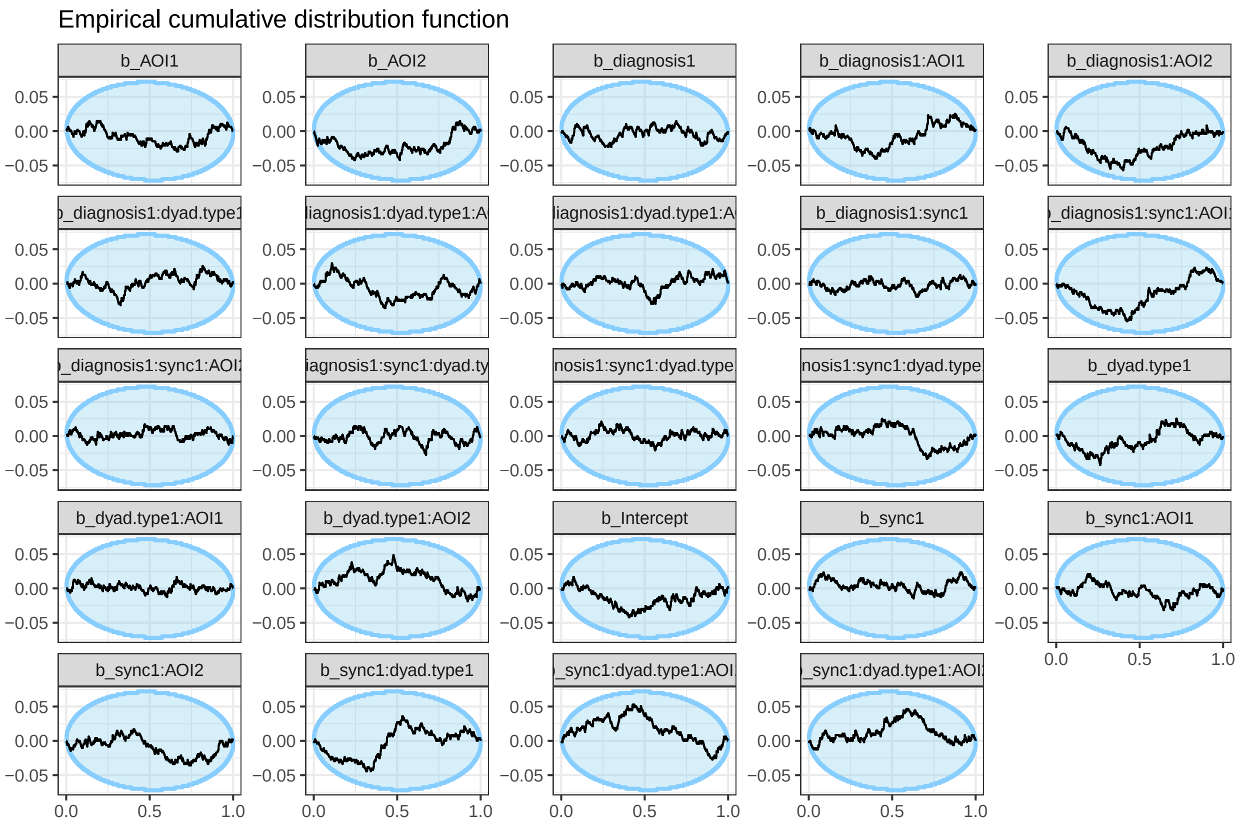


```r
plot_rank_hist(df.results.b, bins = 20) + theme_bw() +
  scale_x_continuous(breaks=scales::pretty_breaks(n = 3)) +
  scale_y_continuous(breaks=scales::pretty_breaks(n = 3)) +
  ggtitle("Rank histograms")
```

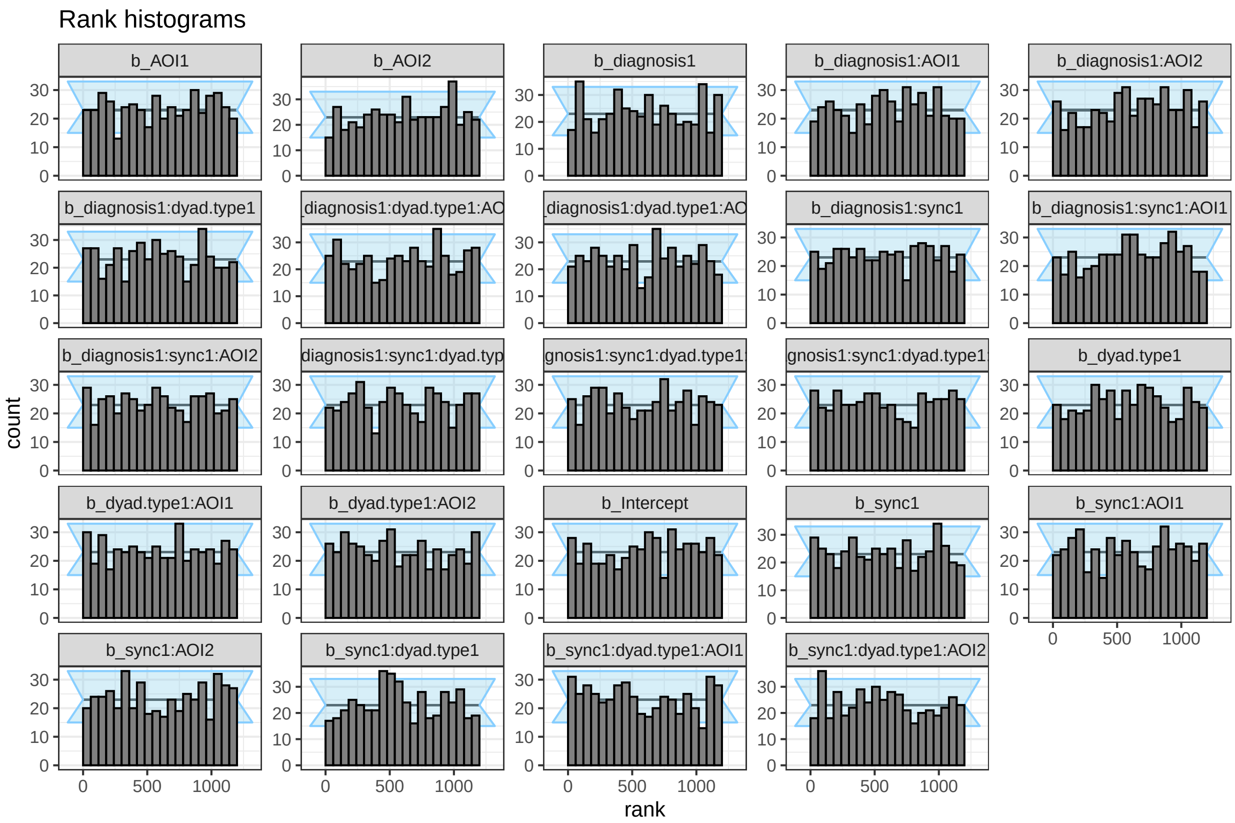


```r
plot_sim_estimated(df.results.b, alpha = 0.5) + theme_bw() +
  scale_x_continuous(breaks=scales::pretty_breaks(n = 3)) +
  scale_y_continuous(breaks=scales::pretty_breaks(n = 3)) +
  ggtitle("Simulated values")
```

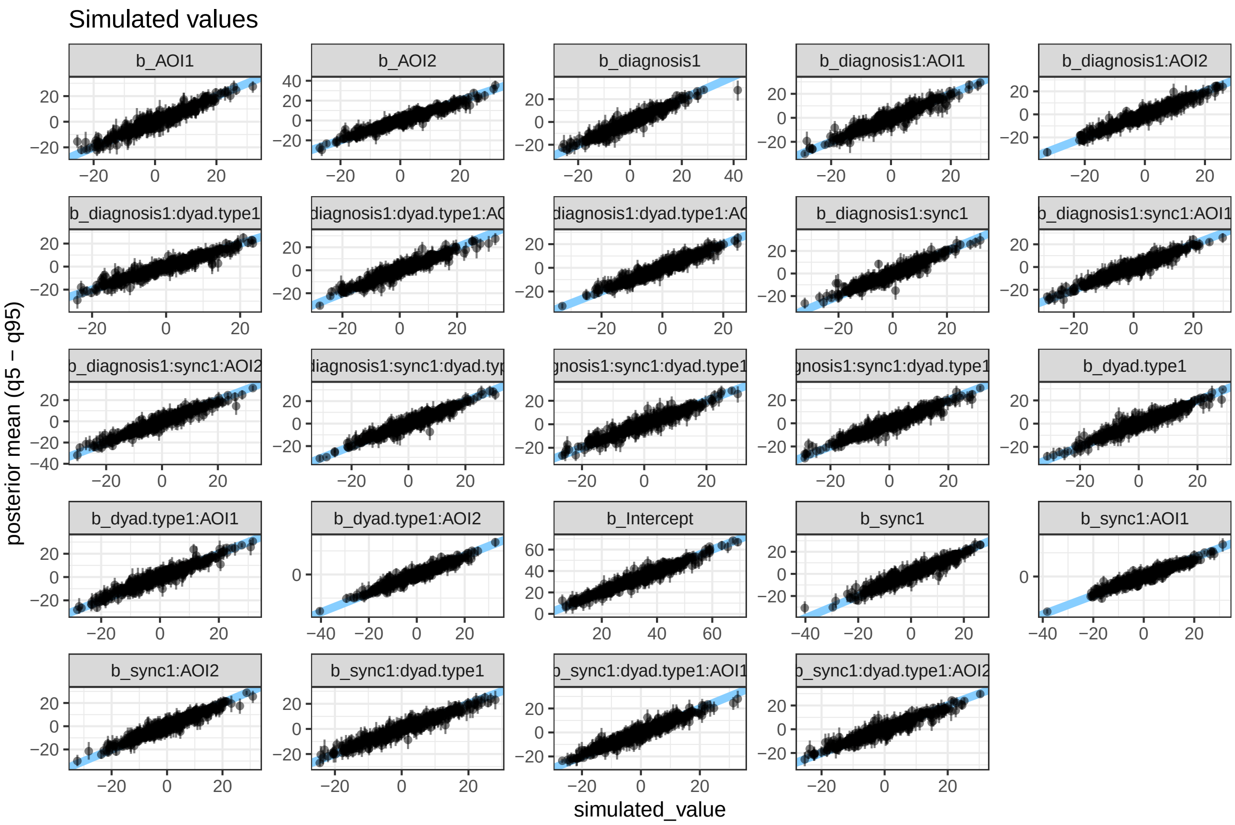


```r
plot_contraction(
  df.results.b,
  prior_sd = setNames(c(11,
                      rep(10, length(unique(df.results.b$variable))-1)),
                    unique(df.results.b$variable))) +
  theme_bw() +
  scale_x_continuous(breaks=scales::pretty_breaks(n = 3)) +
  scale_y_continuous(breaks=scales::pretty_breaks(n = 3)) +
  ggtitle("Posterior contraction and z-scores")
```

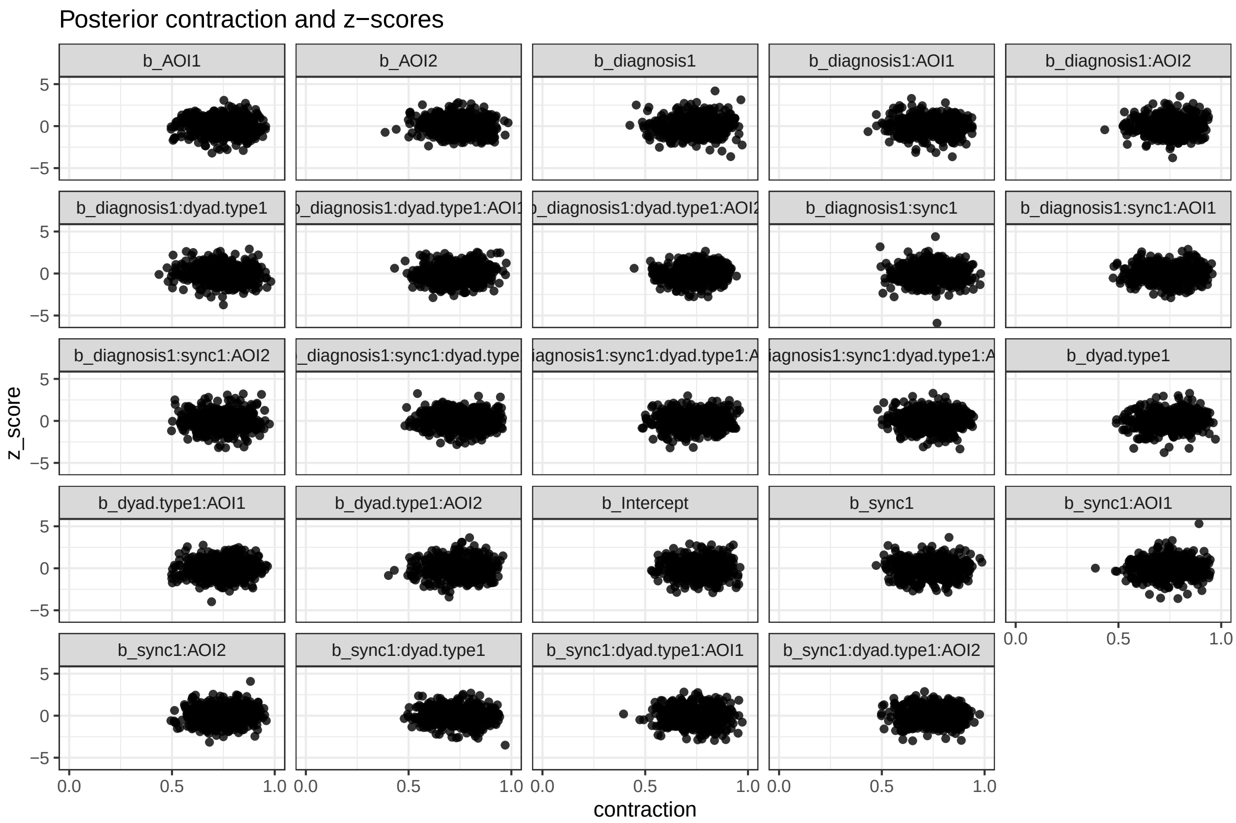

Second, we check the ranks of the parameters. If the model is unbiased, these should be uniformly distributed (Schad, Betancourt and Vasishth, 2020). The sample empirical cumulative distribution function (ECDF) lies within the theoretical distribution (95%) and the rank histogram also shows ranks within the 95% expected range, although there are some small deviations. We judge this to be acceptable.

Third, we investigated the relationship between the simulated true parameters and the posterior estimates. Although there are individual values diverging from the expected pattern, most parameters were recovered successfully within an uncertainty interval of alpha = 0.05.

Last, we explore the z-score and the posterior contraction of our population-level predictors. The z-score "determines the distance of the posterior mean from the true simulating parameter", while the posterior contraction "estimates how much prior uncertainty is reduced in the posterior estimation" (Schad, Betancourt and Vasisth, 2020). Both look acceptable.

Posterior predictive checks

As the next step, we fit the model and check for issues.

```
# fit the model
set.seed(2486)
m.fix = brm(f.fix,
            df.fix.agg, prior = priors,
            iter = iter, warmup = warm,
            backend = "cmdstanr", threads = threading(8),
            file = "m_PESI_fix"
            )

# in this model, there are no divergent samples
sum(subset(nuts_params(m.fix), Parameter == "divergent__")$Value)
```

```
## [1] 0
```

```r
# check that rhats are below 1.01
sum(brms::rhat(m.fix) >= 1.01, na.rm = T)
```

```
## [1] 0
```

```r
# and the chains have converged
post.draws = as_draws_df(m.fix)
mcmc_trace(post.draws, regex_pars = "^b_",
           facet_args = list(ncol = 2)) +
  scale_x_continuous(breaks=scales::pretty_breaks(n = 3)) +
  scale_y_continuous(breaks=scales::pretty_breaks(n = 3))
```

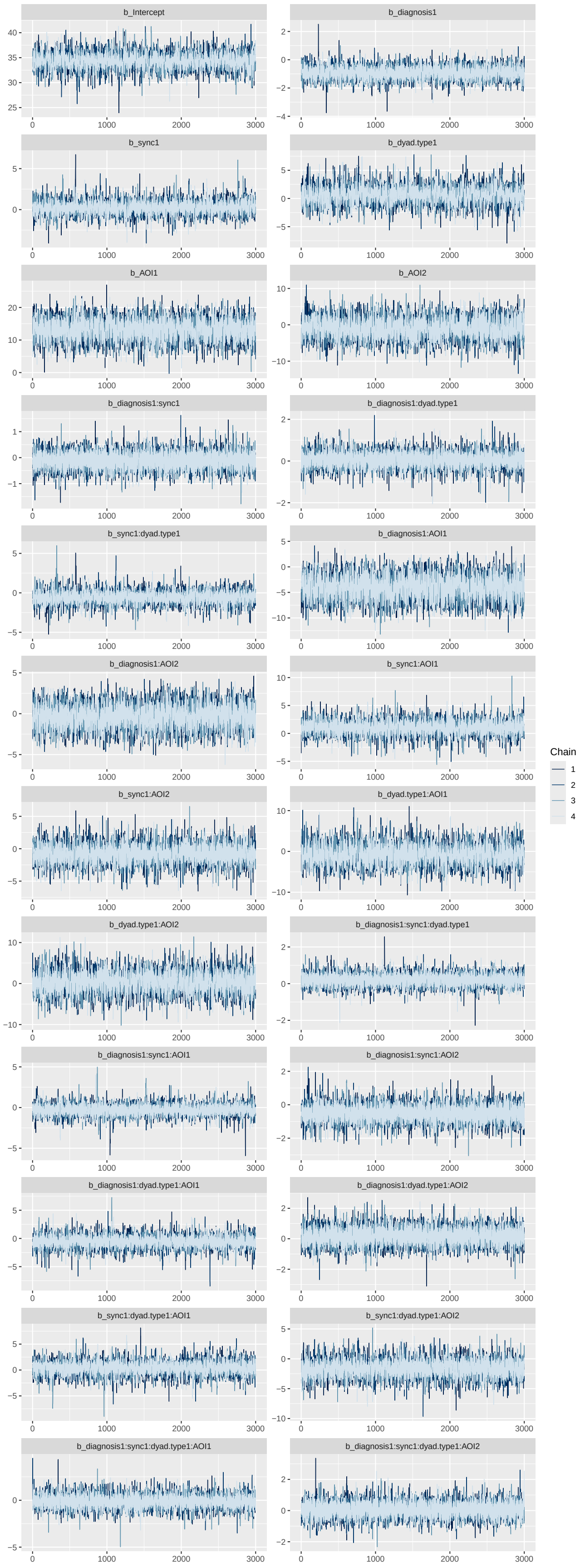
b_Intercept
b_diagnosis1
b_sync1
b_dyad.type1
b_AOI1
b_AOI2
b_diagnosis1:sync1
b_diagnosis1:dyad.type1
b_sync1:dyad.type1
b_diagnosis1:AOI1
b_diagnosis1:AOI2
b_sync1:AOI1
b_sync1:AOI2
b_dyad.type1:AOI1
b_dyad.type1:AOI2
b_diagnosis1:sync1:dyad.type1
b_diagnosis1:sync1:AOI1
b_diagnosis1:sync1:AOI2
b_diagnosis1:dyad.type1:AOI1
b_diagnosis1:dyad.type1:AOI2
b_sync1:dyad.type1:AOI1
b_sync1:dyad.type1:AOI2
b_diagnosis1:sync1:dyad.type1:AOI1
b_diagnosis1:sync1:dyad.type1:AOI2
Chain
1
2
3
4

This model has no divergent samples, and only one rhat that is higher or equal to 1.01. Therefore, we go ahead and perform our posterior predictive checks.

```
# get the posterior predictions
post.pred = posterior_predict(m.fix, ndraws = nsim)

# check the fit of the predicted data compared to the real data
p1 = pp_check(m.fix, ndraws = nsim) +
  theme_bw() + theme(legend.position = "none") + xlim(0,100)

# distributions of means and sds compared to the real values per group
p2 = ppc_stat_grouped(df.fix.agg$fix.prop, post.pred, df.fix.agg$diagnosis) +
  theme_bw() + theme(legend.position = "none")
# ... sync level
p3 = ppc_stat_grouped(df.fix.agg$fix.prop, post.pred, df.fix.agg$sync) +
  theme_bw() + theme(legend.position = "none")
# ... and dyad type
p4 = ppc_stat_grouped(df.fix.agg$fix.prop, post.pred, df.fix.agg$dyad.type) +
  theme_bw() + theme(legend.position = "none")

p = ggarrange(p1, p2, p3, p4,
          nrow = 2, ncol = 2, labels = "AUTO")
annotate_figure(p,
                top = text_grob("Posterior predictive checks",
                face = "bold", size = 14))
```

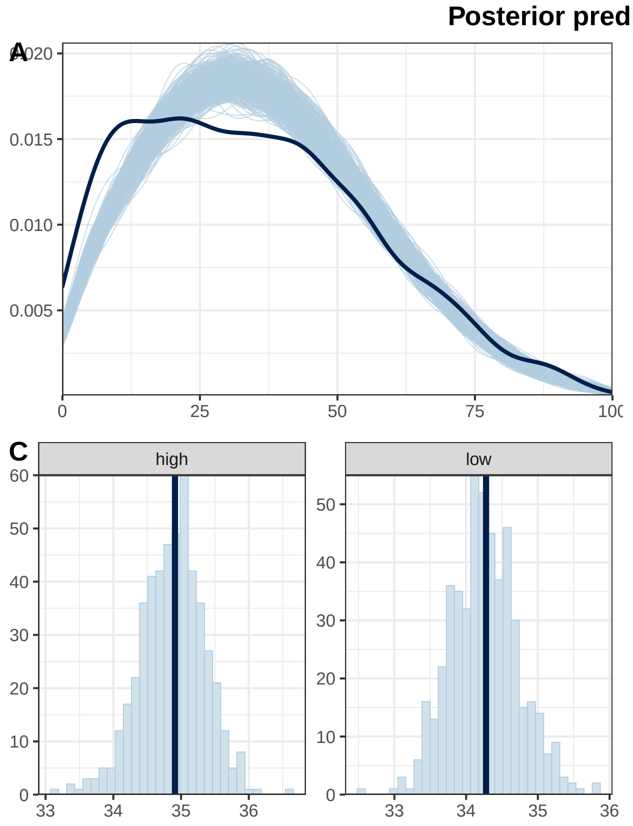

Although the overall shape is not perfect, the means are captured pretty well. Since we could not find a

likelihood shape that fit better than this, we accept this model and test our hypotheses.

Model summary

Now that we are convinced that we can trust our model, we have a look at the model and its estimates.

```r
# print a summary
summary(m.fix)
```

```
##  Family: gaussian
##   Links: mu = identity; sigma = identity
## Formula: fix.prop ~ diagnosis * sync * dyad.type * AOI + (sync * dyad.type * AOI | subID) + (diagnos
##    Data: df.fix.agg (Number of observations: 2160)
##   Draws: 4 chains, each with iter = 4500; warmup = 1500; thin = 1;
##          total post-warmup draws = 12000
##
## Multilevel Hyperparameters:
## ~dyad (Number of levels: 8)
##                                                    Estimate Est.Error l-95% CI
## sd(Intercept)                                          3.89      1.43     2.07
## sd(diagnosis1)                                         0.50      0.44     0.02
## sd(sync1)                                              1.81      0.85     0.78
## sd(AOI1)                                               6.30      1.95     3.47
## sd(AOI2)                                               6.56      1.83     3.84
## sd(diagnosis1:sync1)                                   0.37      0.34     0.01
## sd(diagnosis1:AOI1)                                    2.13      1.12     0.60
## sd(diagnosis1:AOI2)                                    0.58      0.54     0.02
## sd(sync1:AOI1)                                         2.77      1.28     1.07
## sd(sync1:AOI2)                                         3.43      1.26     1.74
## sd(diagnosis1:sync1:AOI1)                              1.12      0.84     0.06
## sd(diagnosis1:sync1:AOI2)                              0.67      0.57     0.03
## cor(Intercept,diagnosis1)                             -0.09      0.26    -0.57
## cor(Intercept,sync1)                                   0.20      0.24    -0.30
## cor(diagnosis1,sync1)                                 -0.07      0.25    -0.55
## cor(Intercept,AOI1)                                    0.03      0.23    -0.41
## cor(diagnosis1,AOI1)                                  -0.02      0.26    -0.51
## cor(sync1,AOI1)                                        0.08      0.23    -0.38
## cor(Intercept,AOI2)                                    0.21      0.23    -0.27
## cor(diagnosis1,AOI2)                                  -0.05      0.25    -0.53
## cor(sync1,AOI2)                                        0.15      0.23    -0.31
## cor(AOI1,AOI2)                                        -0.13      0.22    -0.55
## cor(Intercept,diagnosis1:sync1)                       -0.05      0.25    -0.53
## cor(diagnosis1,diagnosis1:sync1)                       0.01      0.26    -0.49
## cor(sync1,diagnosis1:sync1)                           -0.02      0.26    -0.51
## cor(AOI1,diagnosis1:sync1)                            -0.02      0.26    -0.51
## cor(AOI2,diagnosis1:sync1)                            -0.03      0.26    -0.52
## cor(Intercept,diagnosis1:AOI1)                         0.01      0.24    -0.45
## cor(diagnosis1,diagnosis1:AOI1)                       -0.02      0.26    -0.51
## cor(sync1,diagnosis1:AOI1)                             0.02      0.24    -0.46
## cor(AOI1,diagnosis1:AOI1)                             -0.04      0.24    -0.50
## cor(AOI2,diagnosis1:AOI1)                             -0.01      0.24    -0.46
## cor(diagnosis1:sync1,diagnosis1:AOI1)                  0.01      0.26    -0.49
## cor(Intercept,diagnosis1:AOI2)                        -0.03      0.26    -0.52
## cor(diagnosis1,diagnosis1:AOI2)                        0.01      0.26    -0.49
## cor(sync1,diagnosis1:AOI2)                            -0.02      0.26    -0.52
```

```
## cor(AOI1,diagnosis1:AOI2)                           0.03      0.26    -0.47
## cor(AOI2,diagnosis1:AOI2)                          -0.01      0.25    -0.50
## cor(diagnosis1:sync1,diagnosis1:AOI2)               0.01      0.26    -0.50
## cor(diagnosis1:AOI1,diagnosis1:AOI2)               -0.04      0.26    -0.54
## cor(Intercept,sync1:AOI1)                           0.04      0.24    -0.43
## cor(diagnosis1,sync1:AOI1)                          0.01      0.26    -0.49
## cor(sync1,sync1:AOI1)                               0.01      0.24    -0.45
## cor(AOI1,sync1:AOI1)                                0.10      0.24    -0.36
## cor(AOI2,sync1:AOI1)                                0.10      0.23    -0.36
## cor(diagnosis1:sync1,sync1:AOI1)                   -0.02      0.26    -0.52
## cor(diagnosis1:AOI1,sync1:AOI1)                    -0.12      0.24    -0.57
## cor(diagnosis1:AOI2,sync1:AOI1)                     0.03      0.26    -0.48
## cor(Intercept,sync1:AOI2)                           0.24      0.24    -0.24
## cor(diagnosis1,sync1:AOI2)                         -0.08      0.25    -0.56
## cor(sync1,sync1:AOI2)                               0.23      0.24    -0.27
## cor(AOI1,sync1:AOI2)                               -0.04      0.23    -0.48
## cor(AOI2,sync1:AOI2)                                0.24      0.23    -0.24
## cor(diagnosis1:sync1,sync1:AOI2)                   -0.03      0.26    -0.52
## cor(diagnosis1:AOI1,sync1:AOI2)                     0.05      0.24    -0.43
## cor(diagnosis1:AOI2,sync1:AOI2)                    -0.03      0.26    -0.52
## cor(sync1:AOI1,sync1:AOI2)                         -0.03      0.24    -0.48
## cor(Intercept,diagnosis1:sync1:AOI1)               -0.07      0.25    -0.55
## cor(diagnosis1,diagnosis1:sync1:AOI1)               0.01      0.26    -0.49
## cor(sync1,diagnosis1:sync1:AOI1)                   -0.08      0.25    -0.56
## cor(AOI1,diagnosis1:sync1:AOI1)                     0.03      0.25    -0.45
## cor(AOI2,diagnosis1:sync1:AOI1)                    -0.04      0.25    -0.52
## cor(diagnosis1:sync1,diagnosis1:sync1:AOI1)         0.01      0.26    -0.49
## cor(diagnosis1:AOI1,diagnosis1:sync1:AOI1)         -0.03      0.25    -0.52
## cor(diagnosis1:AOI2,diagnosis1:sync1:AOI1)          0.03      0.26    -0.46
## cor(sync1:AOI1,diagnosis1:sync1:AOI1)               0.02      0.25    -0.47
## cor(sync1:AOI2,diagnosis1:sync1:AOI1)              -0.09      0.25    -0.56
## cor(Intercept,diagnosis1:sync1:AOI2)               -0.04      0.26    -0.53
## cor(diagnosis1,diagnosis1:sync1:AOI2)               0.01      0.26    -0.49
## cor(sync1,diagnosis1:sync1:AOI2)                   -0.01      0.25    -0.50
## cor(AOI1,diagnosis1:sync1:AOI2)                     0.06      0.25    -0.44
## cor(AOI2,diagnosis1:sync1:AOI2)                    -0.03      0.25    -0.52
## cor(diagnosis1:sync1,diagnosis1:sync1:AOI2)         0.01      0.26    -0.50
## cor(diagnosis1:AOI1,diagnosis1:sync1:AOI2)         -0.06      0.26    -0.54
## cor(diagnosis1:AOI2,diagnosis1:sync1:AOI2)          0.02      0.26    -0.48
## cor(sync1:AOI1,diagnosis1:sync1:AOI2)               0.03      0.26    -0.47
## cor(sync1:AOI2,diagnosis1:sync1:AOI2)              -0.04      0.25    -0.53
## cor(diagnosis1:sync1:AOI1,diagnosis1:sync1:AOI2)    0.01      0.26    -0.49
##                                                  u-95% CI Rhat Bulk_ESS
## sd(Intercept)                                        7.52 1.00     4404
## sd(diagnosis1)                                       1.60 1.00     5271
## sd(sync1)                                            4.03 1.00     4836
## sd(AOI1)                                            10.89 1.00     6350
## sd(AOI2)                                            10.97 1.00     7056
## sd(diagnosis1:sync1)                                 1.21 1.00     6148
## sd(diagnosis1:AOI1)                                  5.01 1.00     4761
## sd(diagnosis1:AOI2)                                  1.92 1.00     6662
## sd(sync1:AOI1)                                       6.02 1.00     5908
## sd(sync1:AOI2)                                       6.62 1.00     6229
## sd(diagnosis1:sync1:AOI1)                            3.17 1.00     4174
```

```
## sd(diagnosis1:sync1:AOI2)                               2.10 1.00     5961
## cor(Intercept,diagnosis1)                               0.41 1.00    14429
## cor(Intercept,sync1)                                    0.63 1.00    10228
## cor(diagnosis1,sync1)                                   0.41 1.00     8366
## cor(Intercept,AOI1)                                     0.47 1.00    10877
## cor(diagnosis1,AOI1)                                    0.48 1.00     7566
## cor(sync1,AOI1)                                         0.52 1.00     9410
## cor(Intercept,AOI2)                                     0.63 1.00     9753
## cor(diagnosis1,AOI2)                                    0.45 1.00     6700
## cor(sync1,AOI2)                                         0.58 1.00     9361
## cor(AOI1,AOI2)                                          0.32 1.00     9609
## cor(Intercept,diagnosis1:sync1)                         0.45 1.00    15844
## cor(diagnosis1,diagnosis1:sync1)                        0.51 1.00    14277
## cor(sync1,diagnosis1:sync1)                             0.47 1.00    14291
## cor(AOI1,diagnosis1:sync1)                              0.48 1.00    11902
## cor(AOI2,diagnosis1:sync1)                              0.46 1.00    12985
## cor(Intercept,diagnosis1:AOI1)                          0.47 1.00    12327
## cor(diagnosis1,diagnosis1:AOI1)                         0.48 1.00     9180
## cor(sync1,diagnosis1:AOI1)                              0.48 1.00    11366
## cor(AOI1,diagnosis1:AOI1)                               0.42 1.00    10878
## cor(AOI2,diagnosis1:AOI1)                               0.45 1.00    11263
## cor(diagnosis1:sync1,diagnosis1:AOI1)                   0.51 1.00     8551
## cor(Intercept,diagnosis1:AOI2)                          0.47 1.00    17242
## cor(diagnosis1,diagnosis1:AOI2)                         0.51 1.00    13719
## cor(sync1,diagnosis1:AOI2)                              0.48 1.00    13312
## cor(AOI1,diagnosis1:AOI2)                               0.52 1.00    13081
## cor(AOI2,diagnosis1:AOI2)                               0.48 1.00    12920
## cor(diagnosis1:sync1,diagnosis1:AOI2)                   0.49 1.00     9506
## cor(diagnosis1:AOI1,diagnosis1:AOI2)                    0.47 1.00    10560
## cor(Intercept,sync1:AOI1)                               0.49 1.00    12623
## cor(diagnosis1,sync1:AOI1)                              0.51 1.00     8800
## cor(sync1,sync1:AOI1)                                   0.46 1.00    10370
## cor(AOI1,sync1:AOI1)                                    0.55 1.00    10456
## cor(AOI2,sync1:AOI1)                                    0.54 1.00     9959
## cor(diagnosis1:sync1,sync1:AOI1)                        0.49 1.00     7848
## cor(diagnosis1:AOI1,sync1:AOI1)                         0.36 1.00     9525
## cor(diagnosis1:AOI2,sync1:AOI1)                         0.52 1.00     8259
## cor(Intercept,sync1:AOI2)                               0.67 1.00    10603
## cor(diagnosis1,sync1:AOI2)                              0.42 1.00     8916
## cor(sync1,sync1:AOI2)                                   0.66 1.00    10350
## cor(AOI1,sync1:AOI2)                                    0.40 1.00    10599
## cor(AOI2,sync1:AOI2)                                    0.66 1.00     9760
## cor(diagnosis1:sync1,sync1:AOI2)                        0.47 1.00     9051
## cor(diagnosis1:AOI1,sync1:AOI2)                         0.51 1.00    10074
## cor(diagnosis1:AOI2,sync1:AOI2)                         0.47 1.00     8546
## cor(sync1:AOI1,sync1:AOI2)                              0.43 1.00     9922
## cor(Intercept,diagnosis1:sync1:AOI1)                    0.41 1.00    13107
## cor(diagnosis1,diagnosis1:sync1:AOI1)                   0.51 1.00    11759
## cor(sync1,diagnosis1:sync1:AOI1)                        0.41 1.00    11669
## cor(AOI1,diagnosis1:sync1:AOI1)                         0.51 1.00    10922
## cor(AOI2,diagnosis1:sync1:AOI1)                         0.44 1.00    12275
## cor(diagnosis1:sync1,diagnosis1:sync1:AOI1)             0.51 1.00     9506
## cor(diagnosis1:AOI1,diagnosis1:sync1:AOI1)              0.45 1.00    10376
## cor(diagnosis1:AOI2,diagnosis1:sync1:AOI1)              0.54 1.00     8899
```

```
## cor(sync1:AOI1,diagnosis1:sync1:AOI1)              0.50 1.00    10251
## cor(sync1:AOI2,diagnosis1:sync1:AOI1)              0.42 1.00     8609
## cor(Intercept,diagnosis1:sync1:AOI2)               0.47 1.00    14787
## cor(diagnosis1,diagnosis1:sync1:AOI2)              0.50 1.00    13784
## cor(sync1,diagnosis1:sync1:AOI2)                   0.47 1.00    13700
## cor(AOI1,diagnosis1:sync1:AOI2)                    0.53 1.00    12136
## cor(AOI2,diagnosis1:sync1:AOI2)                    0.46 1.00    11908
## cor(diagnosis1:sync1,diagnosis1:sync1:AOI2)        0.50 1.00     9924
## cor(diagnosis1:AOI1,diagnosis1:sync1:AOI2)         0.43 1.00    10475
## cor(diagnosis1:AOI2,diagnosis1:sync1:AOI2)         0.51 1.00     8863
## cor(sync1:AOI1,diagnosis1:sync1:AOI2)              0.53 1.00    10272
## cor(sync1:AOI2,diagnosis1:sync1:AOI2)              0.46 1.00     9674
## cor(diagnosis1:sync1:AOI1,diagnosis1:sync1:AOI2)   0.49 1.00     7670
##                                                Tail_ESS
## sd(Intercept)                                      6780
## sd(diagnosis1)                                     5419
## sd(sync1)                                          5957
## sd(AOI1)                                           8593
## sd(AOI2)                                           8102
## sd(diagnosis1:sync1)                               5660
## sd(diagnosis1:AOI1)                                4300
## sd(diagnosis1:AOI2)                                5771
## sd(sync1:AOI1)                                     6767
## sd(sync1:AOI2)                                     7612
## sd(diagnosis1:sync1:AOI1)                          4512
## sd(diagnosis1:sync1:AOI2)                          6806
## cor(Intercept,diagnosis1)                          9284
## cor(Intercept,sync1)                               8673
## cor(diagnosis1,sync1)                             10231
## cor(Intercept,AOI1)                                8879
## cor(diagnosis1,AOI1)                               8779
## cor(sync1,AOI1)                                    8886
## cor(Intercept,AOI2)                                9441
## cor(diagnosis1,AOI2)                               7441
## cor(sync1,AOI2)                                    8213
## cor(AOI1,AOI2)                                     9160
## cor(Intercept,diagnosis1:sync1)                    8155
## cor(diagnosis1,diagnosis1:sync1)                   9188
## cor(sync1,diagnosis1:sync1)                        9101
## cor(AOI1,diagnosis1:sync1)                         9278
## cor(AOI2,diagnosis1:sync1)                         8524
## cor(Intercept,diagnosis1:AOI1)                     9041
## cor(diagnosis1,diagnosis1:AOI1)                    9218
## cor(sync1,diagnosis1:AOI1)                         9474
## cor(AOI1,diagnosis1:AOI1)                          9242
## cor(AOI2,diagnosis1:AOI1)                         10172
## cor(diagnosis1:sync1,diagnosis1:AOI1)              9460
## cor(Intercept,diagnosis1:AOI2)                     9232
## cor(diagnosis1,diagnosis1:AOI2)                    8985
## cor(sync1,diagnosis1:AOI2)                         8941
## cor(AOI1,diagnosis1:AOI2)                          9660
## cor(AOI2,diagnosis1:AOI2)                          9434
## cor(diagnosis1:sync1,diagnosis1:AOI2)              9245
## cor(diagnosis1:AOI1,diagnosis1:AOI2)               9187
```

```
## cor(Intercept,sync1:AOI1)                             8682
## cor(diagnosis1,sync1:AOI1)                            8773
## cor(sync1,sync1:AOI1)                                10211
## cor(AOI1,sync1:AOI1)                                  9499
## cor(AOI2,sync1:AOI1)                                  9399
## cor(diagnosis1:sync1,sync1:AOI1)                      9088
## cor(diagnosis1:AOI1,sync1:AOI1)                       9650
## cor(diagnosis1:AOI2,sync1:AOI1)                       9577
## cor(Intercept,sync1:AOI2)                             9596
## cor(diagnosis1,sync1:AOI2)                            9146
## cor(sync1,sync1:AOI2)                                 9897
## cor(AOI1,sync1:AOI2)                                  9082
## cor(AOI2,sync1:AOI2)                                  9060
## cor(diagnosis1:sync1,sync1:AOI2)                      9546
## cor(diagnosis1:AOI1,sync1:AOI2)                       9877
## cor(diagnosis1:AOI2,sync1:AOI2)                      10241
## cor(sync1:AOI1,sync1:AOI2)                           10587
## cor(Intercept,diagnosis1:sync1:AOI1)                  8952
## cor(diagnosis1,diagnosis1:sync1:AOI1)                 9317
## cor(sync1,diagnosis1:sync1:AOI1)                      9442
## cor(AOI1,diagnosis1:sync1:AOI1)                       9516
## cor(AOI2,diagnosis1:sync1:AOI1)                       9218
## cor(diagnosis1:sync1,diagnosis1:sync1:AOI1)          10211
## cor(diagnosis1:AOI1,diagnosis1:sync1:AOI1)            9948
## cor(diagnosis1:AOI2,diagnosis1:sync1:AOI1)            9735
## cor(sync1:AOI1,diagnosis1:sync1:AOI1)                 9867
## cor(sync1:AOI2,diagnosis1:sync1:AOI1)                 9735
## cor(Intercept,diagnosis1:sync1:AOI2)                  9299
## cor(diagnosis1,diagnosis1:sync1:AOI2)                 9619
## cor(sync1,diagnosis1:sync1:AOI2)                     10093
## cor(AOI1,diagnosis1:sync1:AOI2)                       9925
## cor(AOI2,diagnosis1:sync1:AOI2)                       9518
## cor(diagnosis1:sync1,diagnosis1:sync1:AOI2)           9597
## cor(diagnosis1:AOI1,diagnosis1:sync1:AOI2)           10177
## cor(diagnosis1:AOI2,diagnosis1:sync1:AOI2)            9395
## cor(sync1:AOI1,diagnosis1:sync1:AOI2)                10490
## cor(sync1:AOI2,diagnosis1:sync1:AOI2)                10732
## cor(diagnosis1:sync1:AOI1,diagnosis1:sync1:AOI2)     10173
##
## ~subID (Number of levels: 45)
##                                                   Estimate Est.Error l-95% CI
## sd(Intercept)                                         1.71      0.33     1.10
## sd(sync1)                                             0.25      0.19     0.01
## sd(dyad.type1)                                        0.24      0.18     0.01
## sd(AOI1)                                             13.65      1.27    11.42
## sd(AOI2)                                              8.44      0.89     6.86
## sd(sync1:dyad.type1)                                  0.25      0.19     0.01
## sd(sync1:AOI1)                                        1.27      0.57     0.13
## sd(sync1:AOI2)                                        0.58      0.41     0.02
## sd(dyad.type1:AOI1)                                   2.22      0.49     1.25
## sd(dyad.type1:AOI2)                                   1.88      0.55     0.75
## sd(sync1:dyad.type1:AOI1)                             1.77      0.47     0.84
## sd(sync1:dyad.type1:AOI2)                             0.58      0.42     0.02
## cor(Intercept,sync1)                                  0.06      0.26    -0.45
```

```
## cor(Intercept,dyad.type1)                      0.01      0.26    -0.48
## cor(sync1,dyad.type1)                          0.01      0.26    -0.49
## cor(Intercept,AOI1)                            0.46      0.14     0.17
## cor(sync1,AOI1)                                0.06      0.26    -0.45
## cor(dyad.type1,AOI1)                           0.03      0.26    -0.48
## cor(Intercept,AOI2)                            0.17      0.16    -0.16
## cor(sync1,AOI2)                                0.00      0.26    -0.50
## cor(dyad.type1,AOI2)                          -0.00      0.26    -0.50
## cor(AOI1,AOI2)                                -0.54      0.10    -0.70
## cor(Intercept,sync1:dyad.type1)               -0.06      0.25    -0.54
## cor(sync1,sync1:dyad.type1)                    0.00      0.26    -0.49
## cor(dyad.type1,sync1:dyad.type1)              -0.00      0.26    -0.50
## cor(AOI1,sync1:dyad.type1)                    -0.03      0.25    -0.51
## cor(AOI2,sync1:dyad.type1)                    -0.03      0.26    -0.52
## cor(Intercept,sync1:AOI1)                      0.03      0.22    -0.41
## cor(sync1,sync1:AOI1)                          0.01      0.26    -0.49
## cor(dyad.type1,sync1:AOI1)                    -0.01      0.26    -0.50
## cor(AOI1,sync1:AOI1)                          -0.12      0.20    -0.49
## cor(AOI2,sync1:AOI1)                           0.02      0.20    -0.38
## cor(sync1:dyad.type1,sync1:AOI1)               0.02      0.26    -0.48
## cor(Intercept,sync1:AOI2)                      0.02      0.24    -0.46
## cor(sync1,sync1:AOI2)                          0.01      0.26    -0.49
## cor(dyad.type1,sync1:AOI2)                     0.01      0.26    -0.49
## cor(AOI1,sync1:AOI2)                           0.02      0.24    -0.44
## cor(AOI2,sync1:AOI2)                          -0.06      0.24    -0.51
## cor(sync1:dyad.type1,sync1:AOI2)               0.01      0.26    -0.49
## cor(sync1:AOI1,sync1:AOI2)                     0.02      0.26    -0.47
## cor(Intercept,dyad.type1:AOI1)                 0.10      0.19    -0.28
## cor(sync1,dyad.type1:AOI1)                     0.00      0.25    -0.49
## cor(dyad.type1,dyad.type1:AOI1)                0.03      0.26    -0.47
## cor(AOI1,dyad.type1:AOI1)                      0.13      0.16    -0.18
## cor(AOI2,dyad.type1:AOI1)                     -0.21      0.16    -0.52
## cor(sync1:dyad.type1,dyad.type1:AOI1)         -0.02      0.26    -0.52
## cor(sync1:AOI1,dyad.type1:AOI1)                0.02      0.23    -0.43
## cor(sync1:AOI2,dyad.type1:AOI1)                0.10      0.25    -0.41
## cor(Intercept,dyad.type1:AOI2)                -0.15      0.21    -0.54
## cor(sync1,dyad.type1:AOI2)                    -0.03      0.25    -0.52
## cor(dyad.type1,dyad.type1:AOI2)               -0.04      0.25    -0.52
## cor(AOI1,dyad.type1:AOI2)                     -0.22      0.17    -0.53
## cor(AOI2,dyad.type1:AOI2)                     -0.01      0.18    -0.36
## cor(sync1:dyad.type1,dyad.type1:AOI2)          0.01      0.25    -0.48
## cor(sync1:AOI1,dyad.type1:AOI2)               -0.04      0.23    -0.48
## cor(sync1:AOI2,dyad.type1:AOI2)               -0.09      0.25    -0.56
## cor(dyad.type1:AOI1,dyad.type1:AOI2)          -0.37      0.20    -0.71
## cor(Intercept,sync1:dyad.type1:AOI1)           0.05      0.20    -0.34
## cor(sync1,sync1:dyad.type1:AOI1)               0.00      0.26    -0.49
## cor(dyad.type1,sync1:dyad.type1:AOI1)          0.01      0.26    -0.48
## cor(AOI1,sync1:dyad.type1:AOI1)                0.18      0.17    -0.16
## cor(AOI2,sync1:dyad.type1:AOI1)               -0.03      0.18    -0.37
## cor(sync1:dyad.type1,sync1:dyad.type1:AOI1)    0.03      0.26    -0.47
## cor(sync1:AOI1,sync1:dyad.type1:AOI1)          0.08      0.23    -0.38
## cor(sync1:AOI2,sync1:dyad.type1:AOI1)         -0.01      0.25    -0.49
## cor(dyad.type1:AOI1,sync1:dyad.type1:AOI1)    -0.35      0.19    -0.69
## cor(dyad.type1:AOI2,sync1:dyad.type1:AOI1)    -0.05      0.22    -0.46
```

```
## cor(Intercept,sync1:dyad.type1:AOI2)              -0.00      0.25    -0.48
## cor(sync1,sync1:dyad.type1:AOI2)                   0.00      0.26    -0.50
## cor(dyad.type1,sync1:dyad.type1:AOI2)              0.00      0.26    -0.50
## cor(AOI1,sync1:dyad.type1:AOI2)                   -0.01      0.24    -0.47
## cor(AOI2,sync1:dyad.type1:AOI2)                    0.02      0.24    -0.45
## cor(sync1:dyad.type1,sync1:dyad.type1:AOI2)        0.00      0.26    -0.49
## cor(sync1:AOI1,sync1:dyad.type1:AOI2)              0.00      0.26    -0.49
## cor(sync1:AOI2,sync1:dyad.type1:AOI2)             -0.00      0.26    -0.49
## cor(dyad.type1:AOI1,sync1:dyad.type1:AOI2)         0.01      0.25    -0.48
## cor(dyad.type1:AOI2,sync1:dyad.type1:AOI2)        -0.08      0.25    -0.56
## cor(sync1:dyad.type1:AOI1,sync1:dyad.type1:AOI2)  -0.06      0.26    -0.53
##                                                  u-95% CI Rhat Bulk_ESS
## sd(Intercept)                                        2.38 1.00     7387
## sd(sync1)                                            0.70 1.00     7801
## sd(dyad.type1)                                       0.69 1.00     7318
## sd(AOI1)                                            16.37 1.00     5392
## sd(AOI2)                                            10.34 1.00     5614
## sd(sync1:dyad.type1)                                 0.70 1.00     7886
## sd(sync1:AOI1)                                       2.34 1.00     2800
## sd(sync1:AOI2)                                       1.50 1.00     4195
## sd(dyad.type1:AOI1)                                  3.21 1.00     5904
## sd(dyad.type1:AOI2)                                  2.95 1.00     3784
## sd(sync1:dyad.type1:AOI1)                            2.71 1.00     6288
## sd(sync1:dyad.type1:AOI2)                            1.56 1.00     5076
## cor(Intercept,sync1)                                 0.55 1.00    15421
## cor(Intercept,dyad.type1)                            0.51 1.00    18073
## cor(sync1,dyad.type1)                                0.51 1.00    14915
## cor(Intercept,AOI1)                                  0.70 1.00     3433
## cor(sync1,AOI1)                                      0.54 1.00     1508
## cor(dyad.type1,AOI1)                                 0.51 1.00     1686
## cor(Intercept,AOI2)                                  0.47 1.00     3794
## cor(sync1,AOI2)                                      0.50 1.00     1146
## cor(dyad.type1,AOI2)                                 0.51 1.00     1421
## cor(AOI1,AOI2)                                      -0.33 1.00     8458
## cor(Intercept,sync1:dyad.type1)                      0.44 1.00    16568
## cor(sync1,sync1:dyad.type1)                          0.50 1.00    13672
## cor(dyad.type1,sync1:dyad.type1)                     0.50 1.00    12629
## cor(AOI1,sync1:dyad.type1)                           0.46 1.00    16474
## cor(AOI2,sync1:dyad.type1)                           0.46 1.00    16616
## cor(Intercept,sync1:AOI1)                            0.45 1.00    10444
## cor(sync1,sync1:AOI1)                                0.50 1.00     6373
## cor(dyad.type1,sync1:AOI1)                           0.49 1.00     6815
## cor(AOI1,sync1:AOI1)                                 0.28 1.00    14166
## cor(AOI2,sync1:AOI1)                                 0.41 1.00    15116
## cor(sync1:dyad.type1,sync1:AOI1)                     0.52 1.00     6245
## cor(Intercept,sync1:AOI2)                            0.49 1.00    14880
## cor(sync1,sync1:AOI2)                                0.50 1.00    11676
## cor(dyad.type1,sync1:AOI2)                           0.51 1.00    10529
## cor(AOI1,sync1:AOI2)                                 0.48 1.00    15614
## cor(AOI2,sync1:AOI2)                                 0.42 1.00    15919
## cor(sync1:dyad.type1,sync1:AOI2)                     0.50 1.00     8796
## cor(sync1:AOI1,sync1:AOI2)                           0.51 1.00    11130
## cor(Intercept,dyad.type1:AOI1)                       0.46 1.00     6801
## cor(sync1,dyad.type1:AOI1)                           0.49 1.00     3825
```

```
## cor(dyad.type1,dyad.type1:AOI1)                      0.53 1.00     3504
## cor(AOI1,dyad.type1:AOI1)                            0.43 1.00    13141
## cor(AOI2,dyad.type1:AOI1)                            0.13 1.00    13554
## cor(sync1:dyad.type1,dyad.type1:AOI1)                0.48 1.00     4632
## cor(sync1:AOI1,dyad.type1:AOI1)                      0.46 1.00     6350
## cor(sync1:AOI2,dyad.type1:AOI1)                      0.56 1.00     5418
## cor(Intercept,dyad.type1:AOI2)                       0.27 1.00     7914
## cor(sync1,dyad.type1:AOI2)                           0.47 1.00     4640
## cor(dyad.type1,dyad.type1:AOI2)                      0.47 1.00     5032
## cor(AOI1,dyad.type1:AOI2)                            0.13 1.00    13980
## cor(AOI2,dyad.type1:AOI2)                            0.34 1.00    14379
## cor(sync1:dyad.type1,dyad.type1:AOI2)                0.49 1.00     6209
## cor(sync1:AOI1,dyad.type1:AOI2)                      0.42 1.00     7871
## cor(sync1:AOI2,dyad.type1:AOI2)                      0.41 1.00     6264
## cor(dyad.type1:AOI1,dyad.type1:AOI2)                 0.09 1.00     6612
## cor(Intercept,sync1:dyad.type1:AOI1)                 0.44 1.00     7736
## cor(sync1,sync1:dyad.type1:AOI1)                     0.50 1.00     4398
## cor(dyad.type1,sync1:dyad.type1:AOI1)                0.50 1.00     4281
## cor(AOI1,sync1:dyad.type1:AOI1)                      0.49 1.00    13562
## cor(AOI2,sync1:dyad.type1:AOI1)                      0.32 1.00    14172
## cor(sync1:dyad.type1,sync1:dyad.type1:AOI1)          0.52 1.00     5495
## cor(sync1:AOI1,sync1:dyad.type1:AOI1)                0.51 1.00     6736
## cor(sync1:AOI2,sync1:dyad.type1:AOI1)                0.47 1.00     6292
## cor(dyad.type1:AOI1,sync1:dyad.type1:AOI1)          0.06 1.00     9515
## cor(dyad.type1:AOI2,sync1:dyad.type1:AOI1)          0.38 1.00    10103
## cor(Intercept,sync1:dyad.type1:AOI2)                 0.47 1.00    14171
## cor(sync1,sync1:dyad.type1:AOI2)                     0.50 1.00    11966
## cor(dyad.type1,sync1:dyad.type1:AOI2)                0.49 1.00    12194
## cor(AOI1,sync1:dyad.type1:AOI2)                      0.46 1.00    16240
## cor(AOI2,sync1:dyad.type1:AOI2)                      0.49 1.00    15625
## cor(sync1:dyad.type1,sync1:dyad.type1:AOI2)          0.50 1.00     8759
## cor(sync1:AOI1,sync1:dyad.type1:AOI2)                0.49 1.00    11957
## cor(sync1:AOI2,sync1:dyad.type1:AOI2)                0.50 1.00     8648
## cor(dyad.type1:AOI1,sync1:dyad.type1:AOI2)          0.48 1.00    11564
## cor(dyad.type1:AOI2,sync1:dyad.type1:AOI2)          0.43 1.00    10115
## cor(sync1:dyad.type1:AOI1,sync1:dyad.type1:AOI2)     0.45 1.00    10391
##                                                   Tail_ESS
## sd(Intercept)                                         8199
## sd(sync1)                                             5464
## sd(dyad.type1)                                        5436
## sd(AOI1)                                              6940
## sd(AOI2)                                              8058
## sd(sync1:dyad.type1)                                  5974
## sd(sync1:AOI1)                                        2642
## sd(sync1:AOI2)                                        4184
## sd(dyad.type1:AOI1)                                   5951
## sd(dyad.type1:AOI2)                                   2867
## sd(sync1:dyad.type1:AOI1)                             5305
## sd(sync1:dyad.type1:AOI2)                             5717
## cor(Intercept,sync1)                                  9190
## cor(Intercept,dyad.type1)                             8830
## cor(sync1,dyad.type1)                                 8348
## cor(Intercept,AOI1)                                   5808
## cor(sync1,AOI1)                                       3325
```

```
## cor(dyad.type1,AOI1)                              3971
## cor(Intercept,AOI2)                               5940
## cor(sync1,AOI2)                                   2742
## cor(dyad.type1,AOI2)                              3162
## cor(AOI1,AOI2)                                    8166
## cor(Intercept,sync1:dyad.type1)                   9015
## cor(sync1,sync1:dyad.type1)                       8244
## cor(dyad.type1,sync1:dyad.type1)                  9069
## cor(AOI1,sync1:dyad.type1)                        8851
## cor(AOI2,sync1:dyad.type1)                        9833
## cor(Intercept,sync1:AOI1)                         8739
## cor(sync1,sync1:AOI1)                             7148
## cor(dyad.type1,sync1:AOI1)                        8804
## cor(AOI1,sync1:AOI1)                              8529
## cor(AOI2,sync1:AOI1)                              9012
## cor(sync1:dyad.type1,sync1:AOI1)                  8746
## cor(Intercept,sync1:AOI2)                         8898
## cor(sync1,sync1:AOI2)                             8935
## cor(dyad.type1,sync1:AOI2)                        9771
## cor(AOI1,sync1:AOI2)                              8190
## cor(AOI2,sync1:AOI2)                              9544
## cor(sync1:dyad.type1,sync1:AOI2)                  8499
## cor(sync1:AOI1,sync1:AOI2)                        9836
## cor(Intercept,dyad.type1:AOI1)                    8383
## cor(sync1,dyad.type1:AOI1)                        7125
## cor(dyad.type1,dyad.type1:AOI1)                   6701
## cor(AOI1,dyad.type1:AOI1)                        11216
## cor(AOI2,dyad.type1:AOI1)                        10128
## cor(sync1:dyad.type1,dyad.type1:AOI1)             7339
## cor(sync1:AOI1,dyad.type1:AOI1)                   7463
## cor(sync1:AOI2,dyad.type1:AOI1)                   8272
## cor(Intercept,dyad.type1:AOI2)                    8853
## cor(sync1,dyad.type1:AOI2)                        7758
## cor(dyad.type1,dyad.type1:AOI2)                   8036
## cor(AOI1,dyad.type1:AOI2)                        10100
## cor(AOI2,dyad.type1:AOI2)                         9803
## cor(sync1:dyad.type1,dyad.type1:AOI2)             7509
## cor(sync1:AOI1,dyad.type1:AOI2)                   9509
## cor(sync1:AOI2,dyad.type1:AOI2)                   7924
## cor(dyad.type1:AOI1,dyad.type1:AOI2)              5939
## cor(Intercept,sync1:dyad.type1:AOI1)              8793
## cor(sync1,sync1:dyad.type1:AOI1)                  7738
## cor(dyad.type1,sync1:dyad.type1:AOI1)             7642
## cor(AOI1,sync1:dyad.type1:AOI1)                  10178
## cor(AOI2,sync1:dyad.type1:AOI1)                  10142
## cor(sync1:dyad.type1,sync1:dyad.type1:AOI1)       8367
## cor(sync1:AOI1,sync1:dyad.type1:AOI1)             8178
## cor(sync1:AOI2,sync1:dyad.type1:AOI1)             9176
## cor(dyad.type1:AOI1,sync1:dyad.type1:AOI1)        9341
## cor(dyad.type1:AOI2,sync1:dyad.type1:AOI1)        9822
## cor(Intercept,sync1:dyad.type1:AOI2)              8993
## cor(sync1,sync1:dyad.type1:AOI2)                  9573
## cor(dyad.type1,sync1:dyad.type1:AOI2)             9659
## cor(AOI1,sync1:dyad.type1:AOI2)                   9399
```

```
## cor(AOI2,sync1:dyad.type1:AOI2)                      9339
## cor(sync1:dyad.type1,sync1:dyad.type1:AOI2)          9421
## cor(sync1:AOI1,sync1:dyad.type1:AOI2)                9947
## cor(sync1:AOI2,sync1:dyad.type1:AOI2)                9292
## cor(dyad.type1:AOI1,sync1:dyad.type1:AOI2)           9538
## cor(dyad.type1:AOI2,sync1:dyad.type1:AOI2)          11128
## cor(sync1:dyad.type1:AOI1,sync1:dyad.type1:AOI2)    10189
##
## Regression Coefficients:
##                                  Estimate Est.Error l-95% CI u-95% CI Rhat
## Intercept                           34.37      1.48    31.42    37.34 1.00
## diagnosis1                          -0.93      0.42    -1.74    -0.07 1.00
## sync1                                0.27      0.75    -1.23     1.77 1.00
## dyad.type1                           0.60      1.46    -2.34     3.62 1.00
## AOI1                                13.22      2.99     7.13    18.96 1.00
## AOI2                                -0.90      2.61    -6.04     4.27 1.00
## diagnosis1:sync1                    -0.11      0.31    -0.71     0.49 1.00
## diagnosis1:dyad.type1                0.06      0.34    -0.60     0.72 1.00
## sync1:dyad.type1                    -0.58      0.75    -2.07     0.91 1.00
## diagnosis1:AOI1                     -4.04      2.14    -8.32     0.15 1.00
## diagnosis1:AOI2                     -0.36      1.32    -3.00     2.26 1.00
## sync1:AOI1                           1.06      1.14    -1.27     3.27 1.00
## sync1:AOI2                          -0.80      1.32    -3.42     1.91 1.00
## dyad.type1:AOI1                     -0.72      2.28    -5.28     3.90 1.00
## dyad.type1:AOI2                      0.34      2.36    -4.36     5.04 1.00
## diagnosis1:sync1:dyad.type1          0.20      0.31    -0.40     0.80 1.00
## diagnosis1:sync1:AOI1               -0.27      0.63    -1.48     0.93 1.00
## diagnosis1:sync1:AOI2               -0.51      0.46    -1.44     0.42 1.00
## diagnosis1:dyad.type1:AOI1          -0.55      0.99    -2.53     1.43 1.00
## diagnosis1:dyad.type1:AOI2           0.17      0.53    -0.86     1.21 1.00
## sync1:dyad.type1:AOI1                0.40      1.17    -1.98     2.72 1.00
## sync1:dyad.type1:AOI2               -1.65      1.32    -4.37     1.03 1.00
## diagnosis1:sync1:dyad.type1:AOI1    -0.10      0.65    -1.37     1.19 1.00
## diagnosis1:sync1:dyad.type1:AOI2     0.06      0.47    -0.86     0.98 1.00
##                                  Bulk_ESS Tail_ESS
## Intercept                            4201     5609
## diagnosis1                           6612     7376
## sync1                                5370     6496
## dyad.type1                           3885     4918
## AOI1                                 5444     6640
## AOI2                                 5511     7240
## diagnosis1:sync1                    11530     8476
## diagnosis1:dyad.type1                8833     7039
## sync1:dyad.type1                     5806     6126
## diagnosis1:AOI1                      5403     6898
## diagnosis1:AOI2                      5924     7603
## sync1:AOI1                           6241     6234
## sync1:AOI2                           5855     6667
## dyad.type1:AOI1                      5249     6004
## dyad.type1:AOI2                      5777     6797
## diagnosis1:sync1:dyad.type1         11138     7961
## diagnosis1:sync1:AOI1                7610     6349
## diagnosis1:sync1:AOI2               10294     7285
## diagnosis1:dyad.type1:AOI1           6180     7398
```

```
## diagnosis1:dyad.type1:AOI2           9165     7949
## sync1:dyad.type1:AOI1                5815     6544
## sync1:dyad.type1:AOI2                6432     7120
## diagnosis1:sync1:dyad.type1:AOI1     8183     7881
## diagnosis1:sync1:dyad.type1:AOI2    10745     8234
##
## Further Distributional Parameters:
##       Estimate Est.Error l-95% CI u-95% CI Rhat Bulk_ESS Tail_ESS
## sigma    11.24      0.19    10.88    11.61 1.00    10563     8691
##
## Draws were sampled using sample(hmc). For each parameter, Bulk_ESS
## and Tail_ESS are effective sample size measures, and Rhat is the potential
## scale reduction factor on split chains (at convergence, Rhat = 1).
```

```r
# plot the posterior distributions
post.draws %>%
  select(starts_with("b_")) %>%
  pivot_longer(cols = starts_with("b_"),
               names_to = "coef",
               values_to = "estimate") %>%
  subset(!startsWith(coef, "b_Int")) %>%
  mutate(
    coef = substr(coef, 3, nchar(coef)),
    coef = str_replace_all(coef, ":", " x "),
    coef = str_replace_all(coef, "diagnosis1", "ASD"),
    coef = str_replace_all(coef, "sync1", "high sync"),
    coef = str_replace_all(coef, "dyad.type1", "mixed"),
    coef = str_replace_all(coef, "AOI1", "head"),
    coef = str_replace_all(coef, "AOI2", "body"),
    coef_order = case_when(
      coef == "ASD" ~ 100,
      coef == "high sync" ~ 99,
      coef == "mixed" ~ 98,
      coef == "head" ~ 97,
      coef == "body" ~ 96,
      T ~ 100-nchar(coef)),
    coef = fct_reorder(coef, coef_order)
  ) %>%
  group_by(coef) %>%
  mutate(
    cred = case_when(
      (mean(estimate) < 0 & quantile(estimate, probs = 0.975) < 0) |
        (mean(estimate) > 0 & quantile(estimate, probs = 0.025) > 0) ~ "credible",
      T ~ "not credible"
    )
  ) %>% ungroup() %>%
  ggplot(aes(x = estimate, y = coef, fill = cred)) +
  geom_vline(xintercept = 0, linetype = 'dashed') +
  ggdist::stat_halfeye(alpha = 0.7) + ylab(NULL) + theme_bw() +
  scale_fill_manual(values = c(credible = c_dark, c_light)) +
  theme(legend.position = "none")
```

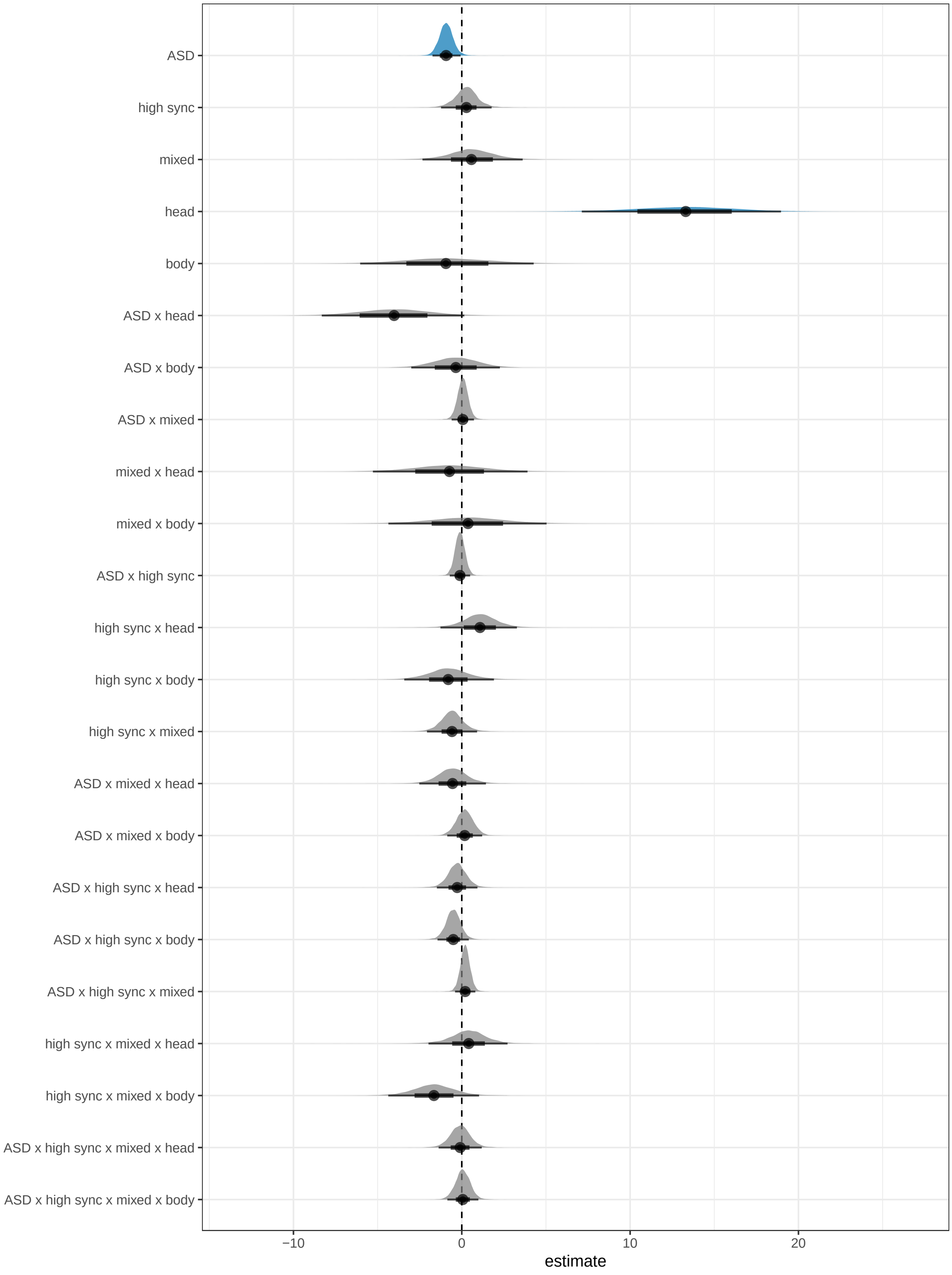
ASD
high sync
mixed
head
body
ASD x head
ASD x body
ASD x mixed
mixed x head
mixed x body
ASD x high sync
high sync x head
high sync x body
high sync x mixed
ASD x mixed x head
ASD x mixed x body
ASD x high sync x head
ASD x high sync x body
ASD x high sync x mixed
high sync x mixed x head
high sync x mixed x body
ASD x high sync x mixed x head
ASD x high sync x mixed x body
−10
0
10
20
estimate

Inferences

```
# H2.a Dyad type x AOI: Fixation durations for each area of interest differ
# between clips of no-diagnosis and mixed-diagnosis dyads.
h2.a = hypothesis(m.fix, "(dyad.type1:AOI1 + dyad.type1:AOI2)/2 < 0", alpha = 0.025)
h2.a
```

```
## Hypothesis Tests for class b:
##                 Hypothesis Estimate Est.Error CI.Lower CI.Upper Evid.Ratio
## 1 ((dyad.type1:AOI1... < 0    -0.19      1.52    -3.19     2.87       1.25
##   Post.Prob Star
## 1      0.55
## ---
## 'CI': 95%-CI for one-sided and 97.5%-CI for two-sided hypotheses.
## '*': For one-sided hypotheses, the posterior probability exceeds 97.5%;
## for two-sided hypotheses, the value tested against lies outside the 97.5%-CI.
## Posterior probabilities of point hypotheses assume equal prior probabilities.
```

```
# H2.b Synchrony x AOI: Fixation durations for each AOI differ between
# high- and low-synchrony clips.
h2.b = hypothesis(m.fix, "(sync1:AOI1 + sync1:AOI2)/2 > 0", alpha = 0.025)
h2.b
```

```
## Hypothesis Tests for class b:
##                 Hypothesis Estimate Est.Error CI.Lower CI.Upper Evid.Ratio
## 1 ((sync1:AOI1+sync... > 0     0.13      0.83    -1.55     1.85       1.32
##   Post.Prob Star
## 1      0.57
## ---
## 'CI': 95%-CI for one-sided and 97.5%-CI for two-sided hypotheses.
## '*': For one-sided hypotheses, the posterior probability exceeds 97.5%;
## for two-sided hypotheses, the value tested against lies outside the 97.5%-CI.
## Posterior probabilities of point hypotheses assume equal prior probabilities.
```

```
# H2.c Diagnostic status x AOI: Fixation durations for each AOI differ between
# autistic and comparison observers
h2.c = hypothesis(m.fix, "(diagnosis1:AOI1 + diagnosis1:AOI2)/2 < 0", alpha = 0.025)
h2.c
```

```
## Hypothesis Tests for class b:
##                 Hypothesis Estimate Est.Error CI.Lower CI.Upper Evid.Ratio
## 1 ((diagnosis1:AOI1... < 0     -2.2      0.97    -4.11    -0.31      76.42
##   Post.Prob Star
## 1      0.99    *
## ---
## 'CI': 95%-CI for one-sided and 97.5%-CI for two-sided hypotheses.
## '*': For one-sided hypotheses, the posterior probability exceeds 97.5%;
## for two-sided hypotheses, the value tested against lies outside the 97.5%-CI.
## Posterior probabilities of point hypotheses assume equal prior probabilities.
```

```
## exploring which AOI is driving this
# first, reminder of the contrast settings
contrasts(m.fix[["data"]][["AOI"]])
```

```
##      [,1] [,2]
## body   -1   -1
## hand    0    1
```

```
## head    1    0
```

```r
contrasts(m.fix[["data"]][["diagnosis"]])
```

```
##      [,1]
## ASD     1
## COMP   -1
```

```r
# ASD(body) > COMP(body)
e1.body = hypothesis(m.fix, "diagnosis1 - diagnosis1:AOI1 - diagnosis1:AOI2 > 0", alpha = 0.025)
e1.body
```

```
## Hypothesis Tests for class b:
##                 Hypothesis Estimate Est.Error CI.Lower CI.Upper Evid.Ratio
## 1 (diagnosis1-diagn... > 0     3.47      1.84    -0.17     7.14       31.7
##   Post.Prob Star
## 1      0.97
## ---
## 'CI': 95%-CI for one-sided and 97.5%-CI for two-sided hypotheses.
## '*': For one-sided hypotheses, the posterior probability exceeds 97.5%;
## for two-sided hypotheses, the value tested against lies outside the 97.5%-CI.
## Posterior probabilities of point hypotheses assume equal prior probabilities.
```

```r
# ASD(hand) < COMP(hand)
e2.hand = hypothesis(m.fix, "-diagnosis1 - diagnosis1:AOI2 > 0", alpha = 0.025)
e2.hand
```

```
## Hypothesis Tests for class b:
##                 Hypothesis Estimate Est.Error CI.Lower CI.Upper Evid.Ratio
## 1 (-diagnosis1-diag... > 0     1.28      1.44    -1.56     4.12       4.47
##   Post.Prob Star
## 1      0.82
## ---
## 'CI': 95%-CI for one-sided and 97.5%-CI for two-sided hypotheses.
## '*': For one-sided hypotheses, the posterior probability exceeds 97.5%;
## for two-sided hypotheses, the value tested against lies outside the 97.5%-CI.
## Posterior probabilities of point hypotheses assume equal prior probabilities.
```

```r
# ASD(head) < COMP(head)
e3.head = hypothesis(m.fix, "-diagnosis1 -diagnosis1:AOI1 > 0", alpha = 0.025)
e3.head
```

```
## Hypothesis Tests for class b:
##                 Hypothesis Estimate Est.Error CI.Lower CI.Upper Evid.Ratio
## 1 (-diagnosis1-diag... > 0     4.97      2.27     0.53      9.5      70.43
##   Post.Prob Star
## 1      0.99    *
## ---
## 'CI': 95%-CI for one-sided and 97.5%-CI for two-sided hypotheses.
## '*': For one-sided hypotheses, the posterior probability exceeds 97.5%;
## for two-sided hypotheses, the value tested against lies outside the 97.5%-CI.
## Posterior probabilities of point hypotheses assume equal prior probabilities.
```

```r
# extract predicted differences based on the model
df.new = df.fix %>%
  select(diagnosis, dyad.type, sync, AOI) %>%
  distinct() %>%
  mutate(
```

Table 8: Summary Statistics

Variable	N	Mean	Std. Dev.	Min	Pctl. 25	Pctl. 75	Max
ASD.body	12000	26	4.1	8.2	23	28	47
ASD.hand	12000	32	3.5	18	30	34	50
ASD.head	12000	43	4.3	27	40	45	59
COMP.body	12000	19	3.9	0.37	16	21	41
COMP.hand	12000	35	3.4	18	33	37	53
COMP.head	12000	53	4	34	50	55	69
body.diff	12000	-6.9	3.7	-24	-9.3	-4.6	9.9
hand.diff	12000	2.6	2.9	-8.1	0.66	4.5	14
head.diff	12000	9.9	4.5	-8.7	6.9	13	28

```
    condition = paste(diagnosis, dyad.type, sync, AOI, sep = "_")
  )
df.ms = as.data.frame(
  fitted(m.fix, summary = F,
              newdata = df.new %>% select(diagnosis, dyad.type, sync, AOI),
              re_formula = NA))
colnames(df.ms) = df.new$condition

# compute specific differences
df.ms = df.ms %>%
  mutate(
    ASD.body  = rowMeans(select(., matches("ASD.*_body")), na.rm = T),
    ASD.hand  = rowMeans(select(., matches("ASD.*_hand")), na.rm = T),
    ASD.head  = rowMeans(select(., matches("ASD.*_head")), na.rm = T),
    COMP.body = rowMeans(select(., matches("COMP.*_body")), na.rm = T),
    COMP.hand = rowMeans(select(., matches("COMP.*_hand")), na.rm = T),
    COMP.head = rowMeans(select(., matches("COMP.*_head")), na.rm = T),
    body.diff = COMP.body - ASD.body,
    hand.diff = COMP.hand - ASD.hand,
    head.diff = COMP.head - ASD.head,
    .keep = "none"
  )

# table for the cell values
st(df.ms, out = "kable")
```

This model revealed support for our expectation of differences between autistic and comparison observers dependent on the specific AOI (*estimate* = -2.2 [-4.11, -0.31], *posterior probability* = 0.987). Further exploration shows that this is driven by decreased dwell times on the head region for autistic observers (*estimate* = 4.97 [0.53, 9.5], *posterior probability* = 0.986). Specifically, this model predicts a difference of dwell times on the heads of the interaction partners of 9.945% [1.055, 18.995] between autistic and comparison observers. There is no support for our hypotheses regarding interactions between IPS and AOI (*estimate* = 0.13 [-1.55, 1.85], *posterior probability* = 0.57) or dyad type and AOI (*estimate* = -0.19 [-3.19, 2.87], *posterior probability* = 0.555).

Plots

At the end, we again plot our data.

```r
# rain cloud plot for fixation durations
df.fix.agg %>%
  group_by(subID, diagnosis, sync, dyad.type, AOI) %>%
  summarise(
    fix.prop = mean(fix.prop, na.rm = T)
  ) %>%
  mutate(
    observer = recode(diagnosis,
                      "ASD" = "autistic",
                      "COMP" = "comparison"),
    `IPSmov` = if_else(
      sync == "high", "high IPSmov","low IPSmov"
    ),
    dyad.type = recode(dyad.type,
                      "mixed"        = "mixed dyad",
                      "non-autistic" = "non-autistic dyad")
  ) %>%
  ggplot(aes(AOI, fix.prop, fill = observer, colour = observer)) +
  geom_rain(rain.side = 'r',
            boxplot.args = list(colour = "black",
                                outlier.shape = NA,
                                show.legend = FALSE,
                                alpha = .75),
            violin.args  = list(colour = "black",
                                outlier.shape = NA,
                                show.legend = T,
                                alpha = .75),
            point.args   = list(show.legend = FALSE,
                                size  = 0.75,
                                alpha = .75),
            boxplot.args.pos = list(
              position =
                ggpp::position_dodgenudge(x = .1, width = 0.1),
              width = 0.1
            )) +
  ylim(0, 100) +
  facet_wrap(dyad.type ~ `IPSmov`) +
  scale_fill_manual(values = c("#CC79A7", "#D55E00")) +
  scale_color_manual(values = c("#CC79A7", "#D55E00")) +
  labs(title = "Mean dwell times",
       x = "",
       y = "percent") +
  theme_bw() +
  theme(legend.position = "bottom",
        plot.title = element_text(hjust = 0.5),
        legend.direction = "horizontal",
        text = element_text(size = 15))
```

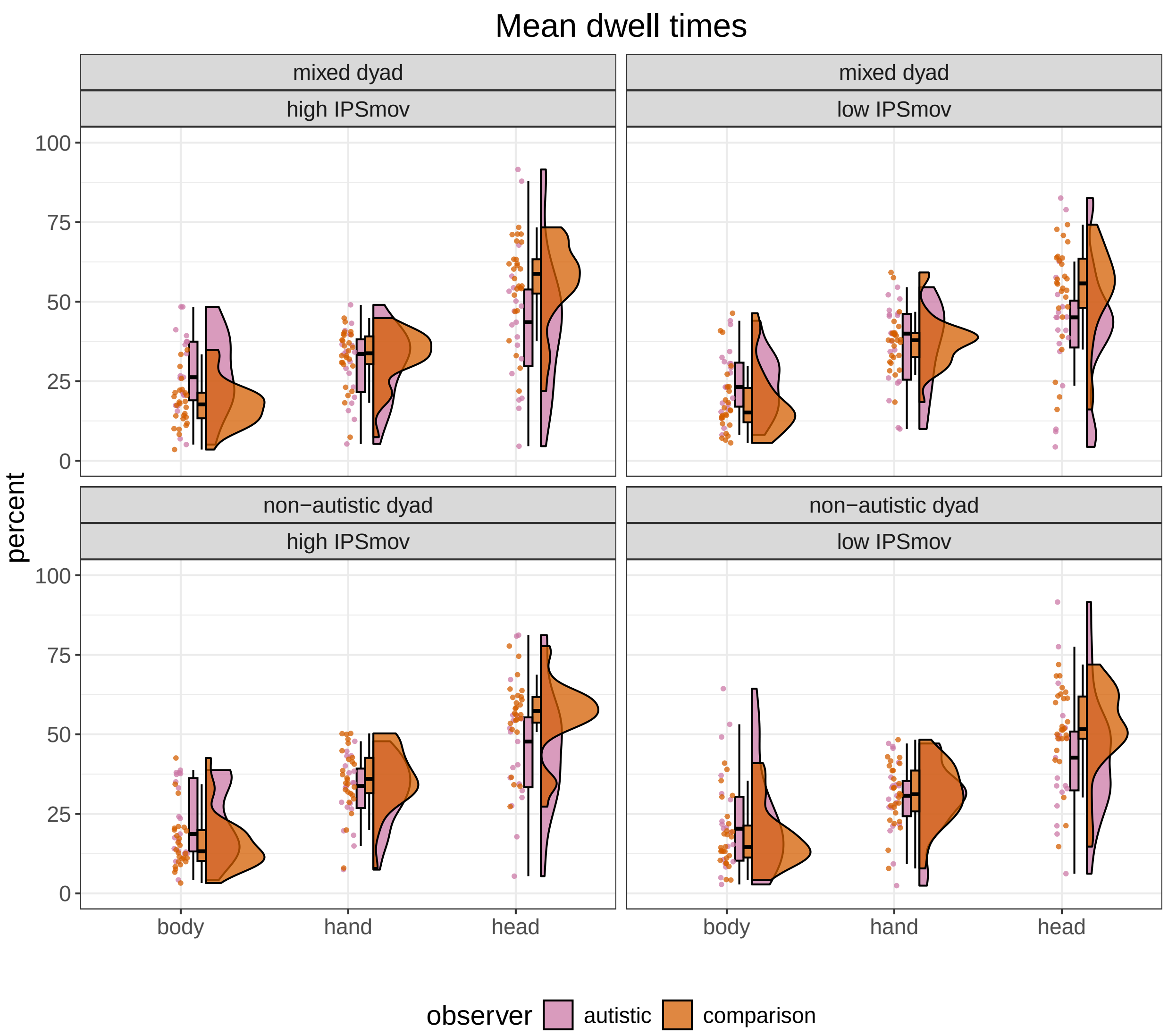


```r
ggsave("Figure4_dwelltimes.png",
       units = "mm",
       width  = 170,
       height = 170,
       dpi    = 300)
```

Explorative analyses

We complement our hypotheses-guided analyses with some exploration of the data.

Number of saccades

First, we use the saccades extracted by the Nyström and Holmqvist (2010) algorithm to explore whether there are differences in saccadic behaviour between our groups or the conditions.

SBC

```r
# set formula considering all combinations
code   = "PESI_sac_int"
f.sac = brms::bf(n.sac ~ diagnosis * sync * dyad.type
                 + (1 | subID)
                 + (1 | dyad))

# set weakly informed priors
priors = c(
  # one saccade per second
  prior(normal(2.3, 1.00), class = Intercept),
  prior(normal(0,   0.20), class = sd),
  prior(normal(0,   0.10), class = b)
)

if (file.exists(file.path(cache_dir, paste0("df_res_", code, ".rds")))) {
  # load in the results of the SBC
  df.results = readRDS(file.path(cache_dir, paste0("df_res_", code, ".rds")))
  df.backend = readRDS(file.path(cache_dir, paste0("df_div_", code, ".rds")))
  dat        = readRDS(file.path(cache_dir, paste0("dat_", code, ".rds")))
} else {
  # set the seed
  set.seed(2489)
  # create the data
  gen  = SBC_generator_brms(f.sac, data = df.sac.agg, prior = priors,
                            thin = 50, warmup = 20000, refresh = 2000,
                            family = poisson
  )
  if (!file.exists(sprintf("%s/dat_%s.rds", cache_dir, code))) {
    dat  = generate_datasets(gen, nsim)
    saveRDS(dat, file = sprintf("%s/dat_%s.rds", cache_dir, code))
  } else {
    dat = readRDS(sprintf("%s/dat_%s.rds", cache_dir, code))
  }

  # perform the SBC
  bck = SBC_backend_brms_from_generator(gen, chains = 4, thin = 1,
                                        warmup = warm, iter = iter,
                                        inits = 0.1)
  res = compute_SBC(dat, bck,
                    cache_mode = "results",
                    cache_location = file.path(cache_dir, sprintf("res_%s", code)))
  # save the results dataframes
  df.results = res$stats
  df.backend = res$backend_diagnostics
  saveRDS(df.results, file = file.path(cache_dir, paste0("df_res_", code, ".rds")))
  saveRDS(df.backend, file = file.path(cache_dir, paste0("df_div_", code, ".rds")))

}
```

Again, we start by investigating the rhats and the number of divergent samples. This shows that 0 of 500 simulations had at least one parameter that had an rhat of at least 1.05, and 162 models had divergent samples (mean number of samples of the simulations with divergent samples: 7.54). We will have to look out

for divergence issues in the final model.

Next, we can plot the simulated values to perform prior predictive checks.

```
# create a matrix out of generated data
dvname = gsub(" ", "", gsub("[\\|~].*", "", f.sac)[1])
dvfakemat = matrix(NA, nrow(dat[['generated']][[1]]), length(dat[['generated']]))
for (i in 1:length(dat[['generated']])) {
  dvfakemat[,i] = dat[['generated']][[i]][[dvname]]
}
truePars = dat$variables

# set very large data points to a value of 200
dvfakematH = dvfakemat;
dvfakematH[dvfakematH > 100] = 100
# compute one histogram per simulated data-set
breaks = seq(0, max(dvfakematH, na.rm=T), length.out = 100)
binwidth = breaks[2] - breaks[1]
histmat = matrix(NA, ncol = nrow(truePars) + binwidth, nrow = length(breaks)-1)
for (i in 1:nrow(truePars)) {
  histmat[,i] = hist(dvfakematH[,i], breaks = breaks, plot = F)$counts
}
# for each bin, compute quantiles across histograms
probs = seq(0.1, 0.9, 0.1)
quantmat= as.data.frame(matrix(NA, nrow=dim(histmat)[1], ncol = length(probs)))
names(quantmat) = paste0("p", probs)
for (i in 1:dim(histmat)[1]) {
  quantmat[i,] = quantile(histmat[i,], p = probs, na.rm = T)
}
quantmat$x = breaks[2:length(breaks)] - binwidth/2 # add bin mean
p1 = ggplot(data = quantmat, aes(x = x)) +
  geom_ribbon(aes(ymax = p0.9, ymin = p0.1), fill = c_light) +
  geom_ribbon(aes(ymax = p0.8, ymin = p0.2), fill = c_light_highlight) +
  geom_ribbon(aes(ymax = p0.7, ymin = p0.3), fill = c_mid) +
  geom_ribbon(aes(ymax = p0.6, ymin = p0.4), fill = c_mid_highlight) +
  geom_line(aes(y = p0.5), colour = c_dark, linewidth = 1) +
  labs(title = "Prior predictive distribution", y = "", x = "number of saccades") +
  theme_bw()
p1
```

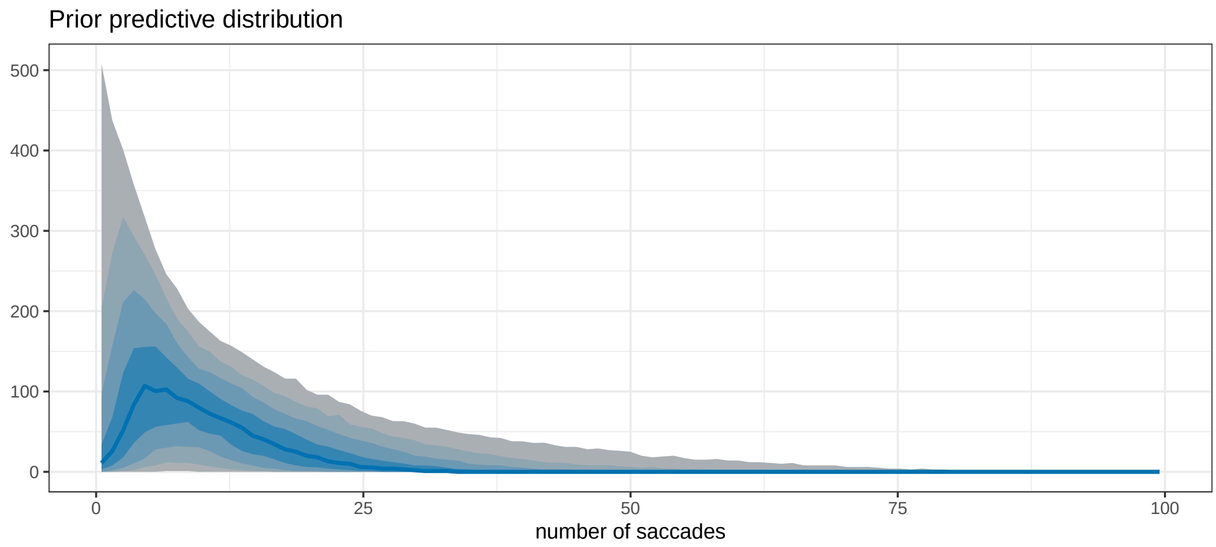

Our simulated data centres around observers only producing a couple of saccades per video, which is reasonable since the videos are only 10 seconds long. However, there is some data simulated that shows up to 50 saccades, so it is reasonably wide and we are happy with the prior predictive distribution.

```
# get simulation numbers with issues
rank = max(df.results$max_rank)
check = merge(df.results %>%
    group_by(sim_id) %>%
      summarise(
        rhat = max(rhat, na.rm = T),
        mean_rank = mean(max_rank)
        ) %>%
    filter(rhat >= 1.05 | mean_rank != rank),
  df.backend %>% filter(n_divergent > 0), all = T)

# plot SBC with functions from the SBC package focusing on population-level parameters
df.results.b = df.results %>%
  filter(substr(variable, 1, 2) == "b_") %>%
  filter(!(sim_id %in% check$sim_id))
p1 = plot_ecdf_diff(df.results.b) + theme_bw() + theme(legend.position = "none") +
  scale_x_continuous(breaks=scales::pretty_breaks(n = 3)) +
  scale_y_continuous(breaks=scales::pretty_breaks(n = 3))
p2 = plot_rank_hist(df.results.b, bins = 20) + theme_bw() +
  scale_x_continuous(breaks=scales::pretty_breaks(n = 3)) +
  scale_y_continuous(breaks=scales::pretty_breaks(n = 3))
p3 = plot_sim_estimated(df.results.b, alpha = 0.5) + theme_bw() +
  scale_x_continuous(breaks=scales::pretty_breaks(n = 3)) +
  scale_y_continuous(breaks=scales::pretty_breaks(n = 3))
p4 = plot_contraction(
  df.results.b,
  prior_sd = setNames(c(1,
                        rep(0.1, length(unique(df.results.b$variable))-1)),
                      unique(df.results.b$variable))) +
  theme_bw() +
  scale_x_continuous(breaks=scales::pretty_breaks(n = 3)) +
  scale_y_continuous(breaks=scales::pretty_breaks(n = 3))
```

```r
p = ggarrange(p1, p2, p3, p4, labels = "AUTO", ncol = 1, nrow = 4)
annotate_figure(p,
                top = text_grob("Computational faithfulness and model sensitivity",
                face = "bold", size = 14))
```

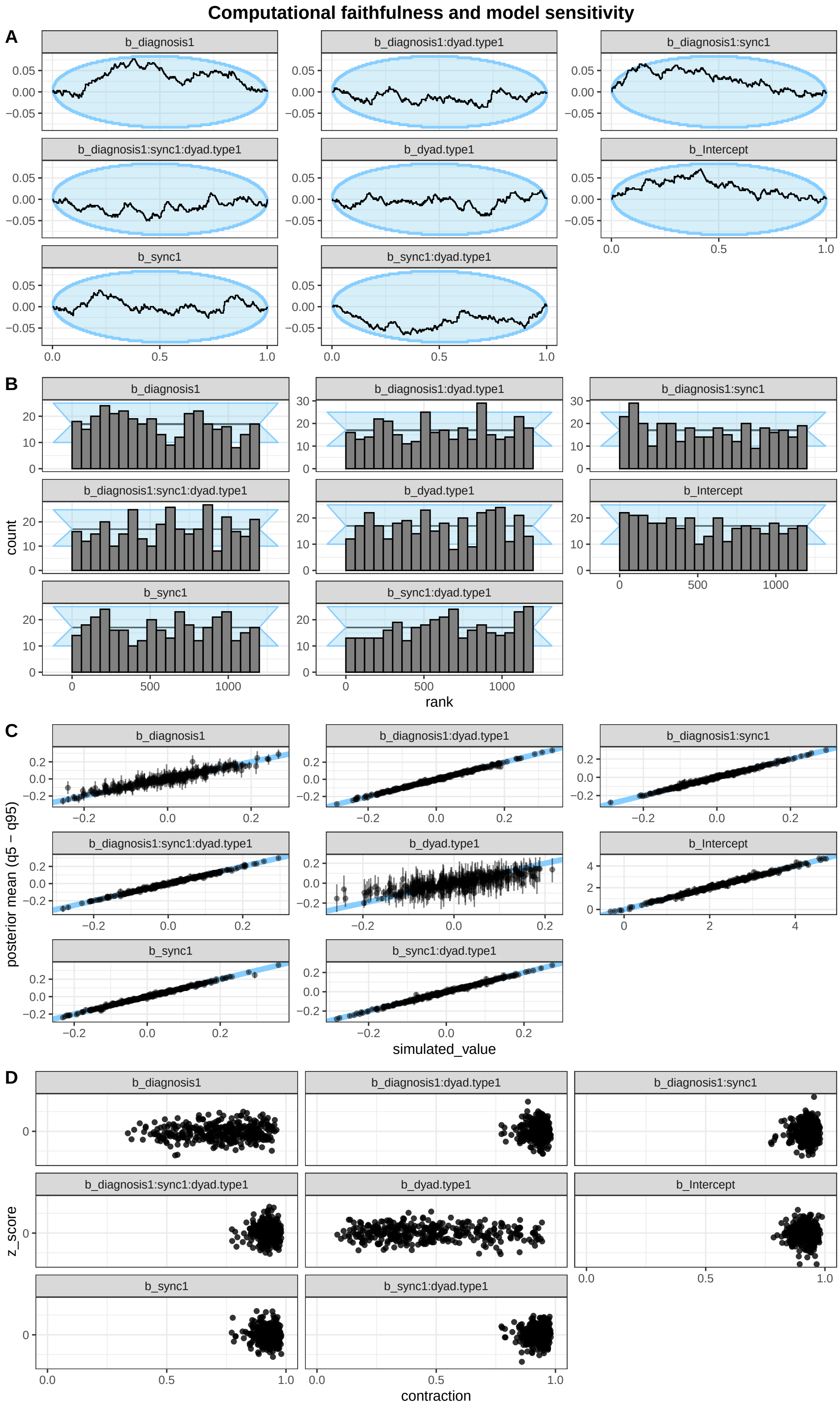

Computational faithfulness and model sensitivity
A
B
C
D
b_diagnosis1
b_diagnosis1:dyad.type1
b_diagnosis1:sync1
b_diagnosis1:sync1:dyad.type1
b_dyad.type1
b_Intercept
b_sync1
b_sync1:dyad.type1
count
rank
posterior mean (q5 − q95)
simulated_value
z_score
contraction

Second, we check the outcome of the SBC. All looks acceptable.

Posterior predictive checks

As the next step, we fit the model and check for issues.

```
# fit the model
set.seed(2496)
m.sac = brm(f.sac,
            df.sac.agg, prior = priors,
            family = poisson,
            iter = iter, warmup = warm,
            backend = "cmdstanr", threads = threading(8),
            file = "m_PESI_sac",
            save_pars = save_pars(all = TRUE)
            )

# in this model, there are no divergent samples
sum(subset(nuts_params(m.sac), Parameter == "divergent__")$Value)
```

```
## [1] 0
```

```
# check that rhats are below 1.01
sum(brms::rhat(m.sac) >= 1.01, na.rm = T)
```

```
## [1] 0
```

```
# and the chains have converged
post.draws = as_draws_df(m.sac)
mcmc_trace(post.draws, regex_pars = "^b_",
           facet_args = list(ncol = 2)) +
  scale_x_continuous(breaks=scales::pretty_breaks(n = 3)) +
  scale_y_continuous(breaks=scales::pretty_breaks(n = 3))
```

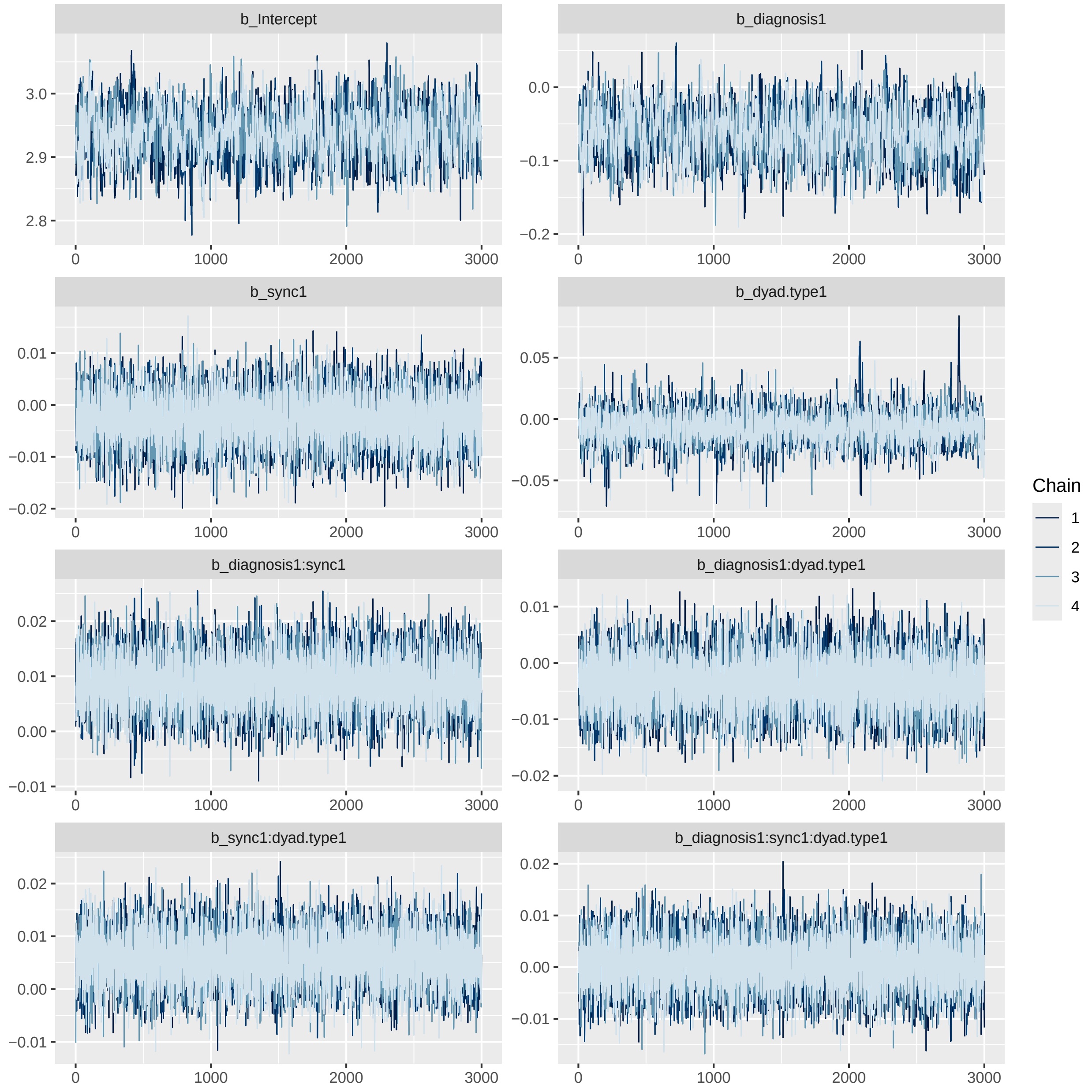

This model has no divergent samples, and no rhat that is higher or equal to 1.01. Therefore, we go ahead and perform our posterior predictive checks.

```
# get the posterior predictions
post.pred = posterior_predict(m.sac, ndraws = nsim)

# check the fit of the predicted data compared to the real data
p1 = pp_check(m.sac, ndraws = nsim) +
  theme_bw() + theme(legend.position = "none")

# distributions of means and sds compared to the real values per group
p2 = ppc_stat_grouped(df.sac.agg$n.sac, post.pred, df.sac.agg$diagnosis) +
  theme_bw() + theme(legend.position = "none")
# ... sync level
p3 = ppc_stat_grouped(df.sac.agg$n.sac, post.pred, df.sac.agg$sync) +
```

```
  theme_bw() + theme(legend.position = "none")
# ... and dyad type
p4 = ppc_stat_grouped(df.sac.agg$n.sac, post.pred, df.sac.agg$dyad.type) +
  theme_bw() + theme(legend.position = "none")

p = ggarrange(p1, p2, p3, p4,
          nrow = 2, ncol = 2, labels = "AUTO")
annotate_figure(p,
                top = text_grob("Posterior predictive checks",
                face = "bold", size = 14))
```

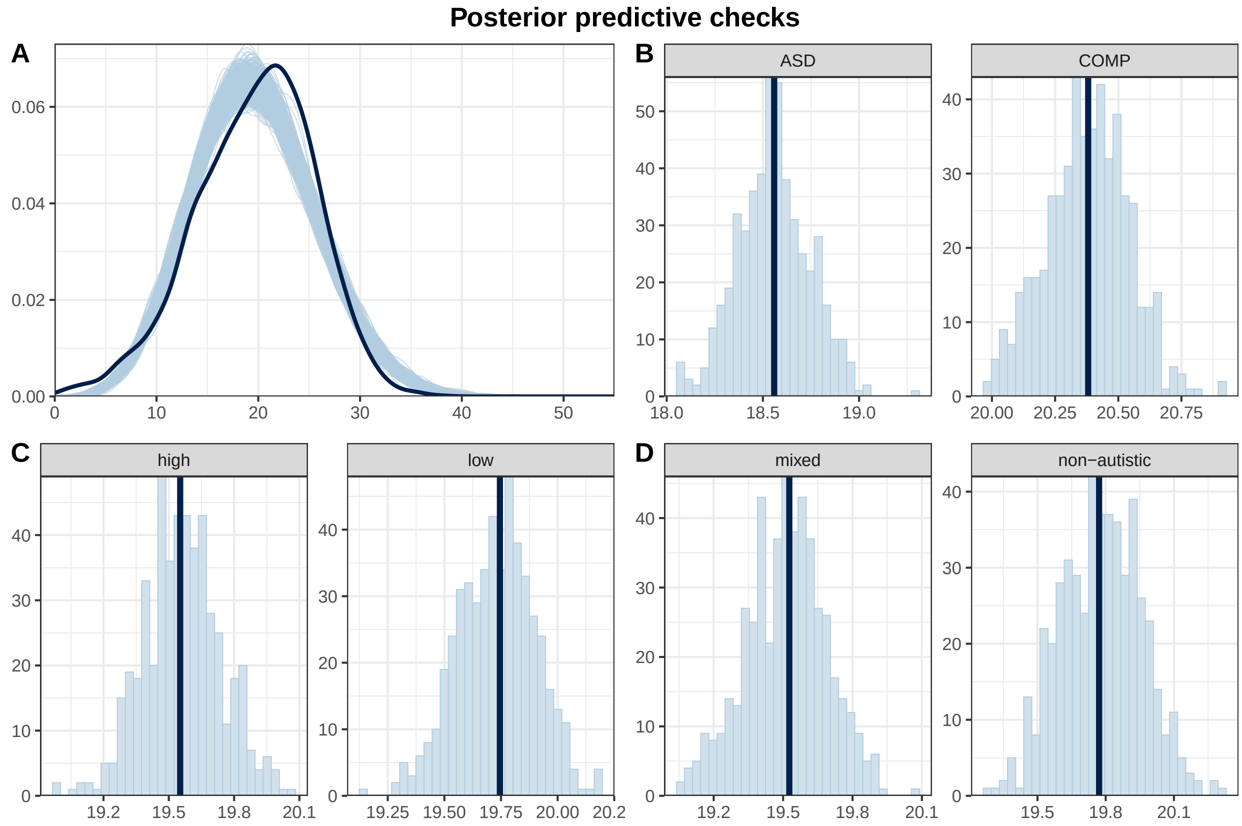

Although the overall shape is not perfect, the means are captured pretty well. Since we could not find a likelihood shape that fit better than this, we accept this model and test our hypotheses.

Model summary

Now, we have a look at the model and its estimates.

```
# print a summary
summary(m.sac)
```

```
##  Family: poisson
##   Links: mu = log
## Formula: n.sac ~ diagnosis * sync * dyad.type + (1 | subID) + (1 | dyad)
##    Data: df.sac.agg (Number of observations: 2392)
##   Draws: 4 chains, each with iter = 4500; warmup = 1500; thin = 1;
##          total post-warmup draws = 12000
```

```
##
## Multilevel Hyperparameters:
## ~dyad (Number of levels: 8)
##               Estimate Est.Error l-95% CI u-95% CI Rhat Bulk_ESS Tail_ESS
## sd(Intercept)     0.03      0.01     0.01     0.06 1.00     3109     5043
##
## ~subID (Number of levels: 46)
##               Estimate Est.Error l-95% CI u-95% CI Rhat Bulk_ESS Tail_ESS
## sd(Intercept)     0.23      0.03     0.18     0.28 1.00     1581     3212
##
## Regression Coefficients:
##                             Estimate Est.Error l-95% CI u-95% CI Rhat Bulk_ESS
## Intercept                       2.94      0.04     2.86     3.01 1.00     1233
## diagnosis1                     -0.06      0.03    -0.13    -0.00 1.00     1538
## sync1                          -0.00      0.00    -0.01     0.01 1.00    12282
## dyad.type1                     -0.01      0.01    -0.03     0.02 1.00     3743
## diagnosis1:sync1                0.01      0.00    -0.00     0.02 1.00    12833
## diagnosis1:dyad.type1          -0.00      0.00    -0.01     0.01 1.00    13114
## sync1:dyad.type1                0.01      0.00    -0.00     0.02 1.00    13274
## diagnosis1:sync1:dyad.type1     0.00      0.00    -0.01     0.01 1.00    12940
##                             Tail_ESS
## Intercept                       2633
## diagnosis1                      2585
## sync1                           8724
## dyad.type1                      4266
## diagnosis1:sync1                8803
## diagnosis1:dyad.type1           8218
## sync1:dyad.type1                9142
## diagnosis1:sync1:dyad.type1     9342
##
## Draws were sampled using sample(hmc). For each parameter, Bulk_ESS
## and Tail_ESS are effective sample size measures, and Rhat is the potential
## scale reduction factor on split chains (at convergence, Rhat = 1).
```

```r
# plot the posterior distributions
post.draws %>%
  select(starts_with("b_")) %>%
  pivot_longer(cols = starts_with("b_"),
               names_to = "coef",
               values_to = "estimate") %>%
  subset(!startsWith(coef, "b_Int")) %>%
  mutate(
    coef = substr(coef, 3, nchar(coef)),
    coef = str_replace_all(coef, ":", " x "),
    coef = str_replace_all(coef, "diagnosis1", "ASD"),
    coef = str_replace_all(coef, "sync1", "high sync"),
    coef = str_replace_all(coef, "dyad.type1", "mixed"),
    coef_order = case_when(
      coef == "ASD" ~ 100,
      coef == "high sync" ~ 99,
      coef == "mixed" ~ 98,
      T ~ 100-nchar(coef)),
    coef = fct_reorder(coef, coef_order)
  ) %>%
```

```
  group_by(coef) %>%
  mutate(
    cred = case_when(
      (mean(estimate) < 0 & quantile(estimate, probs = 0.975) < 0) |
        (mean(estimate) > 0 & quantile(estimate, probs = 0.025) > 0) ~ "credible",
      T ~ "not credible"
    )
  ) %>% ungroup() %>%
  ggplot(aes(x = estimate, y = coef, fill = cred)) +
  geom_vline(xintercept = 0, linetype = 'dashed') +
  ggdist::stat_halfeye(alpha = 0.7) + ylab(NULL) + theme_bw() +
  scale_fill_manual(values = c(credible = c_dark, c_light)) +
  theme(legend.position = "none")
```

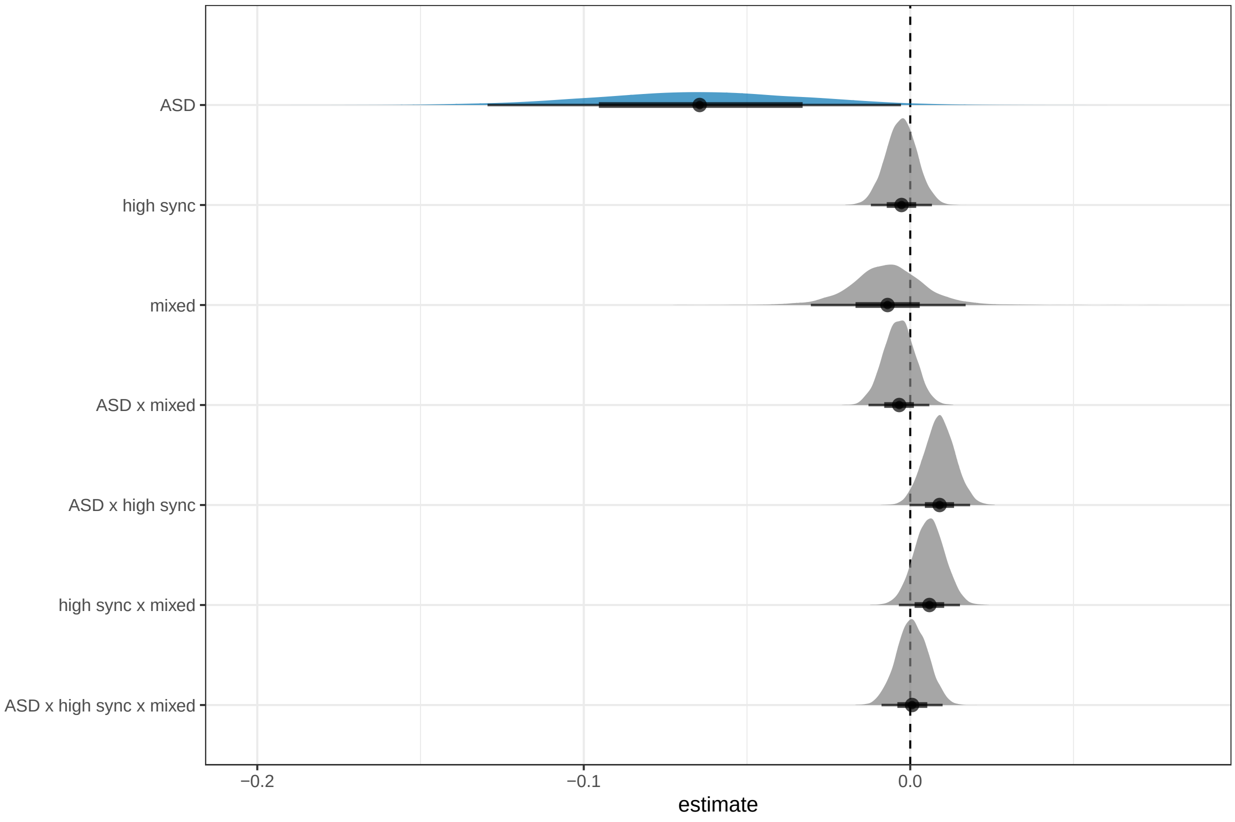


```
df.sac.overall = df.sac.agg %>% group_by(subID, diagnosis) %>%
  summarise(n.sac = mean(n.sac, na.rm = T)) %>%
  ungroup()

# explore difference between observer groups
e.sac = hypothesis(m.sac, "0 > diagnosis1", alpha = 0.025)

# extract predicted differences
df.new = df.sac.agg %>%
  select(diagnosis, sync, dyad.type) %>%
  distinct() %>%
  mutate(
```

```
    condition = paste(diagnosis, sync, dyad.type, sep = "_")
  )

df.ms = as.data.frame(
  fitted(m.sac, summary = F,
              newdata = df.new %>% select(diagnosis, sync, dyad.type),
              re_formula = NA))
colnames(df.ms) = df.new$condition

# calculate our difference columns
df.ms = df.ms %>%
     mutate(
       ASD  = rowMeans(select(., starts_with("ASD")), na.rm = T),
       COMP = rowMeans(select(., starts_with("COM")), na.rm = T),
       COMP_ASD = COMP - ASD
       )
```

In general, observers produced on average 19.31 +- 0.7 saccades per 10-second segment. There were no credible differences between any of the task conditions or interactions. However, autistic observers produced fewer saccades than non-autistic observers ($estimate = 0.06$ [0, 0.13], $posterior\ probability = 0.98$). Specifically, the model predicts that autistic observers produce on average 17.714 [15.936, 19.568] saccades, while comparison observers are predicted to produce 20.151 [18.327, 22.031] saccades on average.

Predicting ratings by gaze behaviour

We also want to see whether across groups gaze behaviour predicted ratings, i.e., focus on specific AOIs led to increased ratings.

SBC

```
# combine data frames
df.com = df.fix %>%
  select(subID, diagnosis, video, sync, dyad, dyad.type, AOI, fix.dur) %>%
  pivot_wider(names_from = AOI, values_from = fix.dur) %>%
  merge(., df.sac.agg) %>%
  merge(., df) %>%
  group_by(subID, diagnosis, sync, dyad, dyad.type) %>%
  summarise(
    head  = mean(head),
    hand  = mean(hand),
    body  = mean(body),
    n.sac = mean(n.sac),
    rating.confirmed = mean(rating.confirmed, na.rm = T)
  ) %>% ungroup() %>%
  # scale the predictors
  mutate(
    sn.sac = scale(n.sac),
    s.head = scale(head),
    s.hand = scale(hand),
    s.body = scale(body)
  ) %>% drop_na()

# set formula
code   = "PESI_com"
```

```r
f.com = brms::bf(rating.confirmed ~ sn.sac + s.head + s.hand + s.body +
                   (1 | subID) + (1 | dyad))

# set weakly informed priors
priors = c(
  prior(normal(50,  10), class = Intercept),
  prior(normal(10,  10), class = sigma),
  prior(normal(0,   10), class = sd),
  prior(normal(0,   5), class = b)
)

if (file.exists(file.path(cache_dir, paste0("df_res_", code, ".rds")))) {
  # load in the results of the SBC
  df.results = readRDS(file.path(cache_dir, paste0("df_res_", code, ".rds")))
  df.backend = readRDS(file.path(cache_dir, paste0("df_div_", code, ".rds")))
  dat        = readRDS(file.path(cache_dir, paste0("dat_", code, ".rds")))
} else {
  # set the seed
  set.seed(2474)
  # create the data
  gen  = SBC_generator_brms(f.com, data = df.com, prior = priors,
                            thin = 50, warmup = 20000, refresh = 2000
  )
  dat  = generate_datasets(gen, nsim)
  saveRDS(dat, file = sprintf("%s/dat_%s.rds", cache_dir, code))

  # perform the SBC
  bck = SBC_backend_brms_from_generator(gen, chains = 4, thin = 1,
                                        warmup = warm, iter = iter,
                                        inits = 0.1)
  res = compute_SBC(dat, bck,
                    cache_mode = "results",
                    cache_location = file.path(cache_dir, sprintf("res_%s", code)))
  # save the results dataframes
  df.results = res$stats
  df.backend = res$backend_diagnostics
  saveRDS(df.results, file = file.path(cache_dir, paste0("df_res_", code, ".rds")))
  saveRDS(df.backend, file = file.path(cache_dir, paste0("df_div_", code, ".rds")))

}
```

Again, we start by investigating the rhats and the number of divergent samples. This shows that 4 of 500 simulations had at least one parameter that had an rhat of at least 1.05, and 83 models had divergent samples (mean number of samples of the simulations with divergent samples: 3.37). Again, we will look out for divergence issues in the final model.

Next, we can plot the simulated values to perform prior predictive checks.

```r
# create a matrix out of generated data
dvname = gsub(" ", "", gsub("[\\|~].*", "", f.com)[1])
dvfakemat = matrix(NA, nrow(dat[['generated']][[1]]), length(dat[['generated']]))
for (i in 1:length(dat[['generated']])) {
  dvfakemat[,i] = dat[['generated']][[i]][[dvname]]
}
truePars = dat$variables
```

```
# set very large data points to a value of 100 and small to 0
dvfakematH = dvfakemat;
dvfakematH[dvfakematH > 100] = 100
dvfakematH[dvfakematH < 0]   = 0
# compute one histogram per simulated data-set
breaks = seq(0, 100, length.out = 101)
binwidth = breaks[2] - breaks[1]
histmat = matrix(NA, ncol = nrow(truePars) + binwidth, nrow = length(breaks)-1)
for (i in 1:nrow(truePars)) {
  histmat[,i] = hist(dvfakematH[,i], breaks = breaks, plot = F)$counts
}
# for each bin, compute quantiles across histograms
probs = seq(0.1, 0.9, 0.1)
quantmat= as.data.frame(matrix(NA, nrow=dim(histmat)[1], ncol = length(probs)))
names(quantmat) = paste0("p", probs)
for (i in 1:dim(histmat)[1]) {
  quantmat[i,] = quantile(histmat[i,], p = probs, na.rm = T)
}
quantmat$x = breaks[2:length(breaks)] - binwidth/2 # add bin mean
p1 = ggplot(data = quantmat, aes(x = x)) +
  geom_ribbon(aes(ymax = p0.9, ymin = p0.1), fill = c_light) +
  geom_ribbon(aes(ymax = p0.8, ymin = p0.2), fill = c_light_highlight) +
  geom_ribbon(aes(ymax = p0.7, ymin = p0.3), fill = c_mid) +
  geom_ribbon(aes(ymax = p0.6, ymin = p0.4), fill = c_mid_highlight) +
  geom_line(aes(y = p0.5), colour = c_dark, linewidth = 1) +
  labs(title = "Prior predictive distribution", y = "", x = "number of saccades") +
  theme_bw()
p1
```

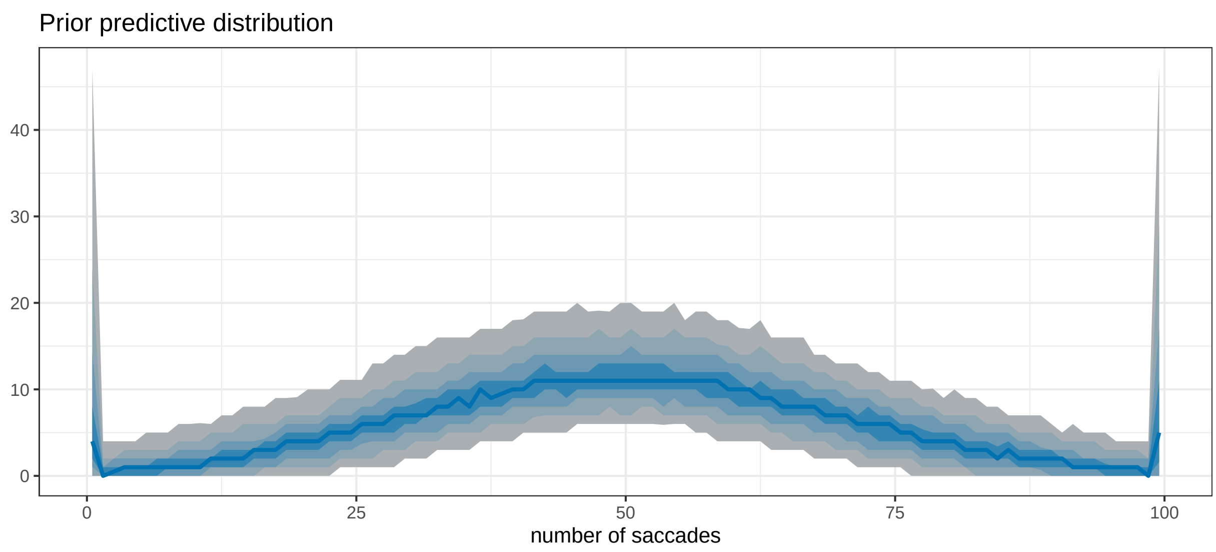

The overall shape again looks acceptable, even though there are some impossible simulated values.

```
# get simulation numbers with issues
rank = max(df.results$max_rank)
check = merge(df.results %>%
    group_by(sim_id) %>%
```

```
      summarise(
        rhat = max(rhat, na.rm = T),
        mean_rank = mean(max_rank)
        ) %>%
    filter(rhat >= 1.05 | mean_rank != rank),
  df.backend %>% filter(n_divergent > 0), all = T)

# plot SBC with functions from the SBC package focusing on population-level parameters
df.results.b = df.results %>%
  filter(substr(variable, 1, 2) == "b_") %>%
  filter(!(sim_id %in% check$sim_id))
p1 = plot_ecdf_diff(df.results.b) + theme_bw() + theme(legend.position = "none") +
  scale_x_continuous(breaks=scales::pretty_breaks(n = 3)) +
  scale_y_continuous(breaks=scales::pretty_breaks(n = 3))
p2 = plot_rank_hist(df.results.b, bins = 20) + theme_bw() +
  scale_x_continuous(breaks=scales::pretty_breaks(n = 3)) +
  scale_y_continuous(breaks=scales::pretty_breaks(n = 3))
p3 = plot_sim_estimated(df.results.b, alpha = 0.5) + theme_bw() +
  scale_x_continuous(breaks=scales::pretty_breaks(n = 3)) +
  scale_y_continuous(breaks=scales::pretty_breaks(n = 3))
p4 = plot_contraction(
  df.results.b,
  prior_sd = setNames(c(10,
                        rep(5, length(unique(df.results.b$variable))-1)),
                      unique(df.results.b$variable))) +
  theme_bw() +
  scale_x_continuous(breaks=scales::pretty_breaks(n = 3)) +
  scale_y_continuous(breaks=scales::pretty_breaks(n = 3))

p = ggarrange(p1, p2, p3, p4, labels = "AUTO", ncol = 1, nrow = 4)
annotate_figure(p,
                top = text_grob("Computational faithfulness and model sensitivity",
                face = "bold", size = 14))
```

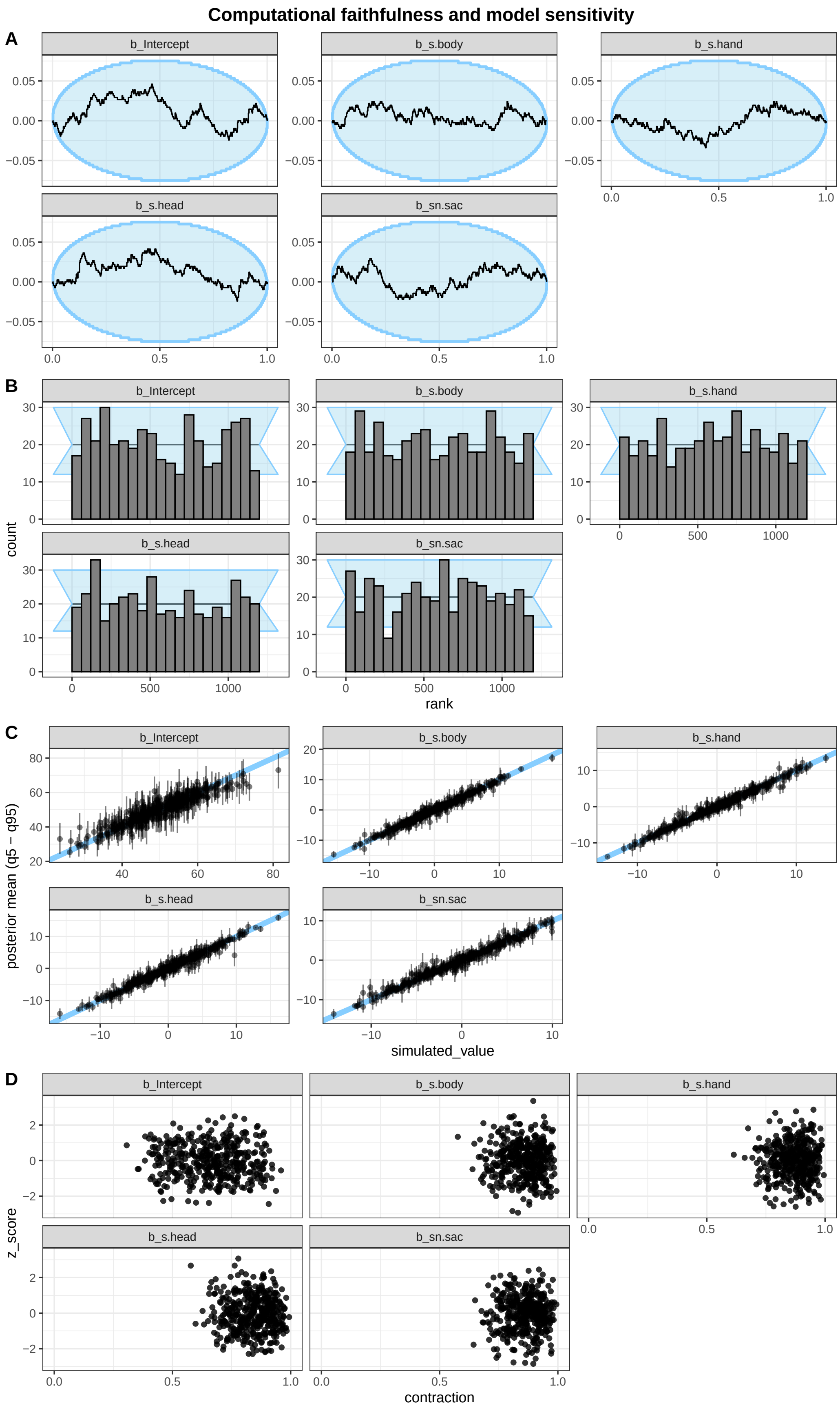
Computational faithfulness and model sensitivity
A
b_Intercept
b_s.body
b_s.hand
b_s.head
b_sn.sac
B
count
rank
C
posterior mean (q5 – q95)
simulated_value
D
z_score
contraction

Second, we check the outcome of the SBC. All looks acceptable.

Posterior predictive checks

As the next step, we fit the model and check for issues.

```
# fit the model
set.seed(2496)
m.com = brm(f.com,
            df.com, prior = priors,
            iter = iter, warmup = warm,
            backend = "cmdstanr", threads = threading(8),
            file = "m_PESI_com",
            save_pars = save_pars(all = TRUE)
            )


# in this model, there are no divergent samples
sum(subset(nuts_params(m.com), Parameter == "divergent__")$Value)
```

```
## [1] 0
```

```
# check that rhats are below 1.01
sum(brms::rhat(m.com) >= 1.01, na.rm = T)
```

```
## [1] 0
```

```
# and the chains have converged
post.draws = as_draws_df(m.com)
mcmc_trace(post.draws, regex_pars = "^b_",
           facet_args = list(ncol = 2)) +
  scale_x_continuous(breaks=scales::pretty_breaks(n = 3)) +
  scale_y_continuous(breaks=scales::pretty_breaks(n = 3))
```

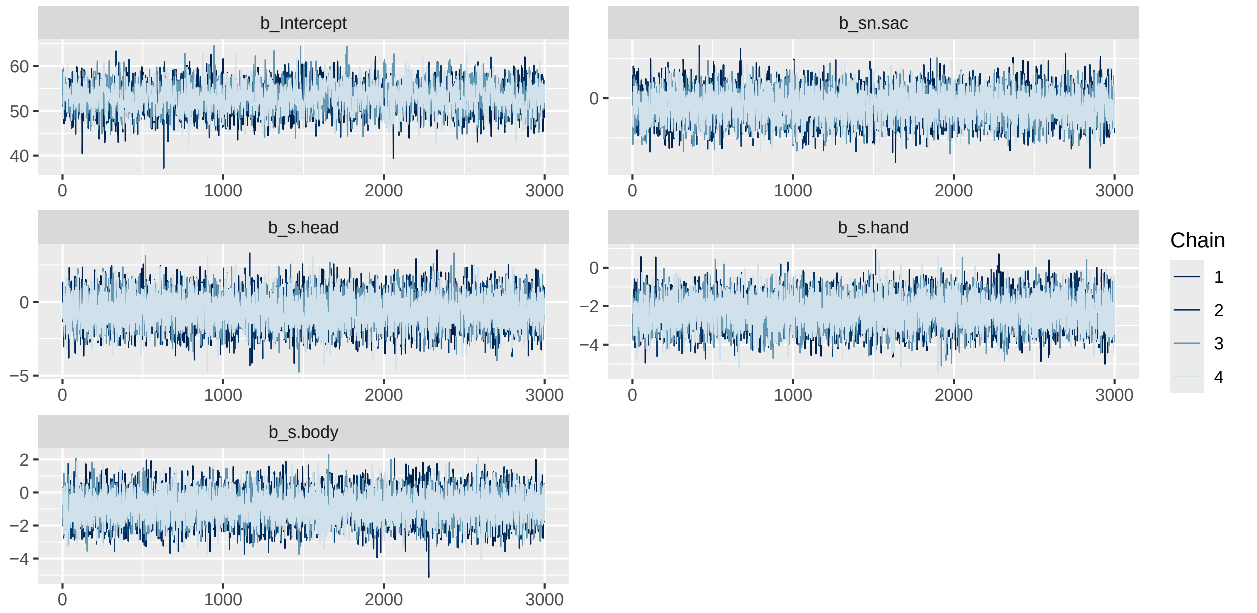

This model has no divergent samples, and no rhat that is higher or equal to 1.01. Therefore, we go ahead and perform our posterior predictive checks.

```r
# get the posterior predictions
post.pred = posterior_predict(m.com, ndraws = nsim)

# check the fit of the predicted data compared to the real data
p1 = pp_check(m.com, ndraws = nsim) +
  theme_bw() + theme(legend.position = "none")

annotate_figure(p1,
                top = text_grob("Posterior predictive checks",
                face = "bold", size = 14))
```

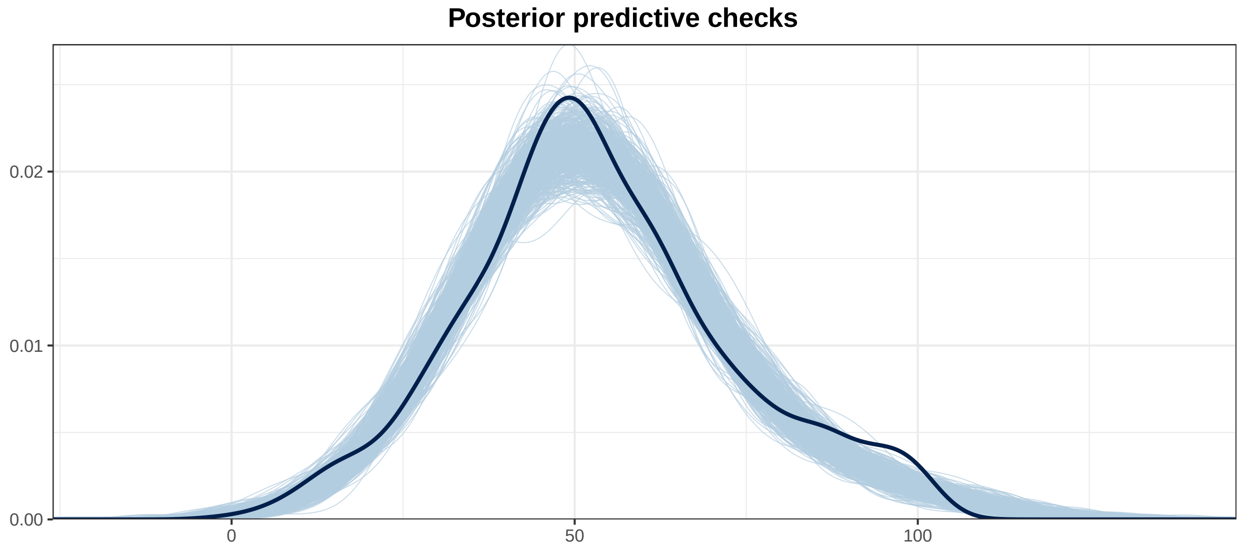

The predicted values in light blue fit the actual values in dark blue quite well.

Model summary

Now, we have a look at the model and its estimates.

```r
# print a summary
summary(m.com)
```

```
##  Family: gaussian
##   Links: mu = identity; sigma = identity
## Formula: rating.confirmed ~ sn.sac + s.head + s.hand + s.body + (1 | subID) + (1 | dyad)
##    Data: df.com (Number of observations: 667)
##   Draws: 4 chains, each with iter = 4500; warmup = 1500; thin = 1;
##          total post-warmup draws = 12000
##
## Multilevel Hyperparameters:
## ~dyad (Number of levels: 8)
##               Estimate Est.Error l-95% CI u-95% CI Rhat Bulk_ESS Tail_ESS
## sd(Intercept)     6.19      1.96     3.42    10.93 1.00     4201     6701
##
## ~subID (Number of levels: 42)
##               Estimate Est.Error l-95% CI u-95% CI Rhat Bulk_ESS Tail_ESS
## sd(Intercept)    12.46      1.54     9.83    15.81 1.00     3046     4538
##
```

```
## Regression Coefficients:
##           Estimate Est.Error l-95% CI u-95% CI Rhat Bulk_ESS Tail_ESS
## Intercept    52.93      2.94    47.08    58.70 1.00     2782     4446
## sn.sac       -0.46      0.91    -2.27     1.34 1.00     7671     8319
## s.head       -0.56      1.05    -2.60     1.50 1.00     8783     8423
## s.hand       -2.27      0.80    -3.83    -0.70 1.00    10539     9438
## s.body       -0.85      0.87    -2.56     0.85 1.00    10523     8832
##
## Further Distributional Parameters:
##       Estimate Est.Error l-95% CI u-95% CI Rhat Bulk_ESS Tail_ESS
## sigma    14.00      0.40    13.24    14.81 1.00    14538     8668
##
## Draws were sampled using sample(hmc). For each parameter, Bulk_ESS
## and Tail_ESS are effective sample size measures, and Rhat is the potential
## scale reduction factor on split chains (at convergence, Rhat = 1).
```

```
# plot the posterior distributions
post.draws %>%
  select(starts_with("b_")) %>%
  pivot_longer(cols = starts_with("b_"),
               names_to = "coef",
               values_to = "estimate") %>%
  subset(!startsWith(coef, "b_Int")) %>%
  mutate(
    coef = substr(coef, 3, nchar(coef))
  ) %>%
  group_by(coef) %>%
  mutate(
    cred = case_when(
      (mean(estimate) < 0 & quantile(estimate, probs = 0.975) < 0) |
        (mean(estimate) > 0 & quantile(estimate, probs = 0.025) > 0) ~ "credible",
      T ~ "not credible"
    )
  ) %>% ungroup() %>%
  ggplot(aes(x = estimate, y = coef, fill = cred)) +
  geom_vline(xintercept = 0, linetype = 'dashed') +
  ggdist::stat_halfeye(alpha = 0.7) + ylab(NULL) + theme_bw() +
  scale_fill_manual(values = c(credible = c_dark, c_light)) +
  theme(legend.position = "none")
```

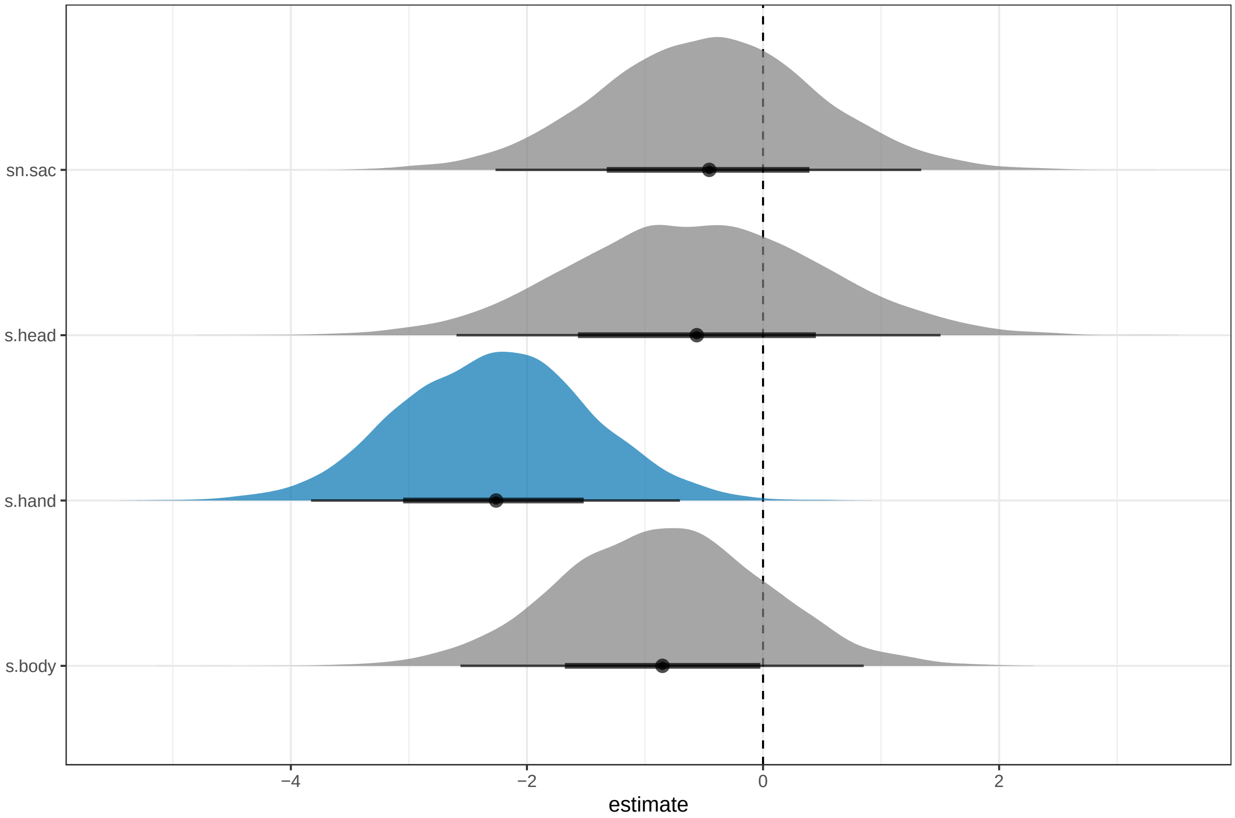

Predicting comfortability ratings with gaze behaviour revealed that higher comfortability impressions were associated with reduced dwell times on the hands (Scaled dwell times on hands: *estimate* = -2.27 [-3.83, -0.7]) but no other gaze behaviour.
